# 5-Arylideneimidazolones with Amine at Position 3 as Potential Antibiotic Adjuvants against Multidrug Resistant Bacteria

**DOI:** 10.3390/molecules24030438

**Published:** 2019-01-26

**Authors:** Aneta Kaczor, Karolina Witek, Sabina Podlewska, Joanna Czekajewska, Annamaria Lubelska, Ewa Żesławska, Wojciech Nitek, Gniewomir Latacz, Sandrine Alibert, Jean-Marie Pagès, Elżbieta Karczewska, Katarzyna Kieć-Kononowicz, Jadwiga Handzlik

**Affiliations:** 1 Department of Technology and Biotechnology of Drugs, Jagiellonian University, Medical College, Medyczna 9, 30-688 Cracow, Poland; aneta.kaczor@student.uj.edu.pl (A.K.); karolina.witek@uj.edu.pl (K.W.); smusz@if-pan.krakow (S.P.); annamarialubelska@outlook.com (A.L.); glatacz@cm-uj.krakow.pl (G.L.); mfkonono@cyf-kr.edu.pl (K.K.-K.); 2 Department of Pharmaceutical Microbiology; Jagiellonian University, Medical College, Medyczna 9, 30-688 Cracow, Poland; j.czekajewska@uj.edu.pl (J.C.); elzbieta.karczewska@uj.edu.pl (E.K.); 3 Aix Marseille Univ, INSERM, SSA, MCT, FAC PHARM, 27 Bd Jean Moulin, 13005 Marseille, France; sandrine.alibert@univ-amu.fr (S.A.); Jean-Marie.PAGES@univ-amu.fr (J.-M.P.); 4 Department of Medicinal Chemistry, Institute of Pharmacology, Polish Academy of Sciences, Smętna 12, 31-343 Cracow, Poland; 5 Department of Chemistry, Institute of Biology, Pedagogical University of Cracow, Podchorążych 2, 30-084 Cracow, Poland; ewa.zeslawska@up.krakow.pl; 6 Faculty of Chemistry, Jagiellonian University, Gronostajowa 2, 30-387 Cracow, Poland; nitek@chemia.uj.edu.pl

**Keywords:** multi-drug resistance, Efflux pump, MRSA, *Staphylococcus aureus*, *Enterobacter aerogenes*, 5-arylideneimidazolones, microdilution assays, RTE studies, allosteric modulation, ADMET in vitro

## Abstract

Searching for new chemosensitizers of bacterial multidrug resistance (MDR), chemical modifications of (Z)-5-(4-chlorobenzylidene)-2-(4-methylpiperazin-1-yl)-3H-imidazol-4(5H)-one (**6**) were performed. New compounds (**7**–**17**), with fused aromatic rings at position 5, were designed and synthesized. Crystallographic X-ray analysis proved that the final compounds (**7**–**17**) were substituted with tertiary amine-propyl moiety at position 3 and primary amine group at 2 due to intramolecular Dimroth rearrangement. New compounds were evaluated on their antibiotic adjuvant properties in either Gram-positive or Gram-negative bacteria. Efflux pump inhibitor (EPI) properties towards the AcrAB-TolC pump in *Enterobacter aerogenes* (EA289) were investigated in the real-time efflux (RTE) assay. Docking and molecular dynamics were applied to estimate an interaction of compounds **6**–**17** with penicillin binding protein (PBP2a). In vitro ADME-Tox properties were evaluated for compound **9**. Most of the tested compounds reduced significantly (4-32-fold) oxacillin MIC in highly resistant MRSA HEMSA 5 strain. The anthracene-morpholine derivative (**16**) was the most potent (32-fold reduction). The tested compounds displayed significant EPI properties during RTE assay (37–97%). The naphthyl-methylpiperazine derivative **9** showed the most potent “dual action” of both oxacillin adjuvant (MRSA) and EPI (*E. aerogenes*). Molecular modeling results suggested the allosteric mechanism of action of the imidazolones, which improved binding of oxacillin in the PBP2a active site in MRSA.

## 1. Introduction

Multidrug resistance (MDR) is a non-susceptible phenotype of a given microorganism to antimicrobial drugs that belong to different chemical classes and have different mechanisms of action [1,2]. Bacterial MDR is nowadays a global problem that severely affects the therapy of infectious diseases. Amongst Gram-positive bacteria that pose a particular threat to human health is *Staphylococcus aureus* [3]. Of great concern is the increased prevalence of methicillin-resistant *S. aureus* (MRSA) strains that lack susceptibility to nearly all β-lactams, which are considered the most relevant class of antibiotics due to their bactericidal activity and excellent safety profile [4,5,6]. Moreover, MRSA strains have an extraordinary versatility to develop resistance to macrolides, lincosamides, streptogramin B, and many other classes of antibacterial drugs, including last resort antibiotics such as vancomycin, linezolid and daptomycin [5,7]. Consequently, infections caused by MRSA strains are often difficult to treat and are associated with an increased risk of treatment failure [8]. Indeed, MRSA is listed by the World Health Organization as one of the most problematic bacterial pathogens, for which available treatment options are constantly decreasing [9]. MDR strains use various ways to circumvent the harmful effects of antibacterial agents, including modification of the drug target, production of enzymes which degrade the antibiotic molecules, over-expression of efflux pumps, and reduction of envelope permeability [1,10,11]. Nevertheless, the modification of PBP (Penicillin Binding Protein) to PBP2a (also called PBP2′), a target for β-lactam antibiotics, seems to be the most common resistance mechanism in *S. aureus* [12,13]. In Gram-negative bacteria, such as *Enterobacter aerogenes* and *Escherichia coli*, the reduction of antibiotic susceptibility is often reported with the overproduction of efflux pumps that expel toxic substances out of bacterial cell. This mechanism is associated with decreased membrane permeability, reducing the intracellular concentration of antimicrobial drugs and promoting bacterial survival [10,14]. The tripartite system AcrAB-TolC is the well-characterized efflux pump that belongs to resistance-nodulation-cell division (RND) transporters in Gram-negative bacteria. This system is of special clinical importance as its modulation rejuvenates the effectiveness of multiple antibacterial drugs, e.g., β-lactams, fluoroquinolones, and tetracyclines [14,15]. According to “the state of art”, various strategies to combat MDR bacterial strains were proposed, e.g., chemical modifications in order to improve the structure-activity relationship (SAR) of available antibiotics and the search for so-called antibiotic adjuvants [13,16]. Antibiotic adjuvants, or chemosensitizers, are compounds that have the ability to restore the antibacterial activity of antibiotics towards resistant strains of bacterial pathogens. These molecules are supposed to potentiate antibiotics efficacy and display a negligible intrinsic antibacterial action [17,18,19]. The expected advantage of pairing an antibiotic with an adjuvant is a low rate of new emerging bacterial resistance in such combinations. A classic example of the strategy, based on the application of antibiotic potentiators in the therapy of infectious diseases, is the combination of β-lactam antibiotic amoxicillin with β-lactamase inhibitor clavulanic acid, used in clinical treatment since 1981 [20,21]. Taking into consideration increasing prevalence of MDR strains and the scarcity of new antibiotic classes, the search for new compounds able to block bacterial mechanisms of resistance seems to be a promising strategy that could help to extend the life span of these life-saving drugs [22,23].

**Figure 1:**
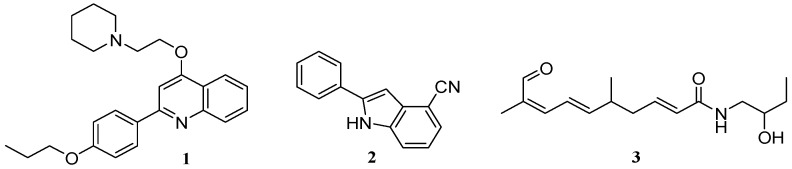
Chemical variety of compounds able to restore antibiotic efficiency in staphylococci (**1–3**).

Regarding MDR staphylococci, there are numerous reports describing structurally unrelated compounds possessing adjuvant-like properties, i.e., quinolone (**1**) and indole derivatives (**2**), chalcones, citral amides and alkenamides (**3**, Figure 1) [11,24].

On the grounds of similarity of hydantoin core to a part of PAβN, which is a well-known efflux pump inhibitor, modifications in this group were carried out to obtain potential adjuvant activity. Further studies indicate imidazolone derivatives with amphiphilic properties as the most promising [10].

**Figure 2:**
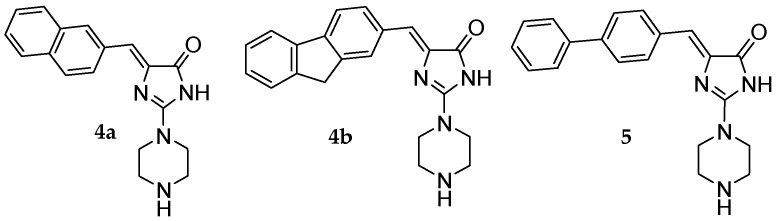
Active 5-arylideneimidazolones (**4a**, **4b**, **5**) found previously.

In our previous research on 5-arylideneimidazolone derivatives, unsubstituted piperazine at position 2, displayed high potency to restore the efficacy of β-lactams and fluoroquinolone antibiotic ciprofloxacin in MDR strains of *S. aureus* (**4a** and **4b**, Figure 2) [25]. Furthermore, the compounds with arylidene moiety at position 5 and amine at position 2 (**4a**, **4b**, **5**) were able to re-sensitize MDR strains of Gram-negative bacteria for selected antibiotics and displayed strong efflux pump inhibitory (EPI) properties towards AcrAB-TolC. Compound **5** caused the highest reduction (32-fold) of rifampin MIC (minimal inhibitory concentration) and an 8-fold reduction of a few antibiotics MIC values: oxacillin, chloramphenicol, linezolid and clarytromycin [15].

On the basis of those interesting results obtained for 5-arylideneimidazolones with an unsubstituted piperazine moiety, we decided to explore their methylpiperazine analogues starting from *p*-chlorobenzylideneimidazolone **6** (Table 1). The compound **6** gave yellow crystals and was used for X-ray crystallographic analysis, as described earlier [26].

Taking into consideration comprehensive structural data for the compound **6**, which also initiated a new group of methylpiperazine derived 5-arylideneimidazolones, the compound was selected as a lead structure for further modifications in search for new antibiotic adjuvants performed within this study. The modifications aimed to introduce a spacer between imidazolone and the methylpiperazine at position 2 as well as an extension of aromatic area at position 5 of imidazolone (**7′**–**17′**, part A, Table 1), in respect to the chemical structure of favorable compounds (**4a**, **4b** and **5**) found previously [15,25]. However, the structures of the new desired compounds turned out to be unexpectedly different (**7**–**17**, part B, Table 1). In this context, the lead structure and its derivatives **7**–**17** were the main subject of this study. Thus, synthesis of the new series and X-ray structural consideration was performed. Final products were tested in microbiological assays for their antibiotic adjuvant potency as well as in molecular modelling to determine the potential mechanism of action towards PBP2a. For selected compounds, EPI properties were examined. One of the most active compounds (**9**) was selected for ADME-Tox assays in vitro. Based on the results obtained, structure-activity relationship (SAR) was discussed.

## 2. Results and Discussion

### 2.1. Chemical Synthesis

**Scheme 1:**
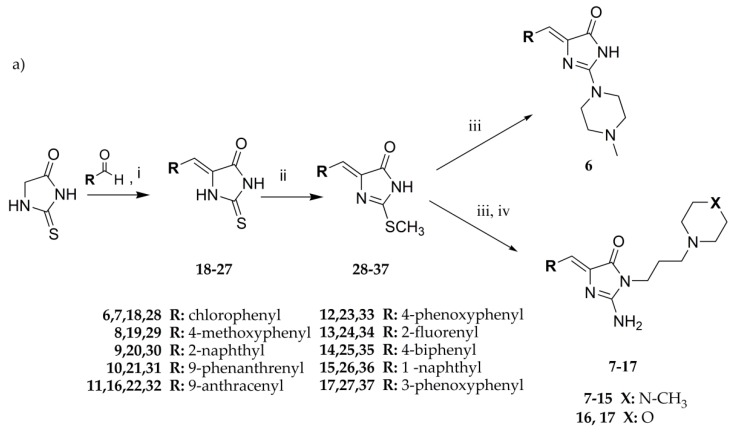

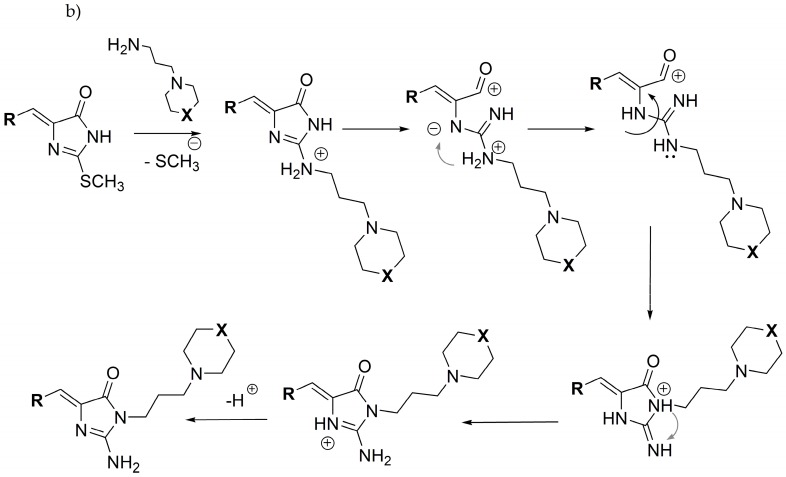
Synthesis route for compounds **6**–**17**; (**a**) (i), CH_3_COONa, CH_3_COOH, reflux, 7 h; (ii) CH_3_I, EtONa, rt, 24 h; (iii) proper amine, EtOH, 120–150 °C, 15 min (iv) Dimroth rearrangement; (**b**) Mechanism of Dimroth rearrangement.

Synthesis of compounds **6**–**17** was performed according to the Scheme 1. Details of synthesis for **6** were described elsewhere [26]. Compounds **7**–**17** were synthesized within three stages, starting from corresponding initial steps to obtain intermediates **18**–**27** and **28**–**37**, respectively (Scheme 1). In the first step (Scheme 1a), Knoevenagel condensation between thiohydantoin and aromatic aldehydes was performed to give 5-arylidene-2-thiohydantoins **18**–**27**. Then, the S-methylation of **18**–**27** using iodomethane in basic sodium ethylate condition provided S-methyl intermediates (**28**–**37**). In the next step, solvent-free reactions of S-methylimidazolones (**28**–**37**) with proper commercial amines were carried out in melting condition to give different substitution at imidazolone ring, depending on amine properties. In the case of the secondary amine (*N*-methylpiperazine), the product **6** of simple replacement of S-methyl group at position 2 was a result. In contrast, the similar process performed by the use of primary amines caused a Dimroth rearrangement (Scheme 1b) [27] to give the final products (**7**–**17**) with primary amine moiety at position 2, and an aminealkyl substituent at position 3 of the 5-arylideneimidazolone pattern (**7**–**17**).

### 2.2. Crystallographic Studies

**Figure 3:**
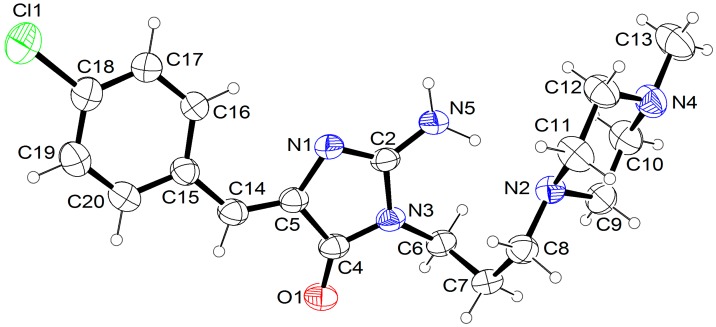
Atom numbering scheme of compound **7**. Displacement ellipsoids are drawn at the 30% probability level.

The overall shape of molecule **7** with atom numbering is presented in Figure 3. The 5-(p-chlorobenzylidene)-imidazolone fragment is almost planar. The angle between the planes of these two rings is 12.36(5)°. The molecule possesses a double bond C5=C14 and can form two geometric isomers (Z or E). In the crystal structure of **7** the Z isomer is observed. This isomer also occurred in other crystal structures, determined earlier, containing 5-(p-chlorobenzylidene)-imidazolone moiety [26,28]. We have conducted a search of the Cambridge Structural Database [29] and in all crystal structures containing 5-arylideneimidazolone fragments only the Z isomer was noted. The molecule of the investigated compound adopts a bent conformation, with the torsion angles of linker: N3-C6-C7-C8 = −60.3(2)°, C6-C7-C8-N2 = −51.7(2)°. This conformation is imposed by the intramolecular hydrogen bond N5-H5B···N2 (Table 2). The piperazine ring has chair conformation with equatorial positions of substituents at nitrogen atoms.

**Figure 4:**
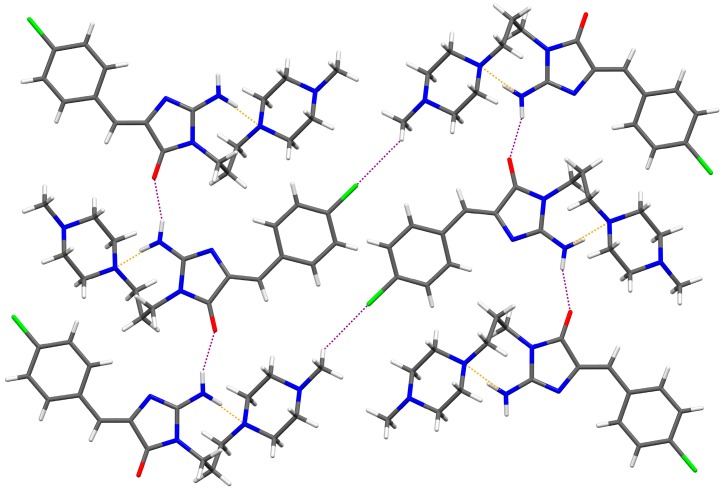
Partial packing view of compound **7** along [100] direction. Dashed lines indicate hydrogen bonds: orange—intramolecular, purple—intermolecular.

The crystal network in the studied structure can be characterized by N5-H5A···O1 intermolecular hydrogen bonds, creating a chain C(6) along [001] direction [30]. Furthermore, the C13-H13B···Cl1 interactions between the chains, which lead to the formation of layers (Figure 4), are observed (Table 2).

Results of the crystallographic studies for compound **7** allowed us to identify the Dimroth rearrangement and real structure of the series of compounds (**7**–**17**). It was difficult to recognize this structure only on the basis of the results of both, NMR and LC-MS analyses, since the molecular mass and number of protons is the same in the expected compound **7′** and the real Dimroth product (**7**). Before the crystallography results, we had identified the bright singlet (7.80 ppm) occurring in ^1^H-NMR spectrum of basic form of **7** (see Supplementary) as that coming from 2H of guanidine-like fragment formed by N1 (in position 1), C2-NH- (position 2) and N3-H (position 3) of the imidazolone ring. These two acid protons could give one bright singlet due tautomerism. The X-ray results have undermined that wrong hypothesis, showing that this peak of two protons comes from the NH_2_ group placed at position 2. This peak occurred in the basic form, while it was exchanged into a very bright (almost plane) singlet at 9–10 ppm (typical for N1-H protons of 5-arylidene-2-thiohydantoin), which indicated a probable transformation of NH_2_ (at C2) into the tautomeric form of N1-H and C2 = NH (see Supplementary) in the case of the hydrochloric form of **7**. Based on the results of X-ray analysis for **7**, it is also distinctly seen that the triplet at ~3.50 ppm comes from N3-CH_2_ protons because similar shifts were observed for N3-CH_2_ protons of N3-propyl-substituted hydantoin derivatives [25], whereas this shift is too high for any propyl-CH_2_ protons in the case of an unrearranged compound (**7′**). Thus, the crystallography studies allowed us to right assign ^1^H-NMR peaks to the suitable protons of the Dimroth rearrangement product. Similar trends that occurred in the ^1^H-NMR spectra of the rest of the series confirm the Dimroth rearrangement structure for the rest of the compounds (**8**–**17**), although the hydrochloric form and more extended aromatic areas disturb the detection of such clear and regular peaks as those for the phenyl symmetric-substituted **7** in its basic form.

### 2.3. Biological Assays

All compounds were investigated on their adjuvant action in the reference and MDR Gram-positive *Staphylococcus aureus* strains. Selected compounds (**9**–**13**, **16**, **17**) were also tested in Gram-negative *E. aerogenes* strains, by employing both the microdilution and the real-time efflux (RTE) assays. In the first step of the study, direct antibacterial activity of compounds against aforementioned bacteria was evaluated. Next, the influence of compounds (at the concentrations corresponding to 25% of their intrinsic MICs) on MICs of antibiotics was investigated. Finally, real-time efflux (RTE) assays were performed in order to determine efflux pump inhibitory properties of compounds towards AcrAB-TolC in *E. aerogenes*.

#### 2.3.1. Direct Antibacterial Activity

##### Gram-positive S. aureus

Initially, 12 arylideneimidazolone derivatives were evaluated in vitro for their intrinsic antibacterial effect against two strains of *S. aureus*: the reference strain *S. aureus* ATCC 25923 and extremely-drug resistant (XDR) MRSA HEMSA 5 clinical isolate. This step of the study was necessary for: (i) elucidation whether molecules tested are devoid of antistaphylococcal activity and thus cannot become antimicrobial agents by themselves; and (ii) determination of the concentrations of compounds suitable for the further assay on their antibiotic adjuvant potency. In addition, the antistaphylococcal efficacy of oxacillin, which was paired with compounds tested, in the following assays was also assessed (Table 3).

The results obtained for arylideneimidazolone derivatives **6**–**17** have demonstrated that the compounds did not exhibit any notable antibacterial activity against *S. aureus* strains used in the study. Among all compounds tested, the lowest MIC value was determined for the compound **13**, which inhibited the growth of the reference strain ATCC 25923 and drug-resistant strain MRSA HEMSA 5 at the concentrations of 0.0625–0.125 mM (28.25–56.5 µg/mL). The MICs of remaining compounds were in the range of 0.25 mM to more than 2 mM.

##### Gram-Negative E. aerogenes

Then, MIC values of the compounds were evaluated with Gram-negative *E. aerogenes*. Three following strains of this bacterial species were employed in the studies: (i) the clinical isolate Ea-289 overexpressing the AcrAB-TolC efflux pump and exhibiting a porin-deficient phenotype; (ii) the CM-64 strain which also overexpresses the AcrAB-TolC efflux pump but has no changes in porin content; (iii) the Ea-294 and Ea-298 that are Ea-289 strain derivatives which are devoid of AcrAB and TolC, respectively (Table 4). For most of the compounds, precipitation was observed after the addition of bacterial suspension in MH II broth (**10, 11, 16, 17**). For these molecules, the MICs could not be determined precisely.

As summarized in Table 4, none of the compounds tested exhibited remarkable antibacterial effects against the bacterial strains used in the study (MIC from 0.25 mM to >2 mM). Since imidazolone derivatives **10**, **12** and **13** did not show any significant differences when assayed in efflux pump overexpressing strains (CM-64 and Ea-289) and efflux pump deficient strains (Ea-294 and Ea-308), these compounds were not considered as substrates for the AcrAB-TolC transporter. Furthermore, the absence of porins in Ea-289, Ea-298 and Ea-294 has no significant effect on the activity. Compounds **11** and **17** influenced the strains growth in corresponding manner to that of known EPI, PAβN, increasing MICs at least in 4-fold for Ea-289 and CM-64 in comparison to Ea-298 and Ea-294. Similar trends can be expected for **9** and **16**, but it was impossible to determine it exactly due lack of growth inhibition for Ea-289 and CM-64 caused by both compounds at their highest tested concentration.

#### 2.3.2. Influence on Antibiotic Efficacy

##### Influence on Antibiotic Susceptibility in S. aureus Strains

The lack of a direct antibacterial activity of arylideneimidazolone derivatives tested allowed analyzing their anti-resistance potency in combination with the β-lactam antibiotic, oxacillin. Compounds studied were examined at the concentrations corresponding to 25% of their respective MIC values or at the highest concentrations, at which they did not precipitate. The potential of compounds to become antibiotic adjuvants was evaluated by comparing the drug effectiveness in the presence and absence of compounds tested, thereby, by determining activity gain parameter A (Table 5). Most of the imidazolone derivatives (**6**–**16**) were able to significantly improve the susceptibility of MRSA to oxacillin. At the same time, compounds did not exert any activity against the reference *S. aureus* strain (A < 4). The strongest chemosensitizing effect was demonstrated by compounds **16** and **9**, which, at a concentration of 0.125 mM, reduced the MIC of oxacillin in 16- to 32-fold against MRSA HEMSA 5. Slightly lower adjuvant-like activity was determined for the imidazolone derivatives **7** and **10** (A = 8–16). The efficacy of the remaining compounds was slightly less marked, however, also significant. Although compounds tested improved noticeably the antibacterial activity of oxacillin against MRSA clinical isolate, none of them restored the activity of the antibiotic (MIC < 2 µg/mL) against this highly resistant strain.

##### Influence on Antibiotic Susceptibility in E. aerogenes

Since the compounds were found to be deprived of an intrinsic antibacterial effect in *E. aerogenes* strains, their ability to enhance the activity of several antibiotics was assessed. The following four antibiotics were selected for the assays: chloramphenicol, erythromycin, doxycycline, and norfloxacin. The compounds were tested at the concentration corresponding to ¼ of their MIC. For compounds **9**, **11**, **16** and **17**, for which determination of the exact intrinsic MIC value was not possible (MIC > 2 mM, Table 4), the concentration of 0.5 mM was chosen. As in previous studies [10], PAβN was used as reference molecule in the assays. Results of the assays indicated that none of the tested compounds significantly increased the effectiveness of chloramphenicol, erythromycin, doxycycline and norfloxacin against both AcrAB-TolC-overexpressing strains and AcrAB-TolC-deficient strains (A ≤ 2). The A value obtained for PAβN combined with the aforementioned antibiotics coincided with data found in the literature [31].

#### 2.3.3. Efflux Pump Inhibitory Properties

Taking into account the structural similarity of the explored group (**6**–**17**) to previously found potent efflux pump inhibitors for AcrAB-TolC (**4a**, **4b** and **5**), the representative structures (**9**–**13**, **16** and **17**) were also evaluated for their ability to inhibit the activity of AcrAB-TolC pump in *E. aerogenes* by measuring the efflux of a dye-substrate in the real-time efflux assay (RTE). The RTE assay is a dynamic tool by which the ability of a potential EPI to inhibit substrate transport in a drug efflux pump can be measured as a function of time and energy. Moreover, the test cannot discriminate for a possible outer membrane-destabilizing action of compounds. The dye, 1,2′-dinaphthylamine (1,2′-dNA), a substrate of the AcrAB-TolC efflux pump, is a sensitive to highly lipophilic environment where it is fluorescent, but it is almost non-fluorescent in an aqueous solution [32]. The experiment was performed in the *E. aerogenes* strain overexpressing AcrAB-TolC transporter (Ea-289).

**Figure 5:**
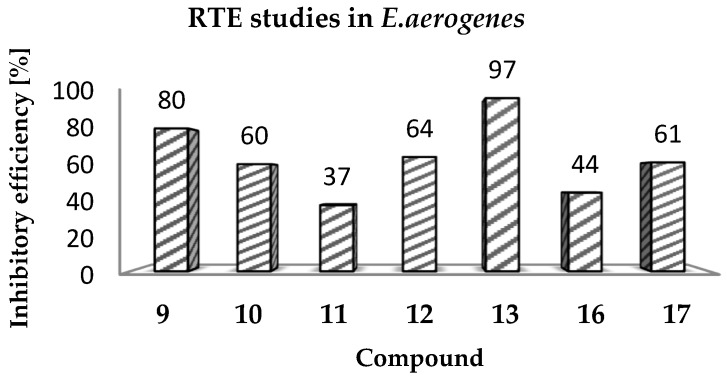
Comparison of the inhibitory activity of imidazolone derivatives towards AcrAB-TolC efflux pump in *E. aerogenes* Ea-289 strain.

Results have demonstrated that majority of the tested compounds caused a decrease in the fluorescence of 1,2′-dNA in the treated bacterial cell. The effect observed was most probably due to molecular interactions between fluorophore and compounds tested that result in increasing fluorescence quenching [33,34]. In order to analyze and quantitatively compare the ability of tested compounds to inhibit the efflux of 1,2′-dNA in the RTE assay, the inhibition efficiency (IE) of each compound was calculated. The IE parameters have indicated that all the tested compounds promoted the accumulation of 1,2′-dNA through the inhibition of its efflux outside bacterial cells overexpressing AcrAB-TolC (Figure 5).

The highest EPI property was detected for the compound **13**, which at the concentration of 100 µM almost completely blocked 1,2′-dNA efflux from the bacteria overexpressing the AcrAB-TolC transporter. Slightly lower effectiveness was found for the compound **9** (80% of efflux inhibition). By contrast, the weakest EPI activity among all compounds tested was determined for the compound **11** which blocked the efflux of the dye in 37% in the Ea-289 strain.

### 2.4. Studies in silico

#### 2.4.1. Docking Studies

In order to estimate potential molecular mechanism of the significant oxacillin adjuvant action of the tested imidazolones in MRSA strains, molecular modeling was applied. The attempts to explain the mechanism were based on the verification if compounds tested had the capacity to interact with the main proteins conferring β-lactam resistance to MRSA strains. For this purpose, all the compounds tested (**6**–**17**) were docked to the crystal structure of PBP2a protein. Moreover, the arylidenemidazolones were subjected to molecular dynamics simulation analysis, which offers an important insight into the molecular behavior of a molecule in its immediate microenvironment.

**Figure 6:**
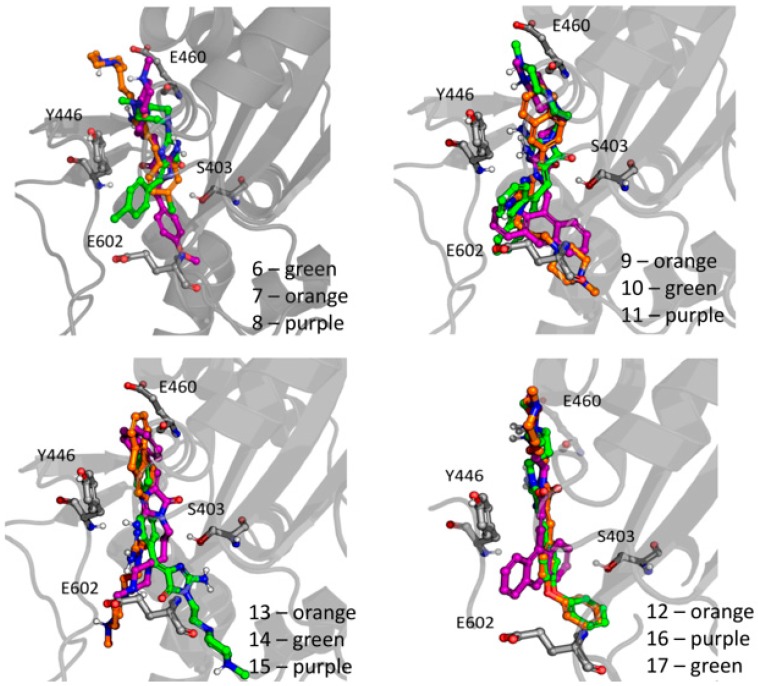
Docking poses of the studied compounds in the active site of PBP2a (grid centered at S403).

**Figure 7:**
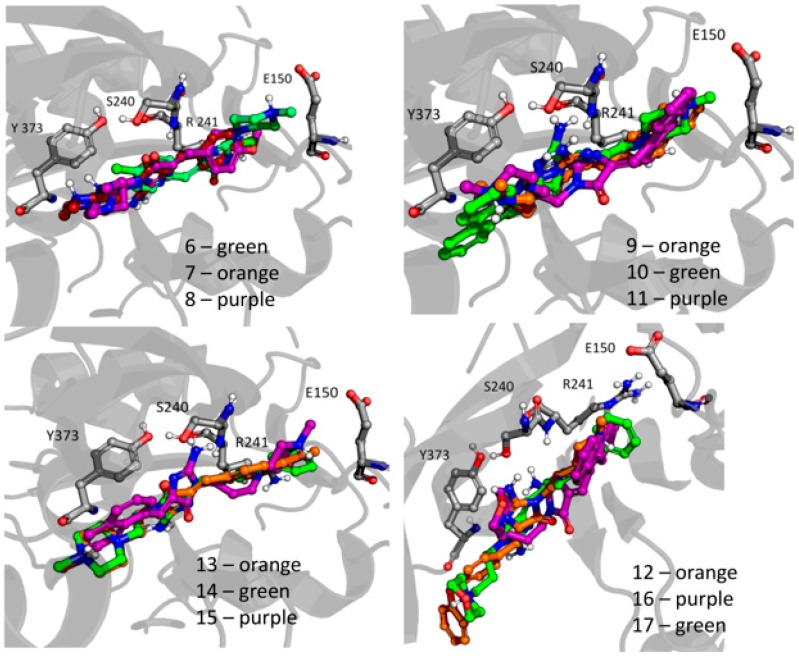
Docking poses of the studied compounds in the allosteric site of PBP2a (grid centered at S240).

The results of docking studies are presented in the form of two groups of docking poses, in respect to the active (Figure 6) and allosteric (Figure 7) sites of PBP2a. Detailed schemes with particular amino acid residues interacting with compounds **6**–**17** are presented in ligand interaction diagrams (Supporting Information). Due to the relatively high number of considered compounds, they are grouped in pictures according to their structural similarity, therefore compounds **6**–**8**, **9**–**11**, **13**–**15** are presented together, and the docking pose of compound **12** is presented together with compounds **16** and **17**. Despite small structural differences occurring within the analyzed groups of compounds, there are quite significant variations in their docking poses (Figure 6).

When compounds **6**–**8** are considered (among which compound **7** displayed the highest activity, reducing MIC of oxacillin by 16-fold in comparison to 8-fold reduction observed for compounds **6** and **8**), the most pronounced difference is connected with the orientation of the phenyl ring, which for compound **7** is located the closest to the serine at position 403 of PBP2a. Moreover, for compound **7** and **8**, a set of hydrogen bonds is observed (charged assisted hydrogen bond of piperazine ring with glutamic acid at position 447 and 460 and hydrogen bond of amine group with serine at position 461), which do not occur for compound **6** (see ligand interaction diagrams in the Supporting Information). Compounds **9**–**11**, which are analyzed together, also displayed relatively high activity in terms of restoring oxacillin activity, with the most active compound **9** (16-fold reduction of MIC of oxacillin). All those compounds are differently oriented in the active site of PBP2a in comparison to compounds **6**–**8**, and all of them form hydrogen bond with oxygen from imidazolone ring; however, different residues from the side of protein are involved in this type of interaction: N464 for compound **9**, T600 for compound **10**, and S403 for compound **11**. Compounds **13**–**15**, for which the docking poses to the active site of PBP2a were analyzed together, displayed slightly lower activity. Compound **14**, reducing the MIC of oxacillin by 8-fold, oriented its piperazine part rather outside of the protein and oxygen from imidazolone ring formed hydrogen bond with E602 and Q521. Compounds **13** and **15** were located in different part of the PBP2a active site than compound **14**, with aromatic rings forming pi–pi interactions with Y446 and H583, respectively, and hydrogen bonds of oxygen in imidazolone moiety with N464 and S462, respectively. The last group of analyzed compounds was the most diversified in terms of activity; there was the most active compound **16** (16–32 fold reduction of MIC of oxacillin), compound **12**, which displayed an 8-fold MIC reduction, and compound **17**, which was not effective in restoring oxacillin activity (2-fold MIC reduction). Compound **16** is differently oriented in comparison to compounds **12** and **17**. It seems that the clue for activity of this compound lies in the position of aromatic rings of anthracene, located closely to S403 residue. The network of hydrogen bonds is similar for all of these compounds, as they all form this type of interaction with S461 and S463.

Figure 7 presents docking results to the allosteric pocket of PBP2a with the compounds grouped in an analogous way as previously.

For compounds **6**–**8**, there are high variations in docking poses, as the pose of compound **6** depicted in green is flipped in comparison to poses of compound **7** and **8**, and the docking pose of the most active compound **7** is only slightly different from less active compound **8**. An analogous situation occurs for compounds **9**–**11**, where molecules are differently docked to the allosteric site of PBP2a, but the flipped orientation of compound **10** (which has similar activity to compound **9** and **11**) is difficult to correlate with observed activity relationships (an analogous situation occurs for compounds **13**–**15**). The last group of analyzed compounds (**12**, **16**, **17**) with activity decreasing in the following way: **16** > **12** > **17** adopted significantly different docking poses. The most active compound **16** displays an extended network of ligand–protein interactions, including those that occur for the anthracene moiety: lysine at position 148, glutamic acid at position 150 and arginine at position 151. For compound **16**, a set of hydrogen bonds that is not observed for other compounds in the analyzed subgroup is present, which take part in the interactions with serine and threonine from positions 240 and 238, respectively.

#### 2.4.2. Molecular Dynamic Studies

**Figure 8:**
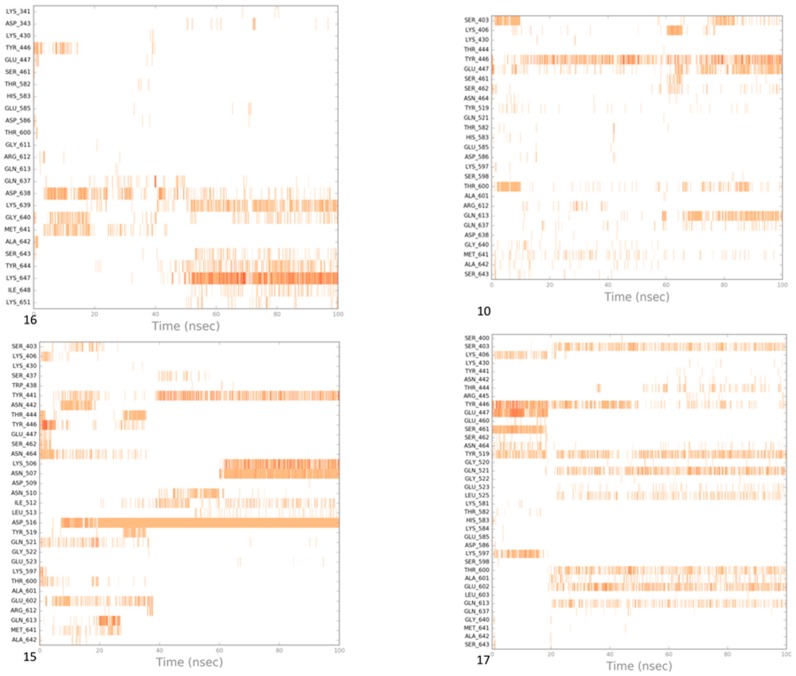
Ligand–protein interaction diagrams obtained for the PBP2a active site during molecular dynamic simulations for compounds **16**, **10**, **15** and **17**.

**Figure 9:**
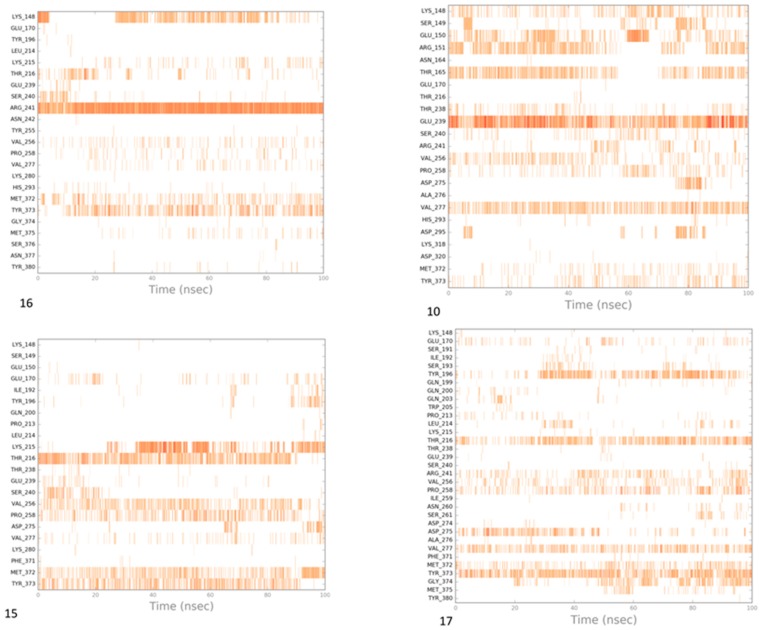
Ligand–protein interaction diagrams obtained for PBP2a allosteric site during molecular dynamic simulations for compounds **16**, **10**, **15** and **17**.

As docking captures the compound orientation only in one moment, molecular dynamic simulation studies were carried out in order to explain more comprehensively the observed activity relationships. Changes in the ligand–protein interactions in time that were observed during simulations are presented in Figure 8 and Figure 9 for dockings to the active and allosteric site of PBP2a, respectively. For simplicity, we have presented the results for only selected compounds; all data is available in the Supporting Information.

When simulations of compounds in the PBP2a active site are analyzed (Figure 8), the most striking observation is the change in orientation of the most active compound (**16**), approximately after 50 ns of simulation, which resulted in the loss of interaction with Q637 and M641 and the formation of interaction with S643, Y644, K647, I648 and K651. On the other hand, a slightly less active compound (**10**) preserved interaction with Y446 (which was lost by compound **16** at the beginning of the simulation), Q637 and M641, although the last two interactions are not very frequent despite being present from time to time during the whole simulation. Similar changes in compound orientation occurred for compound **15** (4-fold reduction of MIC of oxacillin), which was observed after approximately 40 ns of simulation and was connected with the loss of a set of interactions (e.g., T444, Y446, N464, Q521, and E602), the formation of interactions with S437, N510 and I512, and later also with K506, N507, and L513. Additionally, inactive compound **17** moved from its initial docking orientation and it happened the fastest out of all tested compounds (after approximately 20 ns). It led to the loss of interactions e.g., with E447, S461, N464, and K597 and formation of interactions with S403, Q521, L525, T600, E602, and Q613.

On the other hand, when the outcome of molecular dynamic simulations within the allosteric site of PBP2a is analyzed (Figure 9), the most visible observation from the ligand interaction diagrams is the significantly greater perseverance for all of the considered compounds. For the most active compound (**16**), the very strong interaction with R241 occurs for almost 100% of the simulation time, and although interactions with other protein residues are less frequent, they are also present during the whole simulation. The slightly different pose of compound **10** led to a different set of present interactions, the most frequent of which was with E239. Compound **15** interacted more frequently with K215 and T216, although the latter interaction appears after about 20 ns of simulation. For the inactive compound **17**, there is no such interaction that occurs for almost 100% of simulation time, although the compound pose in the allosteric site of PBP2a is also rather preserved during the whole molecular dynamic studies.

The observed dependencies in docking poses and changes in ligand–protein interactions during molecular dynamic simulation studies suggest the allosteric mechanism of action of the studied compounds that is an interaction with PBP2a in the allosteric site, improving binding of oxacillin in the PBP2a active site.

### 2.5. ADMET Studies

Compound **9**, as the most active agent considering both oxacillin adjuvant- and AcrAB-TolC inhibitor properties****, was chosen to be tested on ADME-Tox properties in vitro.

#### 2.5.1. Membrane Permeability

The permeability of compound **9** was estimated by the commercially available Pre-coated PAMPA (Parallel Artificial Membrane Permeability Assay) Plate System Gentest™ according to previously described protocols and formulas [35,36]. The calculated permeability coefficient for **9** (Pe = 0.72 ± 0.27 × 10^−6^ cm/s) was comparable to the Pe value obtained for the low permeable reference norfloxacin and much lower than that for the highly permeable caffeine (Table 6). Thus, the passive movement of compound **9** across the cell membranes was determined as low.

#### 2.5.2. Safety Assays In Vitro

The bacterial and eukaryotic in vitro cell growths were used to get results of potential mutagenicity and hepatotoxicity of compound **9**.

The mutagenicity was evaluated using Ames microplate fluctuation protocol (MPF) performed with *Salmonella typhimurium* TA100 strain, which is able to detect base pair substitutions [37]. Calculation of medium control baseline (MCB) was performed basing on mean number of revertants in standard medium control in addition with one standard deviation. According to the manufacturer’s protocol, the mutagen alert is determined as 2-fold of medium control baseline (2 x MCB), which in this study was calculated as 24 revertants. For the control mutagen nonyl-4-hydroxyquinoline-*N*-oxide (NQNO, 0.5 µM) more than 40 revertants was observed, whereas in the presence of tested compound **9** maximum 11 revertants occurred (Table 7). The obtained data indicates no mutagenicity potential of the tested compound. However, the Binomial B-value = 0.0093, calculated for **9** at the 10 µM concentration, showed possible cytotoxic effect of **9** against *S. typhimurium* TA100 (Table 7).

**Figure 10:**
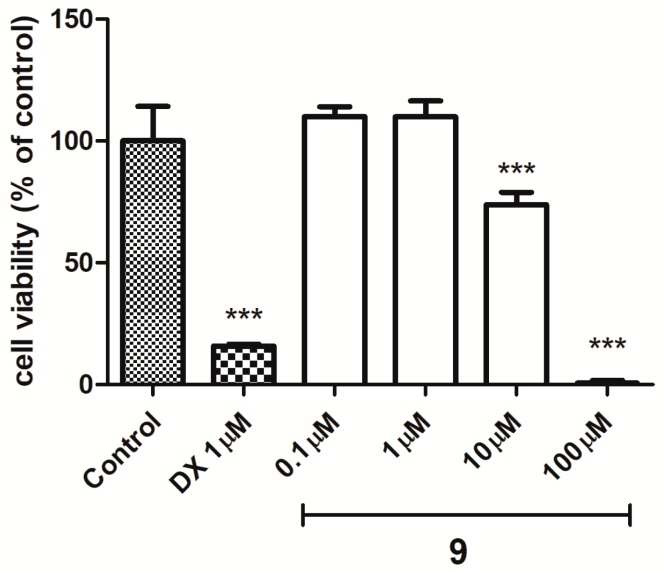
The viability of HepG2 cell line after incubation with compound **9** for 72 h. DX—doxorubicin. Control—1% DMSO in the cell culture media. The statistical significance was evaluated by a one-way ANOVA, followed by Bonferroni’s Comparison Test (*** *p* < 0.001 compared with control).

Determination of potential hepatotoxicity was performed with *hepatoma* HepG2 cells by standard colorimetric MTS procedure. Compound **9** was diluted into the cell culture media in the following concentrations: 0.1, 1, 10 and 100 μM, added to the cells and incubated for 72 h at 37 ºC in the presence of 5% CO_2_. The cytostatic drug doxorubicin (DX) was used at 1 µM, as the reference. The obtained results showed that compound **9** caused statistically significant decrease of the HepG2 cells viability at the concentrations 10 and 100 µM (Figure 10). However, in comparison to the result obtained for the reference DX the hepatotoxicity potential of tested compound is moderate, as its decreased cells’ viability to ~74% of control at 10 µM, whereas DX to ~15% of control at 1 µM (Figure 10).

### 2.6. SAR Discussion

All synthesized compounds (**7**–**17**) belong to the group of 5-arylideneimidazolones with amine moiety at position 2 (**6**) and after Dimroth rearrangement (Scheme 1) also at position 3 (**7**–**17**). A variety of arylidene moieties at position 5 and distinct position and kind of amines give an opportunity to evaluate impact of these fragments for the antibiotic adjuvant properties considered. Most of the tested compounds displayed significant (at least four) reduction of oxacillin MIC in MRSA HEMSA 5 strain (Table 5). In previous tests of 2-amine-5-arylideneimidazolones, only two compounds displayed a significant reduction of oxacillin MIC in the same bacterial strain. However, the decrease of antibiotic MIC was higher (64- to 128-fold) [25]. Additionally, a synergistic effect with oxacillin was not observed in the case of the susceptible strain (ATCC), what indicated an ability to selectively block mechanisms of resistance occurring in the MDR pathogen. The outcome seems to be more profitable for imidazolones with condensed aromatic rings at the position 5 (8–32-fold activity gain for **9**–**11**, **13**, **15** and **16**). The same conclusion was made for previously tested 5-arylideneimidazolones with unsubstituted piperazine at position 2 [25]. Comparing the 2-naphthyl derivative (**9**) and 1-naphthyl (**15**), the first one (**9**) was more potent. There was no difference between the adjuvant potency of biphenyl (**14**) and 4-phenoxyphenyl (**12**) derivatives. In the case of mono-aromatic 5-benzylideneimidazolones, chlorine (**7**) in para position was more profitable than MeO. Compound **16** with an anthracene moiety and the morpholine-terminated fragment at position 3 showed the highest ability to restore oxacillin activity, and also demonstrated more potent ligand–protein interactions in the allosteric site in molecular modelling studies. The similar anthracene derivative of methylpiperazine (**11**) was less active, thus indicating a predominant role for morpholine. Additionally, the 5-anthracylmethylideneimidazolone derivative with a 2-piperazine moiety was active in the same MRSA strain [25]. On this account, this relationship seems to be aryl-dependent. In the case of phenoxybenzylidene derived imidazolones, morpholine derivative (**17**) were less active than the methylpiperazine analogue (**12**). Considering the topology of an amine moiety, the presence of aminoalkyl fragment at position 3 of imidazolone (**7**) seems to be significantly more favourable than the presence of the tertiary amine substituted directly at imidazolone position 2 (**6**).

Compounds **9–13, 16** and **17** were additionally tested for their activity in *E. aerogenes* strains. Although 5-arylideneimidazolone derivatives did not display antibiotic adjuvant activity in assays on their synergistic effects with chloramphenicol, erythromycin, doxycycline and norfloxacin, these compounds were able to block AcrAB-TolC efflux activity in RTE assay. The highest inhibitory efficiency (97%) demonstrated the fluorene derivative (**13**), while imidazolones with three fused aromatic rings, antracene (**11**, **16**) and phenanthrene (**10**), showed moderate ability to block efflux pumps (37–60%). These results confirmed the previous findings about EPI’s action in AcrAB-TolC pump in different *E. coli* strains in the group of imidazolones [15]. In turn, the presence of condensed aromatic rings at position 5 was beneficial either for MRSA or for *E. aerogenes* MDR reversal action. In previous studies, the 2-piperazine derivative of 5-(2-naphthyl)imidazolone was the most potent (128-fold reduction of oxacillin MIC) in the MRSA HEMSA 5 strain [25]. Although, taking into account results of both, the adjuvant potency in MRSA and efflux pump inhibitory action in *E. aerogenes*, the 2-naphthyl and methylpiperazine derivative (**9**) was found as the most potent “dual agent”. Hence, the compound was selected as representative structure for primary ADMET assays in vitro. The obtained results allowed us to classify this compound as lowly permeable (Table 6), non-mutagenic but cytotoxic for prokaryotic cells (Table 7), and displaying slight hepatotoxic effects (Figure 10). This rather moderate “drugability” found did not exclude a better drug-like property for other active members of the investigated imidazolones, but indicated a strong need of further studies within this interesting chemical group. Thus, new chemical modifications as well as an extension of biological- and “drugability” screening will be intentional for this new family of 3-substituted 5-arylideneimidazolones in the near future.

## 3. Experimental

### 3.1. Chemistry

Reagents were purchased from Alfa Aesar (Karlsruhe, Germany) or Sigma Aldrich (Darmstadt, Germany). Reaction progress was verified using thin layer chromatography (TLC), which was carried out on 0.2 mm Merck silica gel 60 F254 plates. Spots were visualized by UV light. Melting points (m.p.) were determined using the MEL-TEMP II apparatus (LD Inc., Long Beach, CA, USA) and are uncorrected. The ^1^H-NMR and ^13^C-NMR spectra were obtained on a Mercury-VX 300 Mz spectrometer (Varian, Palo Alto, CA, USA) in DMSO-d_6_. Chemical shifts in ^1^H-NMR spectra were reported in parts per million (ppm) on the δ scale using the solvent signal as an internal standard. Data are reported as follows: Chemical shift, multiplicity (s, singlet; br.s, broad singlet; d, doublet; d def.-doublet deformated; t, triplet; t def.-triplet deformated; qui, quintet; m, multiplet; taut.-tautomerism), coupling constant J in Hertz (Hz), number of protons, proton’s position (Ar- aromatic moiety at position 5, Ph—phenyl, Pip—piperazine, Mor—morpholine). Mass spectra were recorded on a UPLC-MS/MS system consisted of a Waters ACQUITY^®^UPLC^®^ (Waters Corporation, Milford, MA, USA) coupled to a Waters TQD mass spectrometer (electrospray ionization mode ESI-tandem quadrupole). Chromatographic separations were carried out using the Acquity UPLC BEH (bridged ethyl hybrid) C18 column; 2.1 × 100 mm, and 1.7 µm particle size, equipped with Acquity UPLC BEH C18 VanGuard precolumn (Waters Corporation, Milford, MA, USA); 2.1 × 5 mm, and 1.7 µm particle size. The column was maintained at 40 °C and eluted under gradient conditions from 95% to 0% of eluent A over 10 min, at a flow rate of 0.3 mL·min^−1^. Eluent A: water/formic acid (0.1%, *v/v*); eluent B: acetonitrile/formic acid (0.1%, *v/v*). Chromatograms were made using Waters eλ PDA detector. Spectra were analyzed in the 200–700 nm range with 1.2 nm resolution and sampling rate 20 points/s. MS detection settings of Waters TQD mass spectrometer were as follows: source temperature 150 °C, desolvation temperature 350 °C desolvation gas flow rate 600 L·h^−1^, cone gas flow 100 L·h^−1^, capillary potential 3.00 kV, cone potential 40 V. Nitrogen was used for both nebulizing and drying gas. The data were obtained in a scan mode ranging from 50 to 1000 *m/z* in time 0.5 s intervals. Data acquisition software was MassLynx V 4.1 (Waters Corporation, Milford, MA, USA). The UPLC/MS purity of all the final compounds was confirmed to be 95% or higher. Retention times (t_R_) are given in minutes. The UPLC/MS purity of all final compounds was determined (%). Synthesis of compounds **6**, **18**–**20**, **22**, **25–30**, **32, 35** and **36** were described earlier [15,25,26,38,39,40,41,42].

#### 3.1.1. General Procedure to Obtain 5-Arylidenethiohydantoin (**21**, **23** and **24**)

Thiohydantoin (2.90–5.80 g, 25–50 mmol), acetic acid (25–50 ml), sodium acetate (8.33–16.67 g, 100–200 mmol) with appropriate arylidene aldehyde (25–50 mmol) in flat-bottom flask were heated in boiling point for 4–6 h and then mixed for 20 h. Reaction was controlled by TLC-chloroform/ethyl acetate: 1/1. If necessary, purification was performed using crystallization from acetone or acetic acid.

*(Z)-5-(phenanthren-9-ylmethylene)-2-thioxoimidazolidin-4-one* (**21**) Phenanthrene-9-carbaldehyde (30.5 mmol, 6.29 g) and thiohydantoin (30.5 mmol, 3.54 g) were used. Yellow solid. Yield 99%; mp 283–285 °C. C_18_H_12_N_2_OS MW 304.37. LC/MS ±: purity 96.05% t_R_ = 6.42, (ESI) *m/z* [M+H] 305.04. ^1^H-NMR δ [ppm]: 8.94–8.63 (m, 3H, N1-H, Ar-4,5-H), 8.30 (m, 1H, Ar-1-H), 8.03–7.87 (m, 1H, Ar-8-H), 7.80–7.61 (m, 4H, Ar-2,3,6,7-H), 7.12 (s, 1H, Ar-10-H), 6.76 (s, 1H, C = CH).

*(Z)-5-(4-phenoxybenzylidene)-2-thioxoimidazolidin-4-one* (**23**) 4-Phenoxybenzaldehyde (50 mmol, 9.91 g) and thiohydantoin (50 mmol, 5.81 g) were used. Yellow solid. Yield 81%; mp 233–235 °C. C_16_H_12_N_2_O_2_S MW 296.34. LC/MS ±: purity 99.11% t_R_ = 6.52, (ESI) *m/z* [M+H]^+^ 297.07. ^1^H-NMR δ [ppm]: 12.00 (br.s, 1H, N3-H), 7.80–7.77 (d def., 2H, Ph-2,6-H), 7.41–7.39 (d def., 2H, Ph-3,5-H), 7.20–6.97 (m, 5H, Ph’-2,3,4,5,6-H), 6.42 (s, 1H, C = CH), 3.52 (br. s, 1H, taut. N1-H<->SH).

*(Z)-5-((9H-fluoren-2-yl)methylene)-2-thioxoimidazolidin-4-one* (**24**) 9H-Fluorene-2-carbaldehyde (25 mmol, 4.86 g) and thiohydantoin (25 mmol, 2.90 g) were used. Yellow solid. Yield 86%; mp 276–278 °C. C_17_H_12_N_2_OS MW 292.35. LC/MS ±: purity 94.88% t_R_ = 6.45, (ESI) *m/z* [M+H]^+^ 293.01. ^1^H-NMR δ [ppm]: 12.38 (br.s, 1H, N3-H), 12.38 (br.s, 1H, N3-H), 12.22 (br.s, 1H, N1-H), 8.04 (s, 1H, Ar-1-H), 7.96–7.92 (m, 2H, Ar-4,5-H), 7.71 (d, J = 7.95 Hz, 1H, Ar-8-H), 7.60 (d, J = 6.92 Hz, 1H, Ar-3-H), 7.43–7.32 (m, 2H, Ar-6,7-H), 6.57 (s, 1H, CH = C), 3.96 (s, 2H, Ar-9-CH_2_).

#### 3.1.2. General Procedure to Obtain 2-Methylthio-5-Arylidenethiohydantoins (**31**, **33**, **34** and **37**)

Sodium (0.48–0.87 g, 21.00–37.77 mmol) was put into ethanol (21.00–37.77 mL). Sodium ethoxide mixed with appropriate 5–arylidenethiohydantion (21.00–37.77 mmol) in flat–bottom flask for 3 min. Then, iodomethane (2.98–5.36 g, 21.00–37.77 mmol) was added and whole were mixed for 5–24 h. Reaction was controlled by TLC-chloroform/ethyl acetate: 1/1. Purification was performed using crystallization from acetone wherever necessary.

*(Z)-2-(methylthio)-4-(phenanthren-9-ylmethylene)-1H-imidazol-5(4H)-one* (**31**) (Z)-5-(Phenanthren-9-ylmethylene)-2-thioxoimidazolidin-4-one (**21**) (26.00 mmol, 7.91 g) with iodomethane (26.00 mmol, 3.69 g) was used. Yellow solid. Yield 99%; mp 271–274 °C. C_19_H_14_N_2_OS MW 318.39. LC/MS±: purity 97.70% t_R_ = 6.77, (ESI) *m/z* [M+H]^+^ 319.06. ^1^H-NMR δ [ppm]: 9.24–9.10 (m, 1H, N3-H), 8.90–8.81 (m, 2H, Ar-4,5-H), 8.36–8.29 (m, 1H, Ar-8-H), 8.02–7.99 (m, 1H, Ar-1-H), 7.67–7.40 (m, 5H, Ar-2,3,6,7,10-H), 6.87 (m, 1H, C = CH), 2.71 (s, 3H, S-CH_3_).

*(Z)-4-(4-phenoxybenzylidene)-2-(methylthio)-1H-imidazol-5(4H)-one* (**33**) (Z)-5-(4-Phenoxybenzylidene)-2-thioxoimidazolidin-4-one (**23**) (37.00 mmol, 10.96 g) with iodomethane (37.00 mmol, 5.25 g) was used. Yellow solid. Yield 92%; mp 177–179 °C. C_17_H_14_N_2_O_2_S MW 310.37. LC/MS±: purity 96.15% t_R_ = 7.39, (ESI) *m/z* [M+H]^+^ 311.09. ^1^H-NMR δ [ppm]: 11.80 (br.s, 1H, N3-H), 8.29–8.18 (d def., 2H, N3-H, Ph-2,6-H), 7.44–7.38 (t def., 2H, Ph-3,5-H), 7.21–7.13 (m, 1H, Ph’-4-H), 7.09–6.99 (m, 4H, Ph’-2,3,5,6-H), 6.71 (s, 1H, C = CH), 2.62 (s, 3H, S-CH_3_).

*(Z)-5-((9H-fluoren-2-yl)methylene)-2-(methylthio)-3H-imidazol-4(5H)-one* (**34**) (Z)-5-((9H-Fluoren-2-yl)methylene)-2-thioxoimidazolidin-4-one (**24**) (21.00 mmol, 6.14 g) with iodomethane (21.00 mmol, 2.98 g) was used. Yellow solid. Yield 90%; mp 240–242 °C. C_18_H_14_N_2_OS MW 306.38. LC/MS±: purity 91.26% t_R_ = 7.58, (ESI) *m/z* [M+H]^+^ 307.10. ^1^H-NMR δ [ppm]: 8.39 (s, 1H, Ar-1-H), 8.27–8.18 (d def., 1H, Ar-8-H), 7.99–7.87 (m, 2H, Ar-4,5-H), 7.64–7.54 (d def., 1H, Ar-3-H), 7.45–7.20 (m, 3H, Ar-6,7-H), 6.80 ( s, 1H, CH = C), 3.96 (s, 2H, Ar-9-CH_2_), 2.70 (s, 3H, SCH_3_).

*(Z)-5-(3-phenoxybenzylidene)-2-(methylthio)-3H-imidazol-4(5H)-one* (**37**) (Z)-5-(3-Phenoxybenzylidene)-2-thioxoimidazolidin-4-one (**27**) (37.77 mmol, 11.19 g) with iodomethane (37.77 mmol, 5.36 g) was used. Yellow solid. Yield 95%; mp 214–216 °C. C_17_H_14_N_2_O_2_S MW 310.37. LC/MS±: purity 95.24% t_R_ = 7.32, (ESI) *m/z* [M+H]^+^ 311.02. ^1^H-NMR δ [ppm]: 11.80 (s, 1H, N3-H), 8.06 (s, 1H, Ph-2-H), 7.64–7.61 (d def., 1H, Ph-6-H), 7.43–7.38 (m, 3H, Ph-5-H, Ph’-3,5-H), 7.19–7.05 (m, 4H, Ph-4-H, Ph’-2,4,6-H), 6.67 (s, 1H, C = CH), 2.35 (s, 3H, S-CH_3_).

#### 3.1.3. General Procedure for Synthesis of Final Products (**7**–**17**)

(Z)-2-(methylthio)-5-arylidene-3*H*-imidazol-4(5*H*)-one (2–10 mmol) with appropriate amine (3–15 mmol) derivative in 50 mL flat-bottom flask were heated in oil bath with controlled temperature (120–130 °C) for 15 min. Then, ethanol (15-30 ml) was added and mixture was heated for 5–7 h and mixed for the next 20 h. Then, compounds were converted into hydrochloride forms by gaseous hydrochloride acid obtained from reaction between sodium chloride and sulphuric acid. Purification was performed using crystallization from ethanol wherever necessary.

*(Z)-5-(4-chlorobenzylidene)-2-amino-3-(3-(4-methylpiperazin-1-yl)propyl)-3H-imidazol-4(5H)-one hydrochloride* (**7**); (Z)-5-(4-Chlorobenzylidene)-2-(methylthio)-3*H*-imidazol-4(5*H*)-one (**28**) (10 mmol, 2.53 g) and 3-(4-methylpiperazin-1-yl)propan-1-amine (15 mmol, 2.36 g) were used. White solid. Yield 7.5%; mp 294 °C. C_18_H_24_ClN_5_Ox3HClxH_2_O MW 486.25. LC/MS ±: purity 100.00% t_R_ = 3.34, (ESI) *m/z* [M+H]^+^ 362.26. ^1^H-NMR δ [ppm]: 11.82 (br. s, 1H, NH^+^), 9.22 (br. s, 2H, NH_2_-taut.: N1-H, C2 = NH), 7.84 (d, J = 8.46 Hz, 2H, Ar-2,6-H), 7.49 (d, J = 8.72 Hz, 2H, Ar-3,5-H), 6.77 (s, 1H, CH = C), 3.78 (t, J = 6.41 Hz, 2H, N3-CH_2_), 3.48 (br. s, 8H, Pip), 3.19 (br. s, 2H, Pip-CH_2_), 2.80 (br. s, 3H, CH_3_), 2.02 (br. s, 2H, N-CH_2_-CH_2_).

*(Z)-5-(4-Methoxybenzylidene)-2-amino-3-(3-(4-methylpiperazin-1-yl)propyl)-3H-imidazol-4(5H)-one hydrochloride* (**8**); (Z)-5-(4-Methoxybenzylidene)-2-(methylthio)-3H-imidazol-4(5H)-one (**29**) (10 mmol, 2.46 g) and 3-(4-methylpiperazin-1-yl)propan-1-amine (15 mmol, 2.36 g) were used. Yellow solid. Yield 15%; mp 262 °C. C_19_H_27_N_5_O_2_x3HCl·0.5H_2_O MW 475.93. LC/MS±: purity 100.00% t_R_ = 2.58, (ESI) *m/z* [M+H]^+^ 358.34. ^1^H-NMR δ [ppm]: 11.8 (br. s, 1H, NH^+^), 9.5 (br. s, 2H, NH_2_-taut.: N1-H, C2 = NH), 7.76 (d, J = 8.72 Hz, 2H, Ar-2,6-H), 7.01 (d, J = 8.72 Hz, 2H, Ar-3,5-H), 6.84 (s, 1H, CH = C), 3.81 (s, 3H, O-CH_3_), 3.70–3.20 (m, 12H, Pip, Pip-CH_2_, N3-CH_2_), 2.79 (s, 3H, CH_3_), 2.00 (br. s, 2H, N-CH_2_-CH_2_).

*(Z)-2-Amino-3-(3-(4-methylpiperazin-1-yl)propyl)-5-(naphthalen-2-ylmethylene)-3H-imidazol-4(5H)-one hydrochloride* (**9**) (Z)-2-(methylthio)-4-(naphthalen-2-ylmethylene)-1H-imidazol-5(4H)-one (**30**) (5 mmol, 1.34 g) and 3-(4-methylpiperazin-1-yl)propan-1-amine (10 mmol, 1.57 g) were used. Yellow solid. Yield 74%; mp 215-217 °C. C_22_H_28_ClN_5_O MW 413.94. LC/MS±: purity 100.00% t_R_ = 3.34, (ESI) *m/z* [M+H]^+^ 378.28. ^1^H-NMR δ [ppm]: 8.39–8.34 (m, 2H, Ar-5,8-H), 7.85–7.78 (m, 5H, NH_2_, Ar-1,3,4-H), 7.47–7.45 (m, 2H, Ar-6,7-H), 6.50 (s, 1H, C = CH), 3.54 (t, J = 6.40Hz, N3-CH_2_), 2.45–2.20 (m, 10H, Pip, Pip-CH_2_), 2.09 (s, 3H, CH_3_), 1.68 (qui, J = 6.40Hz, 2H, N-CH_2_-CH_2_).

*(Z)-2-Amino-3-(3-(4-methylpiperazin-1-yl)propyl)-5-(phenanthren-9-ylmethylene)-3H-imidazol-4(5H)-one hydrochloride* (**10**) (Z)-2-(Methylthio)-5-(phenanthren-9-ylmethylene)-3H-imidazol-4(5H)-one (**31**) (2 mmol, 0.62 g) and 3-(4-methylpiperazin-1-yl)propan-1-amine (3 mmol, 0.47 g) were used. Yellow solid. Yield 17%; mp 266–268 °C. C_26_H_30_ClN_5_O MW 464.00. LC/MS±: purity 99.00% t_R_ = 4.03, (ESI) *m/z* [M+H]^+^ 428.19. ^1^H-NMR δ [ppm]: 8.89–8.86 (m, 2H, Ar-1,10-H), 8.80–8.78 (m, 2H, Ar-4,7-H), 8.24–8.21 (m, 1H, Ar-6-H), 7.89 (s, 2H, NH_2_), 7.73–7.69 (m, 2H, Ar-2,5-H), 7.68–7.64 (m, 2H, Ar-3,8-H), 6.93 (s, 1H, C = CH), 3.60–3.20 (m, 2H, N3-CH_2_), 2.45–2.15 (m, 10H, Pip, Pip-CH_2_), 2.09 (s, 3H, CH_3_), 1.75 (m, 2H, N-CH_2_-CH_2_).

*(Z)-2-Amino-5-(anthracen-10-ylmethylene)-3-(3-(4-methylpiperazin-1-yl)propyl)-3H-imidazol-4(5H)-one hydrochloride* (**11**) (Z)-5-(Anthracen-10-ylmethylene)-2-(methylthio)-3H-imidazol-4(5H)-one (**32**) (4 mmol, 1.71 g) and 3-(4-methylpiperazin-1-yl)propan-1-amine (7.5 mmol, 1.18 g) were used. Yellow solid. Yield 61%; mp 246-248 °C. C_26_H_30_ClN_5_O MW 464.00. LC/MS±: purity 100.00% t_R_ = 3.89, (ESI) *m/z* [M+H]^+^ 428.26. ^1^H-NMR δ [ppm]: 8.61 (br. s, 1H, Ar-5-H), 8.11-8.01 (m, 6H, Ar-1,4,6,9-H, NH_2_), 7.53–7.52 (m, 4H, Ar-2,3,7,8-H), 7.00 (s, 1H, C = CH), 3.40–3.15 (m, 2H, N3-CH_2_), 2.56–2.06 (m, 13H, Pip, Pip-CH_2,_ CH_3_), 1.58–1.52 (m, 2H, N-CH_2_-CH_2_).

*(Z)-5-(4-Phenoxybenzylidene)-2-amino-3-(3-(4-methylpiperazin-1-yl)propyl)-3H-imidazol-4(5H)-one* (**12**) (Z)-5-(4-Phenoxybenzylidene)-2-(methylthio)-1H-imidazol-5(4H)-one (**33**) (4 mmol, 1.19 g) and 3-(4-methylpiperazin-1-yl)propan-1-amine (5 mmol, 0.78 g) were used. Yellow solid. Yield 68%; mp 220–230 °C. C_24_H_30_ClN_5_O_2_ MW 455.98. LC/MS±: purity 100.00% t_R_ = 4.1, (ESI) *m/z* [M+H]^+^ 420.28. ^1^H-NMR δ [ppm]: 7.62–7.57 (m, 3H, NH_2,_ Ph’-4-H), 7.46–7.36 (m, 4H, Ph-2,6-H, Ph’-3,5-H), 7.15–6.95 (m, 4H, Ph-3,5-H, Ph’-2,6-H), 6.72 (s, 1H, C = CH), 3.77 (br. s, 2H, N3-CH_2_), 3.70–3.14 (m, 10H, Pip, Pip-CH_2_), 2.79 (s, 3H, CH_3_), 1.99 (br. s, 2H, N-CH_2_-CH_2_). ^13^C-NMR (DMSO-d_6_, ppm): δ 157.23, 156.98, 130.56, 124.01, 118.93, 40.77, 40.49, 40.20, 39.92, 39.64, 39.36, 39.08.

*(Z)-5-((9H-Fluoren-2-yl)methylene)-2-amino-3-(2-(4-methylpiperazin-1-yl)ethyl)-3H-imidazol-4(5H)-one hydrochloride* (**13**) (Z)-5-((9H-Fluoren-2-yl)methylene)-2-(methylthio)-3H-imidazol-4(5H)-one (**34**) (3.5 mmol, 1.09 g) and 3-(4-methylpiperazin-1-yl)propan-1-amine (5 mmol, 0.78 g) were used. Orange solid. Yield 69.27%; mp 265–267 °C. C_25_H_30_ClN_5_O MW 451.99. LC/MS±: purity 99% t_R_ = 3.92, (ESI) *m/z* [M+H]^+^. ^1^H-NMR δ [ppm]: 12.00 (br. s, 1H, NH^+^), 9.35 (br. s, 1H, NH_2_-taut: N1-H), 8.32–7.70 (m, 4H, NH_2_*-taut*: C2 = NH, Ar-1,4,5-H), 7.67–7.55 (m, 2H, Ar-3,8-H), 7.49–7.24 (m, 2H, Ar-6,7-H), 6.82 (br. s, 1H, CH = C), 4.08 (s, 2H, Ar-9-CH_2_), 3.90–3.01 (m, 12H, N3-CH_2_, Pip, Pip-CH_2_), 2.90 (s, 3H, CH_3_), 2.06 (m, 2H, N-CH_2_-CH_2_). 13C-NMR (DMSO-d_6_, ppm): δ 144.20, 143.96, 127.42, 126.88, 125.74, 125.51, 121.18, 121.00, 40.94, 40.89, 40.52, 40.24, 39.68, 39.39, 39.24, 38.95.

*(Z)-2-Amino-5-(biphen-4-ylmethylene)-3-(2-(4-methylpiperazin-1-yl)ethyl)-3H-imidazol-4(5H)-one hydrochloride* (**14**) (Z)-5-(Biphen-4-ylmethylene)-2-(methylthio)-3H-imidazol-4(5H)-one (**35**) (2.5 mmol, 0.72 g) and 3-(4-methylpiperazin-1-yl)propan-1-amine (4 mmol, 0.63 g) were used. Yellow solid. Yield 49%; mp 258–260 °C. C_24_H_30_ClN_5_O MW 439.98. LC/MS±: purity 96.39% t_R_ = 3.78, (ESI) *m/z* [M+H]^+^. ^1^H-NMR δ [ppm]: 12.00 (br. s, 1H, NH^+^), 8.24–8.05 (t def., 1H, Ph’-4-H), 7.80 (br. s, 2H, NH_2_), 7.79–7.61 (m, 4H, Ph’-3,5-H, Ph-2,6-H), 7.52–7.30 (m, 4H, Ph’-2,6-H, Ph-3,5-H), 6.83 (br. s, 1H, CH = C), 4.26–2.97 (m, 12H, Pip, Pip-CH_2_, N3-CH_2_), 2.80 (s, 3H, CH_3_), 2.04 (br. s, 2H, N-CH_2_-CH_2_). ^13^C-NMR (DMSO-d_6_, ppm): δ 129.51, 127.11, 40.77, 40.49, 40.21, 39.92, 39.64, 39.36, 39.08.

*(Z)-2-Amino-3-(3-(4-methylpiperazin-1-yl)propyl)-5-(naphthalen-1-ylmethylene)-3H-imidazol-4(5H)-one hydrochloride* (**15**) (Z)-2-(Methylthio)-5-(naphthalen-1-ylmethylene)-3H-imidazol-4(5H)-one (**36**) (5 mmol, 1.34 g) and 3-(4-methylpiperazin-1-yl)propan-1-amine (7 mmol, 1.09 g) were used. Yellow solid. Yield 24%; mp 187–191 °C. C_22_H_28_ClN_5_O MW 413.94. LC/MS±: purity 99.07% t_R_ = 3.21, (ESI) *m/z* [M+H]^+^ 378.21. ^1^H-NMR δ [ppm]: 8.83 (br. s, 1H, NH+), 8.19 (br. s, 2H, taut NH_2_), 7.96–7.88 (d def., 1H, Ar-8-H), 7.85–7.75 (d def., 2H, Ar-4,5-H), 7.61–7.44 (m, 4H, Ar-2,3,6,7-H), 6.98 (br. s, 1H, CH = C), 3.32 (br. s, 2H, N3-CH_2_), 2.47–2.17 (m, 10H, Pip, Pip-CH_2_), 2.12 (s, 3H, CH_3_), 1.70 (br. s, 2H, N-CH_2_-CH_2_). ^13^C-NMR (DMSO-d_6_, ppm): δ 133.79, 131.93, 131.62, 129.16, 128.53, 127.90, 126.89, 126.19, 126.05, 123.27, 55.09, 53.02, 46.12, 40.81, 40.52, 40.24, 39.96, 39.68, 39.20, 39.11.

*(Z)-2-Amino-5-(anthracen-10-ylmethylene)-3-(3-morpholinopropyl)-3H-imidazol-4(5H)-one hydrochloride* (**16**) (Z)-5-(Anthracen-10-ylmethylene)-2-(methylthio)-3H-imidazol-4(5H)-one (**33**) (4 mmol, 1.22 g) and 3-morpholinopropan-1-amine (5 mmol, 0.72 g) were used. Orange solid. Yield 70%; mp 244–246 °C. C_25_H_27_ClN_4_O_2_ MW 450.96. LC/MS±: purity 98.71% t_R_ = 4.19, (ESI) *m/z* [M+H]^+^ 415.17. ^1^H-NMR δ [ppm]: 11.25 (br. s, 1H, NH^+^), 10.21 (br. s, 1H, NH_2_-taut: N1-H), 9.39 (br. s, 1H, NH_2_-taut: C2 = NH), 8.72 (s, 1H, Ar-9-H), 8.25–7.95 (m, 4H, Ar-1,4,5,8-H), 7.68–7.43 (m, 5H, Ar-2,3,6,7-H, CH = C), 4.16–2.69 (m, 10H, Mor, N3-CH_2_), 2.12 (br.s, 2H, Mor-CH_2_), 1.80 (s, 2H, N-CH_2_-CH_2_). ^13^C-NMR (DMSO-d_6_, ppm): δ 131.49, 129.42, 129.28, 126.77, 125.96, 66.60, 55.99, 53.71, 40.79, 40.50, 40.22, 39.94, 39.66, 39.37, 39.09.

*(Z)-5-(3-phenoxybenzylidene)-2-amino-3-(3-morpholinopropyl)-3H-imidazol-4(5H)-one hydrochloride* (**17**) (Z)-5-(3-Phenoxybenzylidene)-2-(methylthio)-3H-imidazol-4(5H)-one (**37**) (5 mmol, 1.55 g) and 3-morpholinopropan-1-amine (7 mmol, 1.01 g) were used. Yellow solid. Yield 41%; mp 227–230 °C. C_23_H_27_ClN_4_O_3_ MW 442.94. LC/MS±: purity 96.49% t_R_ = 4.37, (ESI) *m/z* [M+H]^+^ 407.19. ^1^H-NMR δ [ppm]: 11.35 (br. s, 1H, NH^+^), 9.45 (br. s, 1H, NH_2_-taut: N1-H), 7.82-7.27 (m, 6H, NH_2_-taut: C2 = NH, Ph-5,6-H, Ph’-3,4,5-H), 7.26–6.85 (m, 4H, Ph-2,4-H, Ph’-2,6-H), 6.74 (br. s, 1H, CH = C), 4.03 (br. s, 4H, Mor-OCH_2_), 3.66–2.65 (m, 8H, N3-CH_2_, Mor-NCH_2,_ Mor-CH_2_), 2.16–1.88 (t def., 2H, N-CH_2_-CH_2_). ^13^C-NMR (DMSO-d_6_, ppm): δ 157.67, 157.41, 130.55, 124.13, 118.97, 63.54, 51.44, 40.81, 40.53, 40.25, 39.97, 39.68, 39.40, 39.12.

### 3.2. Crystallographic Studies

Crystals suitable for an X-ray analysis were obtained by slow evaporation of the solvent at room temperature from acetonitryl solution. Diffraction data for single crystal were collected at 293 K using Oxford Diffraction SuperNova four circle diffractometer, equipped with the Cu (1.54184 Å) Kα radiation source and graphite monochromator. The structure was solved by direct methods using SIR-97 [43]. All non-hydrogen atoms were refined anisotropically using weighted full-matrix least-squares on F^2^. The hydrogen atoms bonded to carbons were included in the structure at idealized positions and were refined using a riding model with U_iso_(H) fixed at 1.2 U_eq_ of C and 1.5 U_eq_ for methyl groups. Hydrogen atoms attached to nitrogen atom were found from the difference Fourier map and refined without any restraints. Refinement and further calculations were carried out using SHELXL [44]. For molecular graphics ORTEP [45] and MERCURY [46] programs were used.

Crystallographic data: C_18_H_24_ClN_5_O, M_r_ = 361.87, 0.36 × 0.48 × 0.54 mm^3^, orthorhombic, space group Pccn, a = 9.381(5) Å, b = 31.114(5) Å, c = 13.119(5) Å, V = 3829(3) Å^3^, Z = 8, T = 293(2)K, 50,215 reflections collected, 3377 unique reflections (R_int_ = 0.0364), R1 = 0.0410, wR2 = 0.1110 [I > 2σ(I)], R1 = 0.0499, wR2 = 0.1197 (all data).

The supplementary for these crystallographic data can be obtained free of charge from the Cambridge Crystallographic Data Centre, deposited as CCDC 1,886,697.

### 3.3. Microbiological Assay

*S. aureus* and *E. aerogenes* bacteria were maintained and grown on Columbia agar (bioMérieux, Marcy-l’Étoile, France) or Trypticase Soy Agar II (TSA II; Becton Dickinson, Franklin Lakes, NJ, USA) supplemented with 5% sheep blood, respectively. Cation-adjusted Mueller-Hinton (MH II) broth used in the microbiological assays was obtained from bioMérieux. Oxacillin, norfloxacin, chloramphenicol, doxycycline, PAβN, and INT were purchased from Sigma Aldrich (St. Louis, MI, USA), whereas erythromycin lactobionate was purchased from Amdipharm (United Kingdom). The compounds tested were dissolved in DMSO (Merc, Stuttgart, Germany) and the solutions obtained were stored at −20 °C until used. Furthermore, the following chemical compounds were used to perform RTE assay: K_2_HPO_4_ (Sigma Aldrich) and MgCl_2_ (Sigma Aldrich) to prepare potassium phosphate buffer (PPB), CCCP (Sigma Aldrich), 1,2′-dNA (TCI-Europe, Zwijndrecht, Belgium), glucose (Sigma Aldrich).

#### 3.3.1. Susceptibility Testing

Susceptibility testing was performed by the 2-fold standard microdilution method in MH II broth following the Clinical and Laboratory Standards Institute (CLSI) and the Comité de l’Antibiogramme de la Société Française de Microbiologie (CA-SFM) recommendations [47,48]. MIC values were detected in an Infinite M200 pro Tecan microplate reader (Tecan^®^ France, SA-Lyon, France) after overnight incubation at 37 °C. Experiments were carried out in triplicate and the resulting medians were presented.

First, minimum inhibitory concentration (MIC) values of a series of imidazolidine derivatives were examined against *S. aureus* and *E. aerogenes* strains. Then, MICs of selected antibiotics were determined in the absence and in the presence of compounds in order to measure the adjuvant-like effect of these structures. In the latter case, compounds tested were evaluated for the ability to enhance antibacterial activity of β-lactam antibiotic oxacillin in methicillin-resistant and methicillin-susceptible *S. aureus* (MRSA and MSSA) strains as well as chloramphenicol, erythromycin, doxycycline, and norfloxacin in AcrAB-TolC-overexpressing and AcrAB-TolC-deficient mutants of *E. aerogenes*. The concentrations of imidazolidine derivatives used in the MIC reduction assay were not greater than 25% of their respective MICs, therefore, cell viability was not affected by the direct antibacterial activity of compounds. For compounds for which precipitation was observed after the addition of bacterial suspension in MH II broth, the highest concentration at which they did not precipitate was chosen. The final concentration of DMSO in the assays never exceed 2.5% *v/v* and had no influence on bacteria. In tests with *E. aerogenes*, a well-known efflux pump inhibitor, PAβN, was used as reference compound. The MICs of imidazolidine derivatives and antibiotics selected for experiments were recorded as the lowest concentrations of compounds inhibiting the growth of bacteria after 18-h incubation at 37 °C. Due to the heterogeneity of MRSA strains, the incubation of compounds with oxacillin was extended to 24 h. The growth of bacterial cells was visualized by iodonitrotetrazolium chloride (INT). The results of the MIC reduction assay were presented by employing activity gain parameter (A) calculated as the ratio of the MIC value of an antibiotic alone to its MIC in the presence of the compound analyzed (Equation (1)). All MIC determinations were repeated in at least two independent experiments

(1) $$A=\left(\frac{{\mathrm{MIC}}_{\mathrm{Ant}}}{{\mathrm{MIC}}_{\mathrm{Ant}+\mathrm{Comp}}}\right)$$

#### 3.3.2. Real-Time Efflux Assay

In the first step, bacteria were loaded with 1,2′-dNA, a fluorescent membrane probe which is a substrate of the AcrAB-TolC efflux pump, in the presence of a well-known EPI carbonyl cyanide m-chlorophenylhydrazone (CCCP) that inactivates the pump in the pump energy-collapsing mode (H^+^-consuming). After the addition of compounds, at the final concentration of 100 µM, an efflux was initiated by automated injection of glucose that provides pump energy (H^+^-donating compound) to the final concentration of 50 μM. The fluorescence intensity was measured using a microplate reader (Tecan) with an excitation wavelength of λ_ex_ = 370 nm and an emission wavelength of λ_em_ = 420 nm. In order to analyze and quantitatively compare the EPI activity of compounds tested, the pre-energization fluorescence intensity was adjusted to 100 relative fluorescence units and the inhibition efficiency (IE) of each compound was calculated according to Equation (2):

(2) $$\mathrm{IE}\;[\%]=\frac{\Delta i_{1}}{\Delta i_{2}}\times 100\%$$

where Δi_1_ corresponds to the difference between the fluorescence of the dye in the presence and absence of compound tested after the addition of a source of energy for the pump (glucose) and Δi_2_ is the difference between the fluorescence of the dye in the presence and absence of compound tested before the addition of a source of energy for the pump.

### 3.4. In Silico Studies

The three-dimensional conformations of compounds and respective protonation states (for pH 7.0 +/− 2.0) were generated with the use of LigPrep [49]. At first, all the compounds were docked to the crystal structure of PBP2a protein (PDB code: 3ZFZ [50]). Following the suggested mechanism of interaction of compounds via the allosteric modulation of this target [50,51], the studied compounds were docked to the active and allosteric sites of the protein (with grids were centered at S403 and S240, respectively; the docking was performed in Glide [52,53], and the compounds were docked in extra precision).

The poses with the best Glide docking score were use as starting points for molecular dynamic (MD) simulations. MD simulations were performed in Desmond [54,55], using TIP3P solvent model [56] and lasted 100 ns.

The interactions between ligands and PBP2a protein were analyzed manually and with the use of the Simulation Interaction Diagram from the Schrodinger Suite.

### 3.5. ADMET Studies

The references used in ADMET studies included caffeine (CFN), norfloxacin (NFX), doxorubicin (DX), carbonyl cyanide 3-chlorophenylhydrazone (CCCP), nonyl-4-hydroxyquinoline-*N*-oxide (NQNO) and were provided by Sigma-Aldrich (St. Louis, MO, USA). HepG2 (ATCC^®^ HB-8065™) *hepatoma* cell line was kindly donated by the Department of Pharmacological Screening, Jagiellonian University Medical College. *Salmonella typhimurium* TA100 strain with base pair substitution (hisG46 mutation, which target is GGG) was purchased from Xenometrix, Allschwil, Switzerland.

#### 3.5.1. Membrane Permeability

Pre-coated PAMPA Plate System Gentest™ was purchased from Corning (Tewksbury, MA, USA). The tested compound **9** and the references (200 µM) were prepared first in PBS buffer (pH = 7.4) and added to the donor wells of PAMPA Plate System. PBS was added to the acceptor wells. The plate was incubated for 5 h at room temperature. The compounds’ as well as references’ concentrations in acceptor and donor wells were estimated by the UPLC-MS analyses with use of internal standard. The permeability coefficients (*Pe*, cm/s) were calculated according to formulas provided by the manufacturer.

#### 3.5.2. Safety

Ames microplate fluctuation protocol (MPF) assay was obtained from Xenometrix AG (Allschwil, Switzerland). The occurrence of mutagen-induced or spontaneous reversion events to histidine prototrophy was determined as a growth of *S. typhimurium* TA100 in the indicator medium without histidine after 72 h incubation at 37 °C temperature. NQNO (0.5 µM) was used as a positive control. Compound **9** was tested in triplicate at the final concentrations 1 and 10 µM. The revertants’ growth induced the colour change of medium which was analysed next colourimetrically with a microplate reader (EnSpire, PerkinElmer, Waltham, MA, USA) at 420 nm.

The HepG2 cells were seeded in 96-well plates at a concentration of 1 × 10^4^ cells/well and incubated for 24 h at 37 °C in 5% CO_2_ atmosphere to reach 50% of confluence. Compound **9** was diluted into fresh growth medium and added to the cells at the final concentrations 0.1 μM–100 μM. The positive controls DX and CCCP were added at 1 μM and 10 μM, respectively, and the cells were incubated for 72 h. The MTS reagent (CellTiter 96^®^ AQueous One Solution Cell Proliferation Assay, Promega, Madison, WI, USA) was added next to the each well and incubated for 4 h. The absorbance was measured next using a microplate reader (EnSpire, PerkinElmer, Waltham, MA USA) at 490 nm to determine cells’ viability.

## 4. Conclusions

In order to search for successful antibiotic adjuvants, useful against both Gram-positive and Gram-negative MDR pathogens, the performed chemical modifications of the lead structure (*Z*)-5-(4-chlorobenzylidene)-2-(4-methylpiperazin-1-yl)-3*H*-imidazol-4(5*H*)-one (**6**) have provided a series of new, and even unexpected, chemical structures due to Dimroth rearrangements within the imidazolone ring. Most of those original compounds significantly improved oxacillin activity in MRSA, while they did not influence the activity of oxacillin in the reference *S. aureus* strain, suggesting an impact on MDR mechanisms associated with PBP2a. Docking and molecular dynamic simulations have confirmed this hypothesis, indicating the ability of the most active compounds to interact with the allosteric site of PBP2a, and to enhance the binding of oxacillin to the active site. On the other hand, results of the RTE assay for the new 5-arylideneimidazolones towards AcrAB-TolC in *E. aerogenes* (EA289) have confirmed their potent capacity to inhibit this important MDR efflux system. The performed SAR analysis indicated the anthracene-morpholine derivative (**16**) as the most active oxacillin adjuvant in the MRSA, and the naphthalene-methylpiperazine imidazolone (**9**) as the most potent “dual-action” compound displaying both oxacillin potentiating action in MRSA in the range of compound **16** and efflux pump inhibitory properties in *E. aerogenes*. The primary ADMET screening in vitro for **9** showed a rather moderate “drugability” profile for this active compound. In the context of the obtained results, further comprehensive studies for this interesting chemical family are needed. Overall, compound **9**, and then **16**, seem to be new lead structures for further modifications in order to discover therapeutically useful adjuvants, able to restore the effectiveness of common antibiotics against MDR pathogens.

## Figures and Tables

**Table 1:**
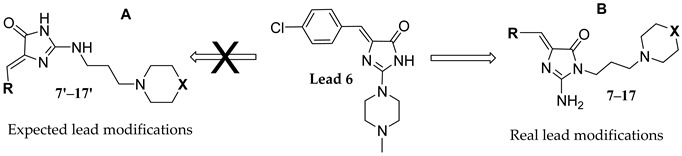
Structure of investigated compounds: **lead 6** and its modifications (**7**–**17**).

Cpd	R	X	Cpd	R	X
**6**	-	-	**12**	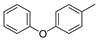	N-CH_3_
**7**	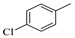	N-CH_3_	**13**	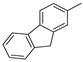	N-CH_3_
**8**	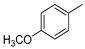	N-CH_3_	**14**	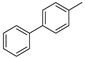	N-CH_3_
**9**	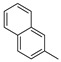	N-CH_3_	**15**	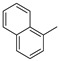	N-CH_3_
**10**	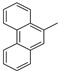	N-CH_3_	**16**	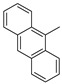	O
**11**	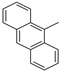	N-CH_3_	**17**	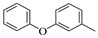	O

**Table 2:** Parameters of intramolecular and intermolecular interactions for compound **7**.

	D-H (Å)	H···A (Å)	D···A (Å)	D-H···A (°)	Symmetry Code
N5-H5B···N2	0.97(2)	1.96(2)	2.894(2)	160(2)	
N5-H5A···O1	0.90(2)	1.98(2)	2.846(2)	162(2)	x, −y+1/2, z+1/2
C13-H13B···CL1	0.96	2.87	3.576(3)	131.0	−x+1, y−1/2, −z+3/2

**Table 3:** Intrinsic antibacterial activity of compounds tested against *S. aureus* strains used in the study.

Compound.	MIC Value [mM]	
	MRSA HEMSA 5	*S. aureus* ATCC 25923
**6**	1	2
**7**	1	0.5–1
**8**	>2	>2
**9**	0.5	0.5
10 **	>0.125	>0.125
**11**	0.5	0.5
**12**	0.25–0.5	0.25
**13**	0.125	0.0625–0.125
**14**	0.25	0.12–0.25
**15**	1–2	1
16 **	>0.125	>0.125
**17**	0.25	0.25
**Oxacillin**	1.21 (512) *	0.00059 (0.25) *

* Activity of oxacillin was also evaluated in µg/mL concentration; ** compounds which precipitated after addition of bacterial suspension in cation-adjusted Mueller-Hinton (MH II) broth.

**Table 4:** MIC values obtained for compounds tested, PAβN and selected antibiotics against *E. aerogenes*.

Compound	MIC [mM]		
	Ea-294, Ea-298	Ea-289	CM-64
**9**	1	>2	>2
10 *	0.5	1	1
11 *, 17 *	0.5	>2	>2
**12**	1	2	2
**13**	0.25	0.5	0.5
16*	2	>2	>2
**PaβN**	0.125	0.5	0.5
**Chloramphenicol**	0.2 (64)	3.2 (1024)	1.6 (512)
**Erythromycin**	0.005 (4)	0.35 (256)	0.7 (512)
**Doxycycline**	0.005 (2)	0.018 (8)	0.072 (32)
**Norfloxacin**	0.05 (16)	0.2 (64)	0.006(2)

* Compounds, which precipitated after addition of bacterial suspension in MH II broth. Values in brackets represent concentrations of antibiotics in µg/mL.

**Table 5:** Effect of imidazolidine derivatives on the susceptibility of *S. aureus* strains to oxacillin.

Cpd	MRSA HEMSA 5			*S. aureus* ATCC 25923		
	Conc. of Compound [mM]	Activity Gain [A]	Range of Reduction [µg/mL]	Conc. of Compound [mM]	Activity Gain [A]	Range of Reduction [µg/mL]
**6**	0.25	8	from 512 to 64	0.5	2	from 0.25 to 0.125
**7**	0.25	16	from 512 to 32	0.125	1	no effect
**8**	0.5	8	from 512 to 64	0.5	1	no effect
**9**	0.125	16	from 512 to 32	0.125	2	from 0.25 to 0.125
**10**	0.125	8–16	from 512 to 32-64	0.125	1	no effect
**11**	0.125	8	from 512 to 64	0.125	2	from 0.25 to 0.125
**12**	0.0625	8	from 512 to 64	0.0625	2	from 0.25 to 0.125
**13**	0.0313	4–8	from 512 to 64-128	0.0156	1	no effect
**14**	0.0625	8	from 512 to 64	0.0313	2	from 0.25 to 0.125
**15**	0.25	4	from 512 to 128	0.25	1	no effect
**16**	0.125	16–32	from 512 to 16-32	0.125	1	no effect
**17**	0.0625	2–4	from 512 to 128-256	0.0625	2	from 0.25 to 0.125

**Table 6:** PAMPA results for compound **9** and the references: well-permeable caffeine (CFN) and low-permeable norfloxacin (NFX).

Comp.	*Pe* (10^−6^ cm/s) ± SD
CFN	15.1 ± 0.4
NFX	0.56 ± 0.1
**9**	0.72 ± 0.27

**Table 7:** The results of mutagenicity assay for **9** and the reference mutagen nonyl-4-hydroxyquinoline-*N*-oxide (NQNO).

Cpd 9 Concentration (µM)	n *	Mean of Revertants	SD	Fold Increase Over Baseline **	Binomial B-Value ***
0	9	9.00	3.00		
1	3	9.33	2.08	0.78	0.6331
10	3	5.33	4.73	0.44	0.0093
4-NQNO (0.5 µM)	9	45.00	4.50	3.75	1.0000

* number of replicates; ** compound is considered to be mutagenic if data points with fold increase ≥ 2; *** Binomial B-value ≤ 0.01 may be the result of compound’s cytotoxic effect.

## Supplementary Materials

The following are available online, Spectral data for compounds, 1H NMRs Compounds.

## References

[1] Tanwar J., Das S., Fatima Z., Hameed S. (2014). Multidrug resistance: An emerging crisis. Interdiscip. Perspect. Infect. Dis..

[2] Bassetti M., Righi E. (2015). Development of novel antibacterial drugs to combat multiple resistant organisms. Langenbecks Arch. Surg..

[3] World Health Organization. http://www.who.int/drugresistance/documents/surveillancereport/en/.

[4] Haysom L., Cross M., Anastasas R., Moore E., Hampton S. (2018). Prevalence and risk factors for methicillin-resistant *Staphylococcus aureus* (MRSA) infections in custodial populations: A systematic review. J. Correct. Heal. Care.

[5] Arêde P., Milheiriço C., De Lencastre H., Oliveira D.C. (2012). The anti-repressor MecR2 promotes the proteolysis of the mecA repressor and enables optimal expression of β-lactam resistance in MRSA. Plos Pathog..

[6] Ueda Y., Kanazawa K., Eguchi K., Takemoto K., Eriguchi Y., Sunagawa M. (2005). In vitro and in vivo antibacterial activities of SM-216601, a new broad-spectrum parenteral carbapenem. Antimicrob. Agents Chemother..

[7] Memmi G., Filipe S.R., Pinho M.G., Fu Z., Cheung A. (2008). *Staphylococcus aureus* PBP4 is essential for β -lactam resistance in community-acquired methicillin-resistant strains. Antimicrob. Agents Chemother..

[8] Yang C.C., Sy C.L., Huang Y.C., Shie S.S., Shu J.C., Hsieh P.H., Hsiao C.H., Chen C.J. (2018). Risk factors of treatment failure and 30-day mortality in patients with bacteremia due to MRSA with reduced vancomycin susceptibility. Sci. Rep..

[9] Foster T.J. (2017). Antibiotic resistance in *Staphylococcus aureus*. Current status and future prospects. FEMS Microbiol. Rev..

[10] Handzlik J., Szymańska E., Chevalier J., Otrębska-Machaj E., Kieć-Kononowicz K., Pagès J.-M., Alibert S. (2011). Amine-alkyl derivatives of hydantoin: New tool to combat resistant bacteria. Eur. J. Med. Chem..

[11] Handzlik J., Matys A., Kieć-Kononowicz K. (2013). Recent Advances in multi-drug resistance (MDR) efflux pump inhibitors of Gram-positive bacteria *S. aureus*. Antibiotics.

[12] Nikaido H. (2010). Multidrug resistance in bacteria. Annu. Rev. Biochem..

[13] Munita J.M., Arias C.A. (2016). Mechanisms of antibiotic resistance. Microbiol. Spectr..

[14] Nikaido H., Takatsuka Y. (2009). Mechanisms of RND multidrug efflux pumps. Biochim. Biophys. Acta.

[15] Bohnert J.A., Schuster S., Kern W.V., Karcz T., Olejarz A., Kaczor A., Handzlik J., Kieć-Kononowicz K. (2016). Novel piperazine arylideneimidazolones inhibit the AcrAB-TolC pump in *Escherichia coli* and simultaneously act as fluorescent membrane probes in a combined real-time influx and efflux assay. Antimicrob. Agents Chemother..

[16] González-Bello C. (2017). Antibiotic adjuvants—A strategy to unlock bacterial resistance to antibiotics. Bioorganic Med. Chem. Lett..

[17] Spengler G., Kincses A., Gajdács M., Amaral L. (2017). New Roads Leading to Old Destinations: Efflux Pumps as Targets to Reverse Multidrug Resistance in Bacteria. Molecules.

[18] Aron Z., Opperman T.J. (2018). The hydrophobic trap-the Achilles heel of RND efflux pumps. Res. Microbiol..

[19] Duraes F.A.P.M., Pinto M.M.M., de Sousa M.E.S.P. (2018). Medicinal Chemistry Updates on Bacterial Efflux Pump Modulators. Curr. Med. Chem..

[20] Leflon-Guibout V., Speldooren V., Heym B., Nicolas-Chanoine M.H. (2000). Epidemiological survey of amoxicillin-clavulanate resistance and corresponding molecular mechanisms in *Escherichia coli* isolates in France: New genetic features of bla(TEM) genes. Antimicrob. Agents Chemother..

[21] Bernal P., Molina-Santiago C., Daddaoua A., Llamas M. (2013). Antibiotic adjuvants: Identification and clinical use. Microb. Biotechnol..

[22] Ventola C.L. (2015). The Antibiotic Resistance Crisis Part 1: Causes and Threats. Pharm. Ther..

[23] Gill E.E., Franco O.L., Hancock R.E.W. (2015). Antibiotic adjuvants: Diverse strategies for controlling drug-resistant pathogens. Chem. Biol. Drug Des..

[24] Melander R., Melander C. (2017). The Challenge of Overcoming Antibiotic Resistance: An Adjuvant Approach?. ACS Infect Dis..

[25] Matys A., Podlewska S., Witek K., Witek J., Bojarski A., Schabikowski J., Otrębska-Machaj E., Latacz G., Szymańska E., Kieć-Kononowicz K. (2015). Imidazolidine-4-one derivatives in the search for novel chemosensitizers of *Staphyloccocus aureus* MRSA: Synthesis, biological evaluation and molecular modelling studies. Eur. J. Med. Chem..

[26] Żesławska E., Nitek W., Tejchman W., Handzlik J. (2018). Influence of 3-{5-[4-(diethylamino)benzylidene]rhodanine}propionic acid on the conformation of 5-(4-chlorobenzylidene)-2-(4-methylpiperazin-1-yl)-3H-imidazol-4(5H)-one. Acta Crystallogr. C Struct. Chem..

[27] Kim H.Y., Finneman J.I., Harris C.M., Harris T.M. (2000). Studies of the mechanisms of adduction of 2′-deoxyadenosine with styrene oxide and polycyclic aromatic hydrocarbon dihydrodiol epoxides. Chem. Res. Toxicol..

[28] Żesławska E., Nitek W., Handzlik J. (2017). Conformational study of (Z)-5-(4-chlorobenzylidene)-2-[4-(2-hydroxyethyl)piperazin-1-yl]-3H-imidazol-4(5H)-one in different environments: Insight into the structural properties of bacterial efflux pump inhibitors. Acta Crystallogr. C Struct. Chem..

[29] Groom C.R., Bruno I.J., Lightfoot M.P., Ward S.C. (2016). The Cambridge Structural Database. Acta Cryst. B Struct. Sci. Cryst. Eng. Mater..

[30] Bernstein J., Davis R.E., Shimoni L., Chang N.L. (1995). Patterns in hydrogen bonding: Functionality and graph set analysis in crystals. Angew. Chem. Int. Ed. Engl..

[31] Otrębska-Machaj E., Chevalier J., Handzlik J., Szymańska E., Schabikowski J., Boyer G., Bolla J.-M., Kieć-Kononowicz K., Pages J.-M., Alibert S. (2016). Efflux pump blockers in Gram-negative bacteria: The new generation of hydantoin based-modulators to improve antibiotic activity. Front. Microbiol..

[32] Bohnert A., Schuster S., Szymaniak-Vits M., Kern W.V. (2011). Determination of real-time efflux phenotypes in *Escherichia coli* AcrB binding pocket phenylalanine mutants using a 1, 2′ dinaphthylamine efflux assay. PLoS ONE.

[33] Lôme V., Brunel J.M., Pagès J.-M., Bolla J.M. (2018). Multiparametric Profiling for Identification of Chemosensitizers against Gram-Negative Bacteria. Front. Microbiol..

[34] Chen H., Ahsan S.S., Santiago-Berrios M.E.B., Abruña H.D., Webb W. (2010). Mechanism of quenching of Alexa fluorophores by natural amino acids. J. Am. Chem. Soc..

[35] Chen X.X., Murawski A., Patel K., Crespi C.L., Balimane P.V. (2008). A novel design of artificial membrane for improving the PAMPA model. Pharm. Res..

[36] Latacz G., Hogendorf A.S., Hogendorf A., Lubelska A., Wierońska J.M., Woźniak M., Cieślik P., Kieć-Kononowicz K., Handzlik J., Bojarski A.J. (2018). Search for a 5-CT alternative. In vitro and in vivo evaluation of novel pharmacological tools: 3-(1-alkyl-1*H*-imidazol-5-yl)-1*H*-indole-5-carboxamides, low-basicity 5-HT_7_ receptor agonists. MedChemComm.

[37] Flückiger-Isler S., Baumeister M., Braun K., Gervais V., Hasler-Nguyen N., Reimann R., Van Gompel J., Wunderlich H.G., Engelhardt G. (2004). Assessment of the performance of the Ames II TM assay: A collaborative study with 19 coded compounds. Mutat. Res..

[38] Handzlik J., Spengler G., Mastek B., Dela A., Molnar J., Amaral L., Kieć-Kononowicz K. (2012). 5-Arylidene(thio)hydantoin derivatives as modulators of Cancer efflux pump. Acta Pol. Pharm. Drug Res..

[39] Rempel V., Atzler K., Behrenswerth A., Karcz T., Schoeder C., Hinz S., Kaleta M., Thimm D., Kieć-Kononowicz K., Mueller C.E. (2014). Bicyclic imidazole-4-one derivatives: A new class of antagonists for the orphan G protein-coupled receptors GPR18 and GPR55. Med. Chem. Commun..

[40] Szymańska E., Kieć-Kononowicz K. (2002). Antimycobacterial activity of 5-arylidene derivatives of hydantoin. Farmaco.

[41] Khan S., Tyagi V., Mahar R., Bajpai V., Kumar B., Chauhan P.M.S. (2013). Expedient base-mediated desulfitative dimethylamination, oxidation, or etherification of 2-(methylsulfanyl)-3,5-dihydro-4H-imidazol-4-one scaffolds. Synthesis.

[42] Łażewska D., Maludziński P., Szymańska E., Kieć-Kononowicz K. (2007). The lipophilicity estimation of 5-arylidene derivatives of (2-thio)hydantoin with antimycobacterial activity. Biomed. Chromatogr..

[43] Altomare A., Burla M.C., Camalli M., Cascarano G.L., Giacovazzo C., Guagliardi A., Moliterni A.G.G., Polidori G., Spagna R.J. (1999). SIR97: A new tool for crystal structure determination and refinement. J. Appl. Cryst..

[44] Sheldrick G.M. (2015). Crystal structure refinement with SHELXL. Acta Cryst..

[45] Farrugia L.J. (2012). WinGX and ORTEP for Windows: An update. J. Appl. Cryst..

[46] Macrae C.F., Edgington P.R., McCabe P., Pidcock E., Shields G.P., Taylor R., Towler M., van de Streek J. (2006). Mercury: Visualization and Analysis of Crystal Structures. J. Appl. Cryst..

[47] Clinical and Laboratory Standards Institute (2012). Document M07-A9 Methods for Dilution Antimicrobial Susceptibility Tests for Bacteria that Grow Aerobically, Approved Standard.

[48] Members of the Antibiogram Committee (2003). Comité de l’Antibiogramme de la Société Française de Microbiologie. Report 2003. Int. J. Antimicrob. Agents.

[49] (2016). Schrödinger Release 2016-4: LigPrep.

[50] Otero L.H., Rojas-Altuve A., Llarrull L.I., Carrasco-Lopez C., Kumarasiri M., Lastochkin E., Fishovitz J., Dawley M., Hesek D., Lee M. (2013). How allosteric control of *Staphylococcus aureus* penicillin binding protein 2a enables methicillin resistance and physiological function. Proc. Natl. Acad. Sci. USA.

[51] Mahasenan K.V., Molina R., Bouley R., Batuecas M.T., Fisher J.F., Hermoso J.A., Chang M., Mobashery S. (2017). Conformational Dynamics in Penicillin-Binding Protein 2a of Methicillin-Resistant *Staphylococcus aureus*, Allosteric Communication Network and Enablement of Catalysis. J. Am. Chem. Soc..

[52] Friesner R.A., Banks J.L., Murphy R.B., Halgren T.A., Klicic J.J., Mainz D.T., Repasky M.P., Knoll E.H., Shelley M., Perry J.K. (2004). Glide: A new approach for rapid, accurate docking and scoring. 1. Method and assessment of docking accuracy. J. Med. Chem..

[53] (2016). Schrödinger Release 2016-4: Glide.

[54] (2016). Schrödinger Release 2016-4: Desmond Molecular Dynamics System.

[55] (2016). Maestro-Desmond Interoperability Tools.

[56] Jorgensen W.L., Chandrasekhar J., Madura J.D., Impey R.W., Klein M.L. (1983). Comparison of simple potential functions for simulating liquid water. J. Chem. Phys..

