# Supplementary material for: 5-Arylideneimidazolones with Amine at Position 3 as Potential Antibiotic Adjuvants against Multidrug Resistant Bacteria
Source: Molecules. 2019 Jan 26;24(3):438. doi: 10.3390/molecules24030438 (PMC6384991; doi:10.3390/molecules24030438)
Supplement: Supplementary file 1 [file molecules-24-00438-s001.pdf]

5-Arylideneimidazolones with Amine at Position 3 as Potential Antibiotic Adjuvants against Multidrug Resistant Bacteria

Aneta Kaczor[1], Karolina Witek[1,2,3], Sabina Podlewska[1,4], Joanna Czekajewska[2], Annamaria Lubelska[1], Gniewomir Latacz[1], Ewa Żesławska[5], Wojciech Nitek[6], Sandrine Alibert[3], Jean-Marie Pagès[3], Elżbieta Karczewska[2], Katarzyna Kieć- Kononowicz[1], Jadwiga Handzlik[1,*]

Supplementary

Spectral data for compounds

^{1}H NMRs Compounds

Compound 7 (basic form)

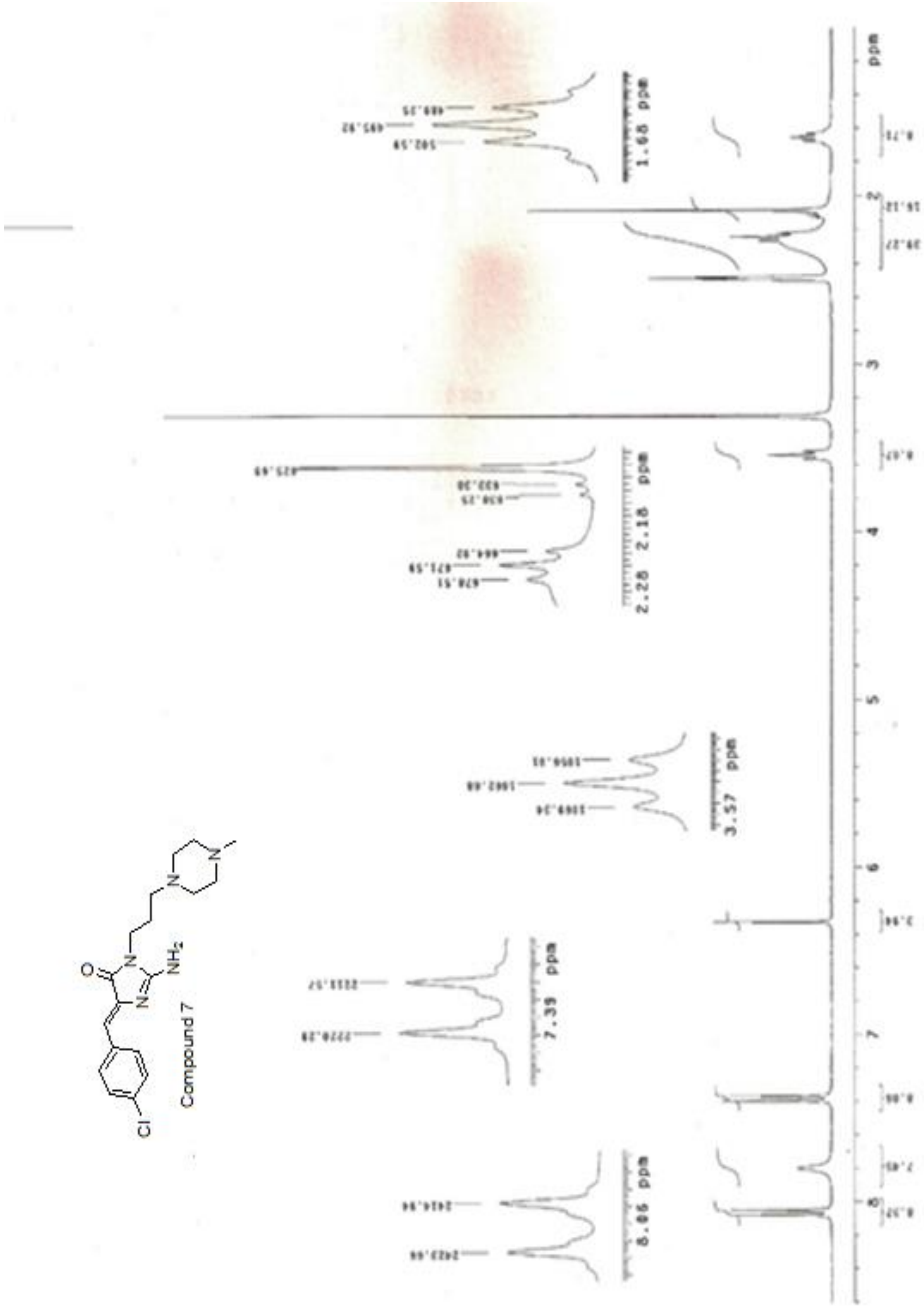

[1]**H-NMR** for **7** (DMSO-d_6) δ [ppm]: 1.63 (qu, J=6.67 Hz, 2H, CH_2-$\underline{CH_2}$-CH_2), 2.09 (s, 3H, CH_3), 2.22-2.26 (t def., 10H, Pp, Pp-CH_2), 3.52 (t, J=6.67 Hz, 2H, N3-CH_2), 6.32(s, 1H, CH=C), 7.36 (d, J=8.72Hz, 2H, Ar-3,5-H), 7.80 (br. s, 2H, NH_2), 8.05 (d, J=8.72 Hz, 2H, Ar-2,6-H).

Compound 7 (HCl form)

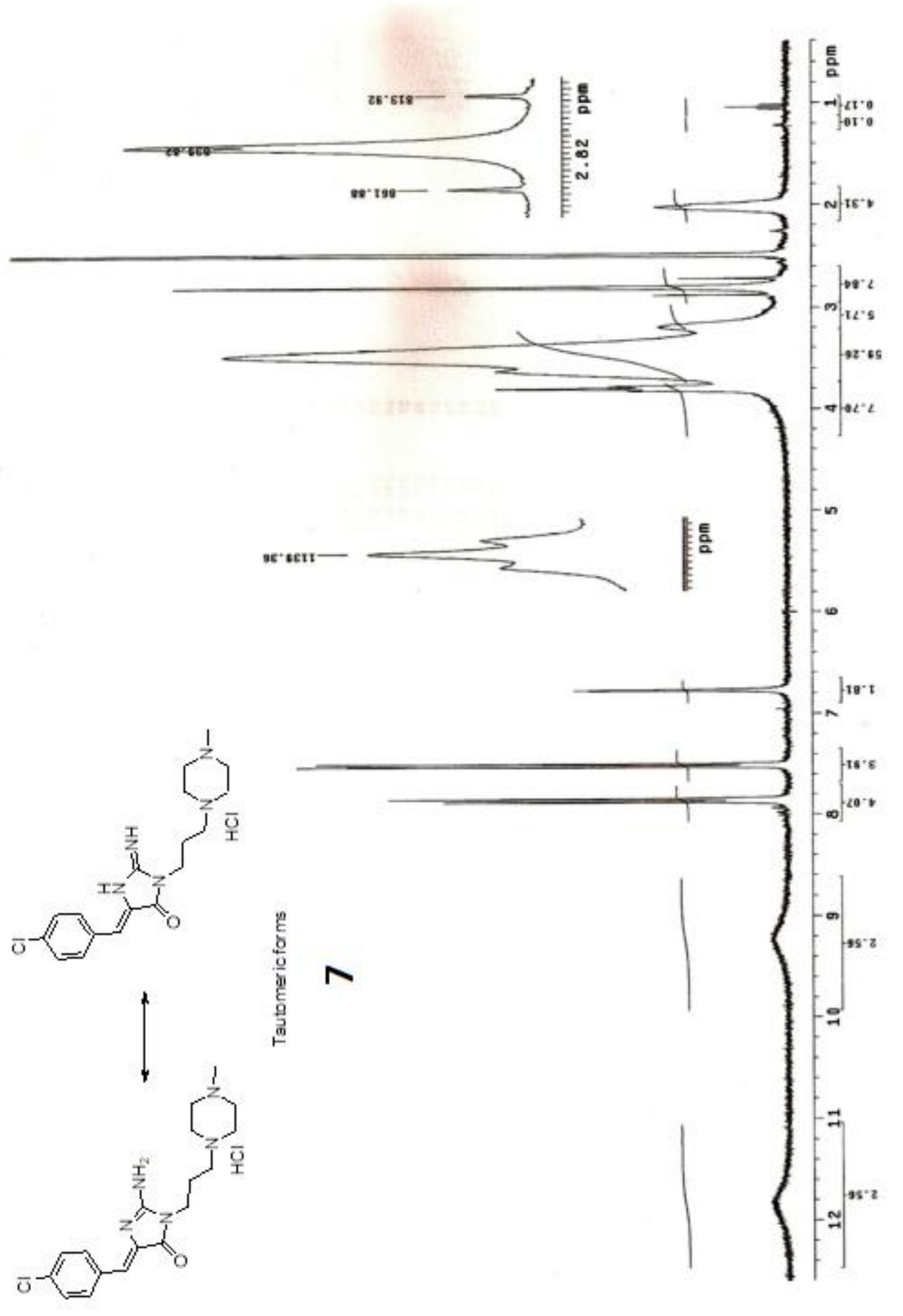

Compound 8 (basic form)

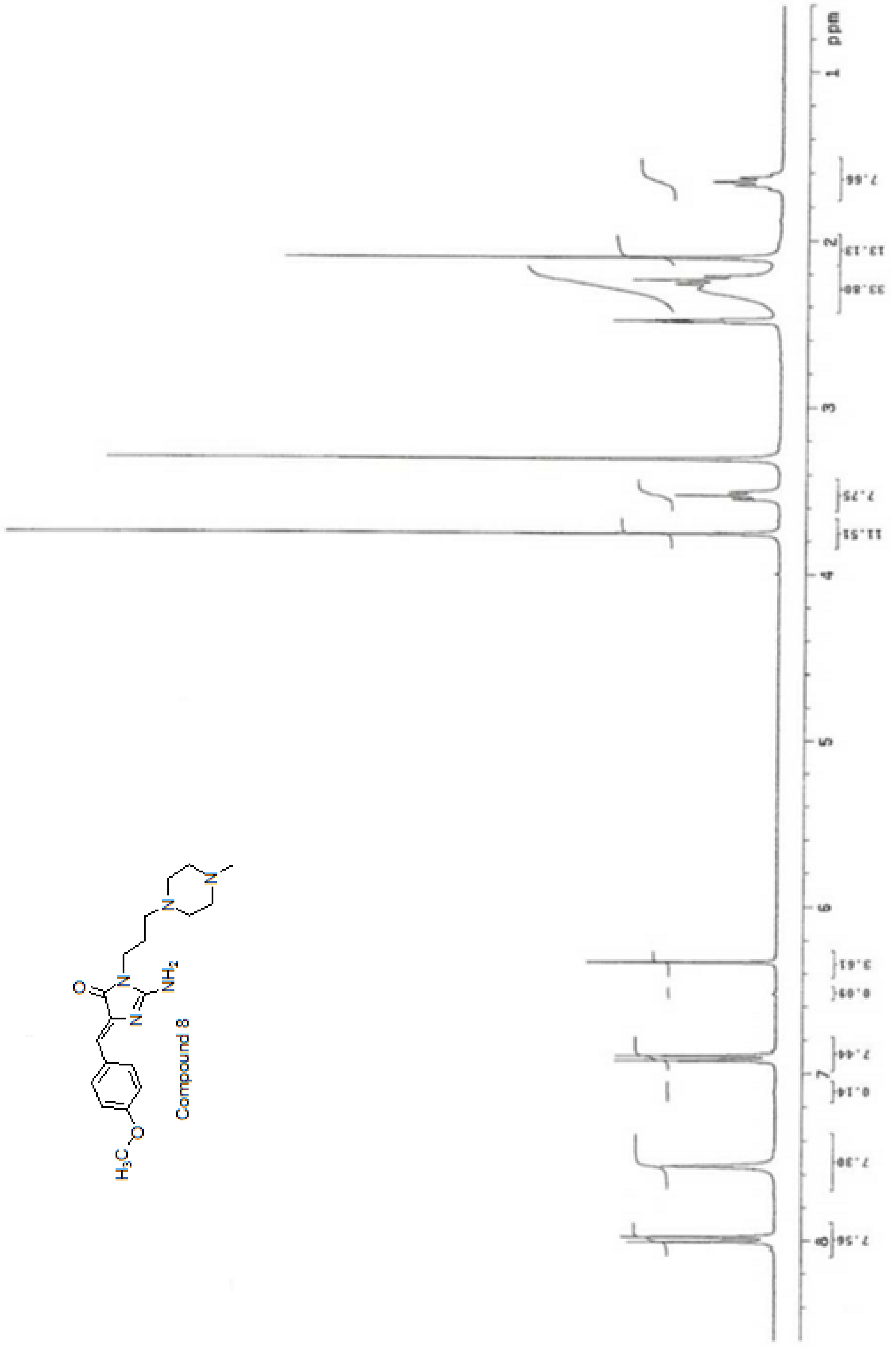

[1]**H-NMR** for **8** (DMSO-d_6) δ [ppm]: 1.63 (t, J=6.67 Hz, 2H, CH_2-<u>CH_2</u>-CH_2), 2.10 (s, 3H, N-CH_3), 2.21-2.40 (m, 8H, Pp, Pp-CH_2), 3.50 (t, J=6.70 Hz, 2H, N3-CH_2), 3.76 (s, 3H, OCH_3), 6.33 (s, 1H, CH=C), 6.89 (d, J=8.98, 2H, Ar-3,5-H), 7.55 (s, 2H, NH_2), 7.98 (d, J=8.72 Hz, 2H, Ar-2,6-H).

Compound 8 (HCl form)

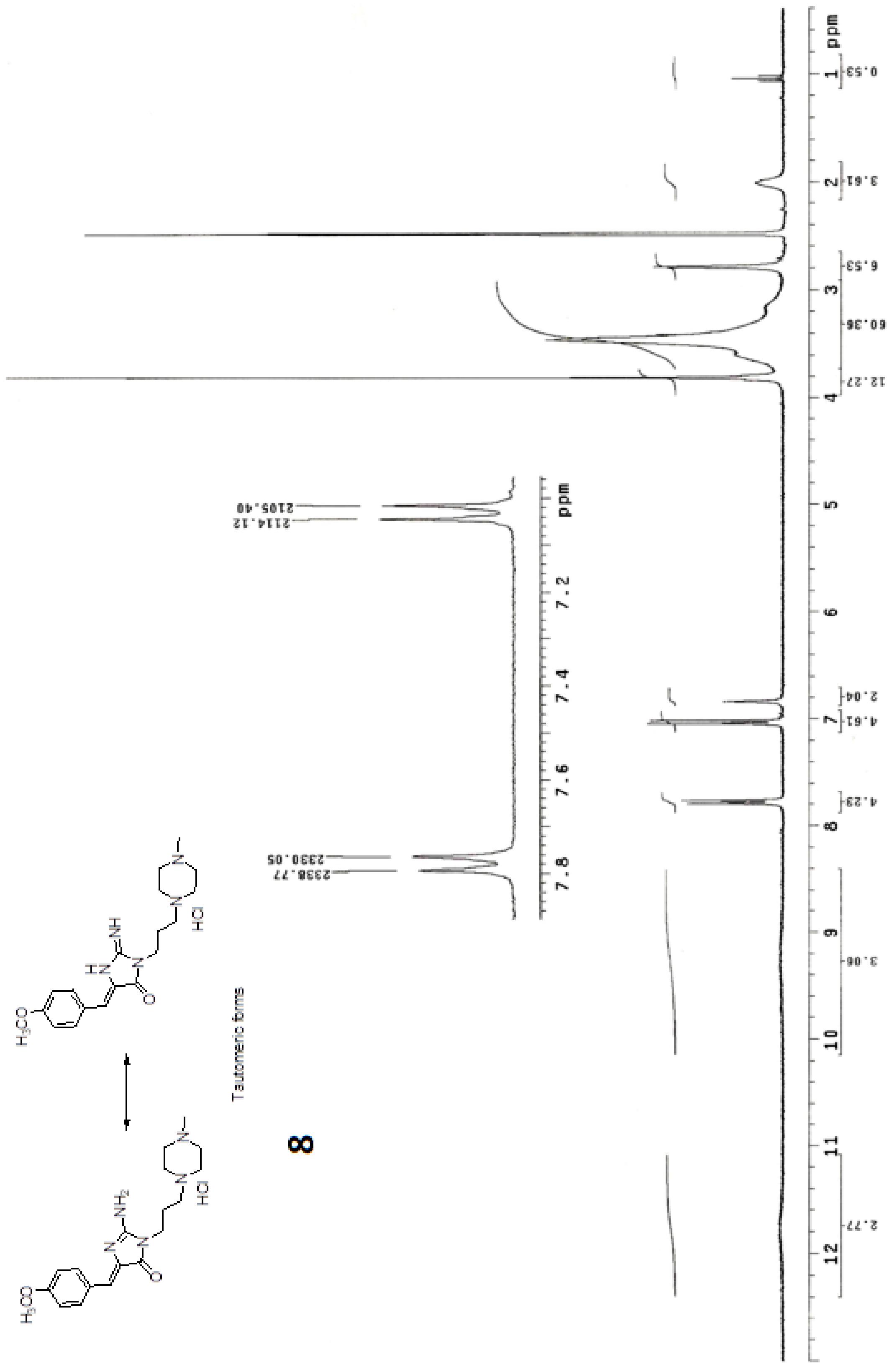

Compound 9

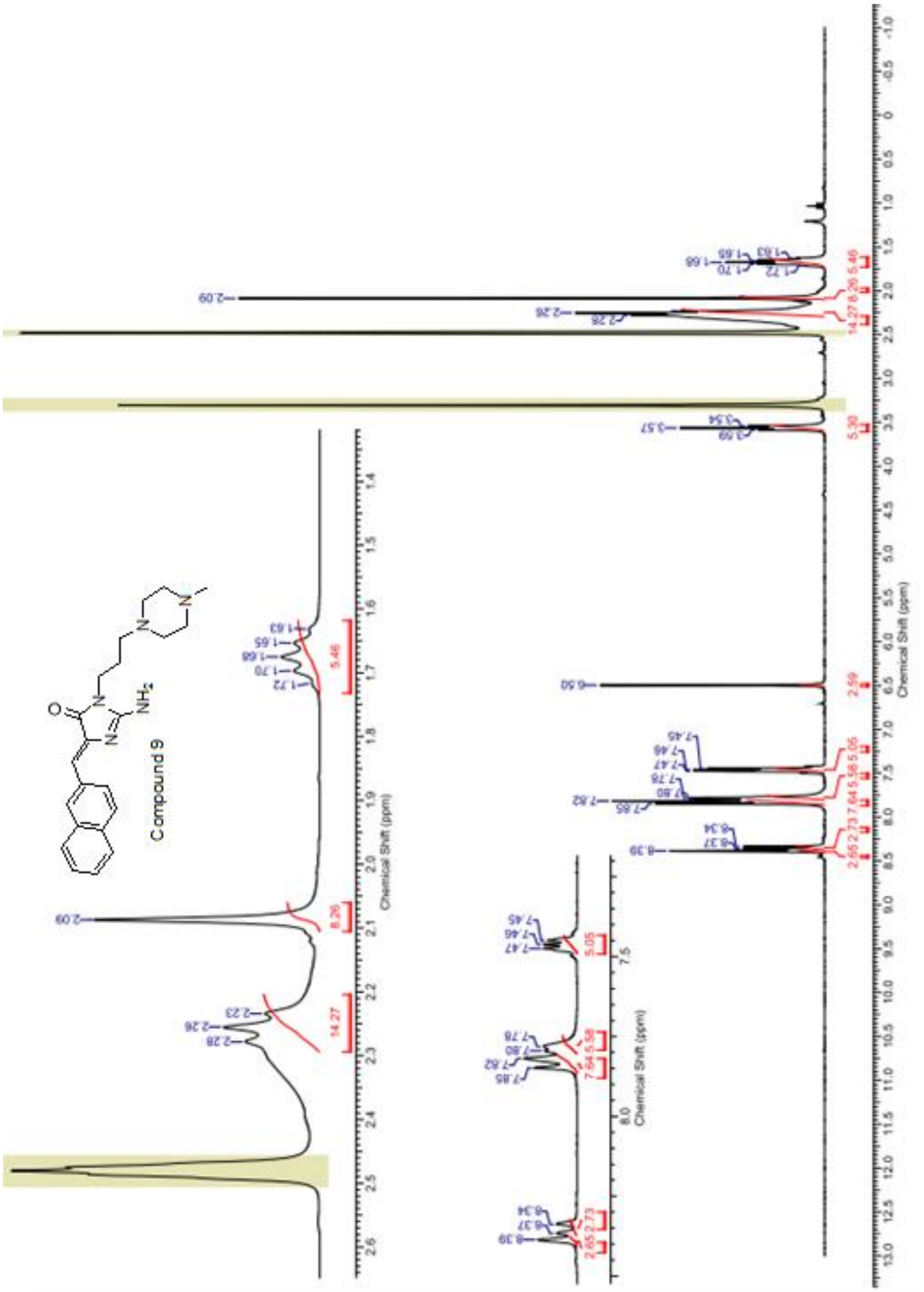

Compound 10

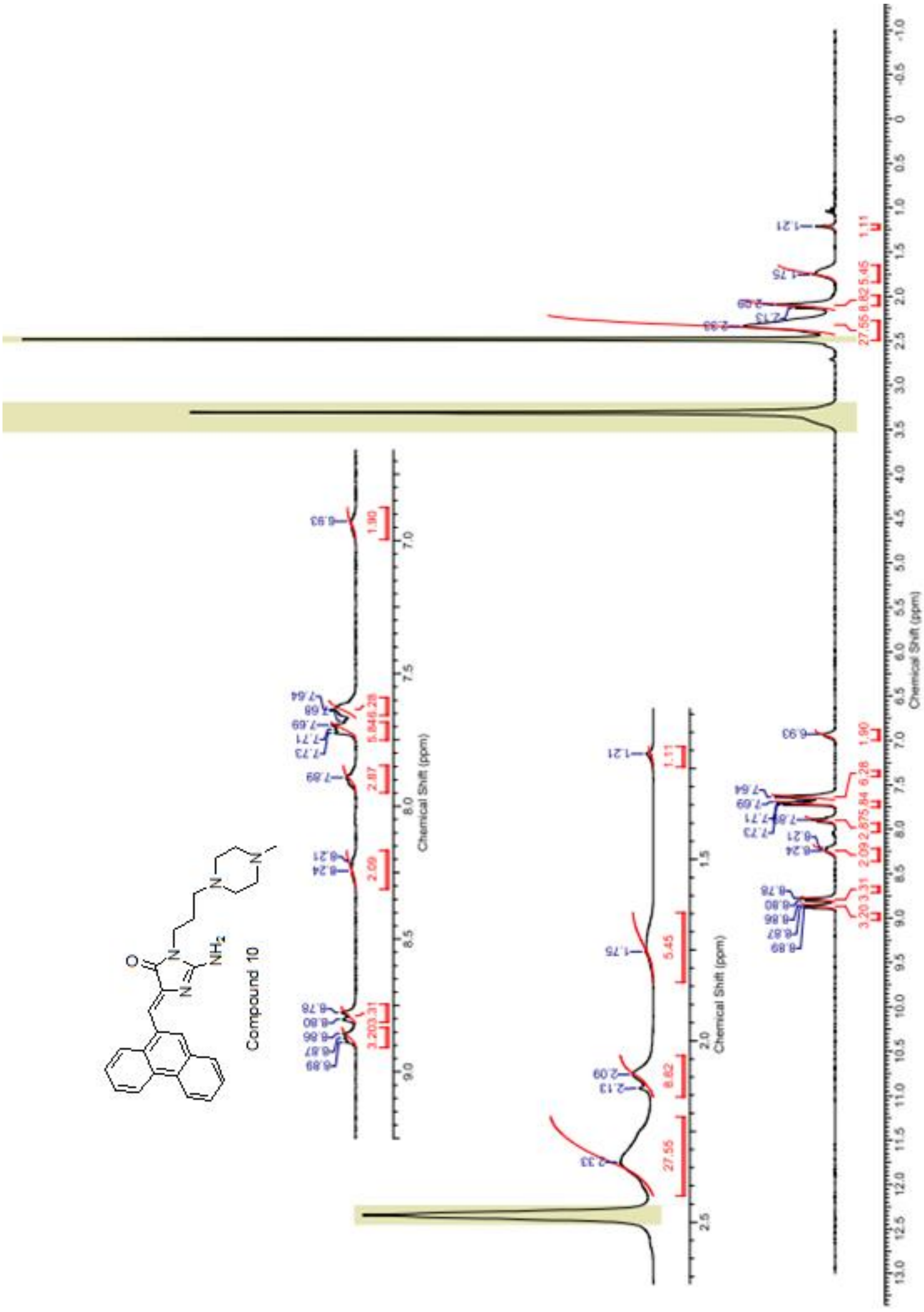

Compound 11

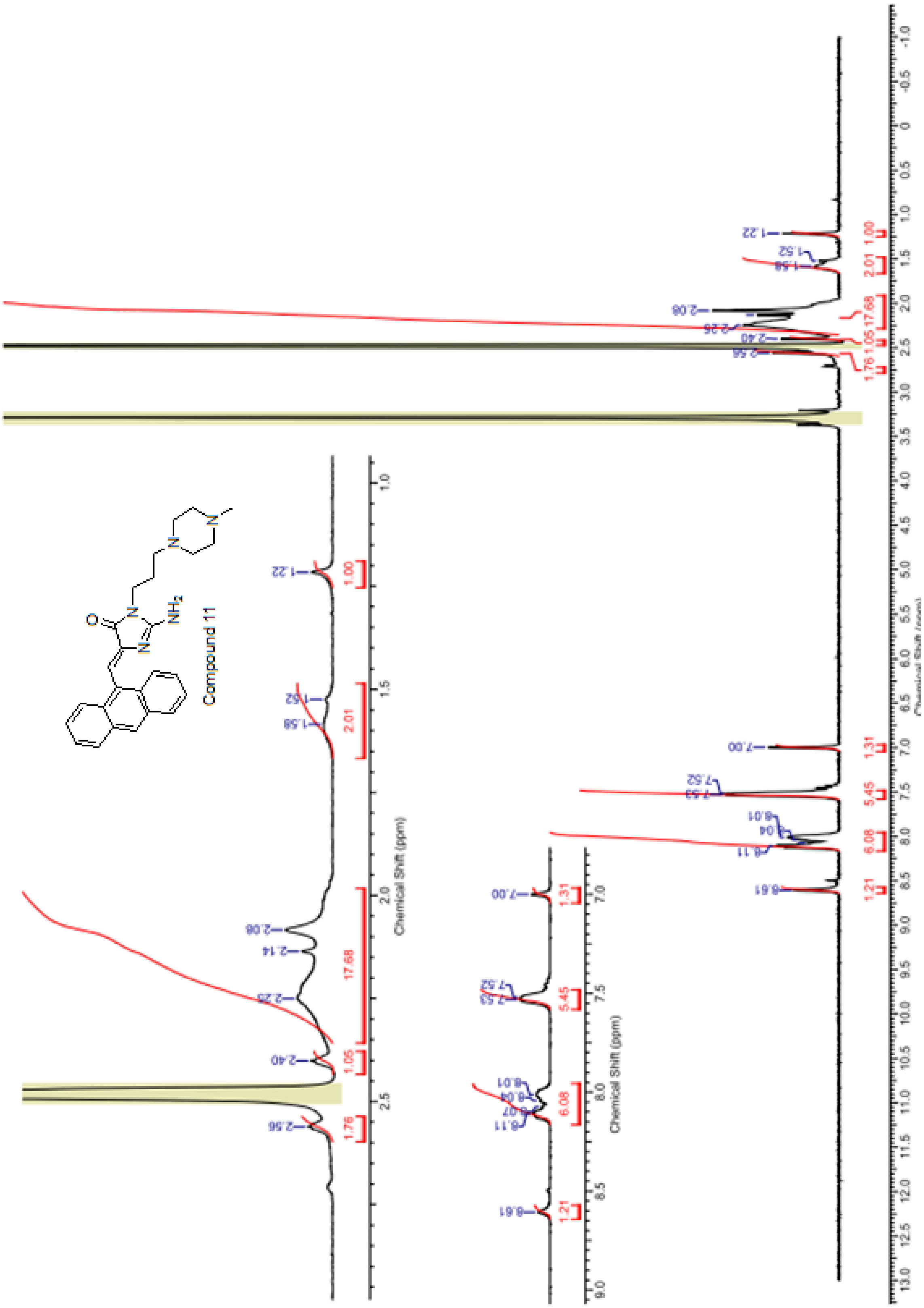

Compound 12

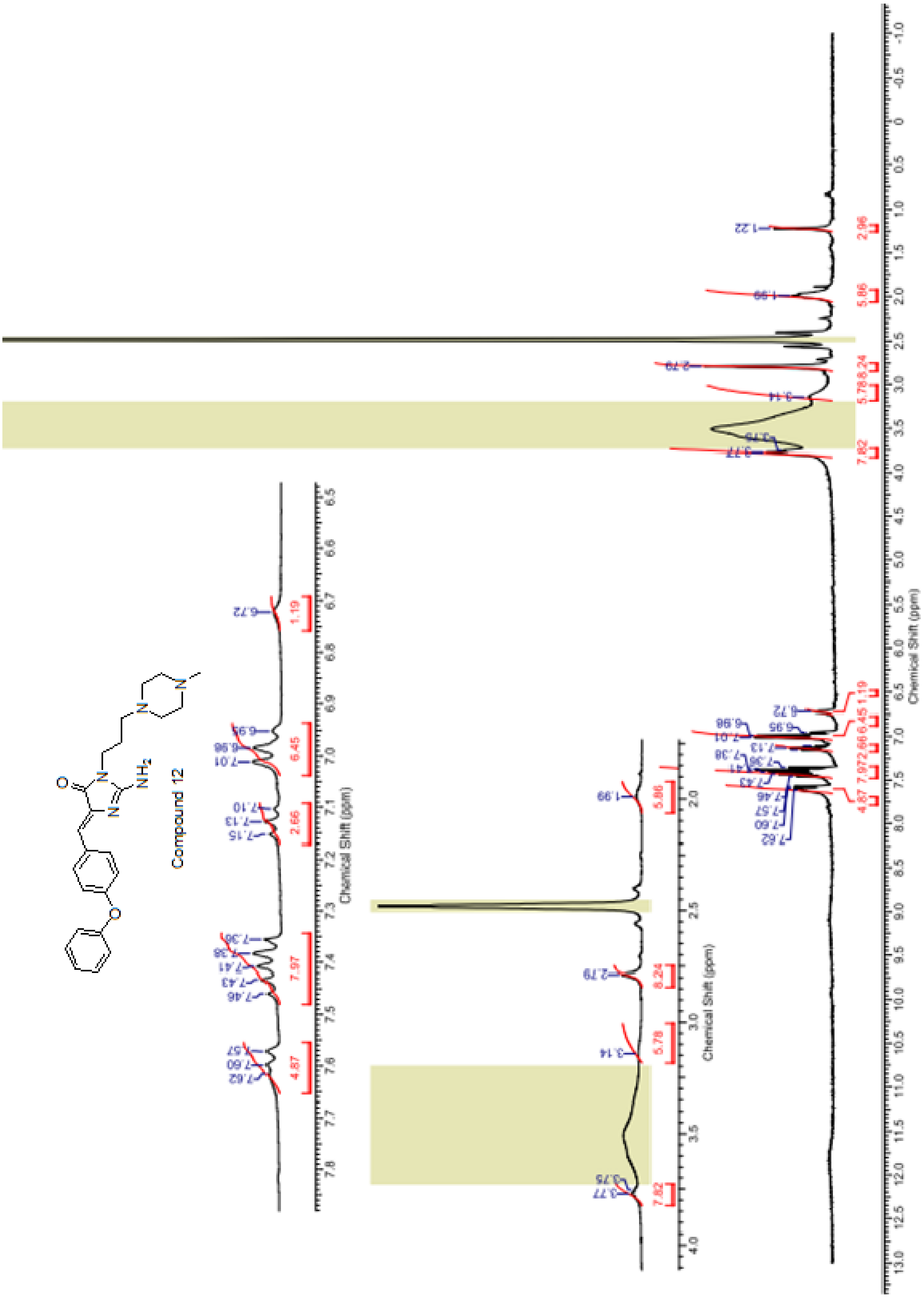

Compound 13

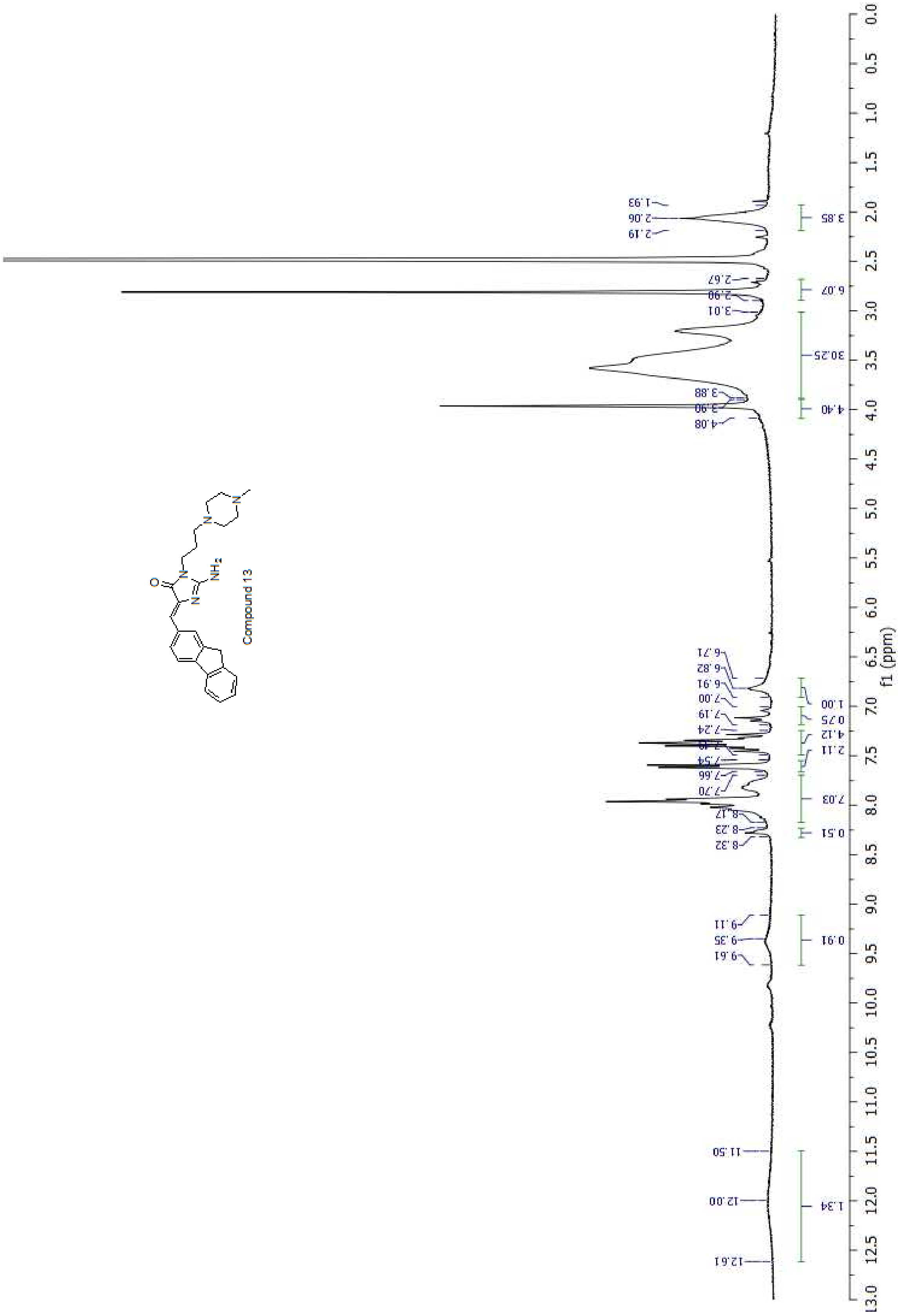

Compound 14

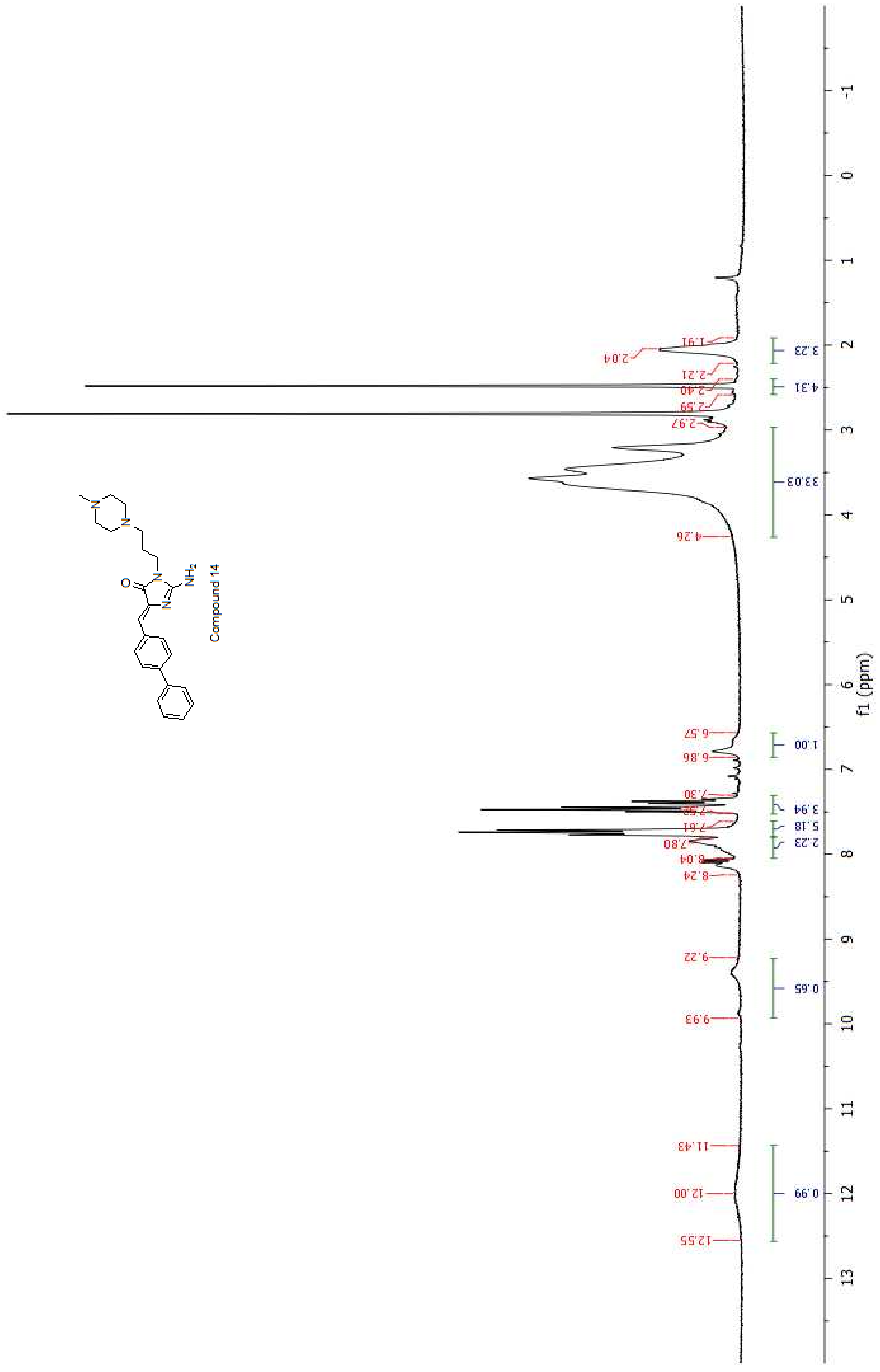

Compound 15

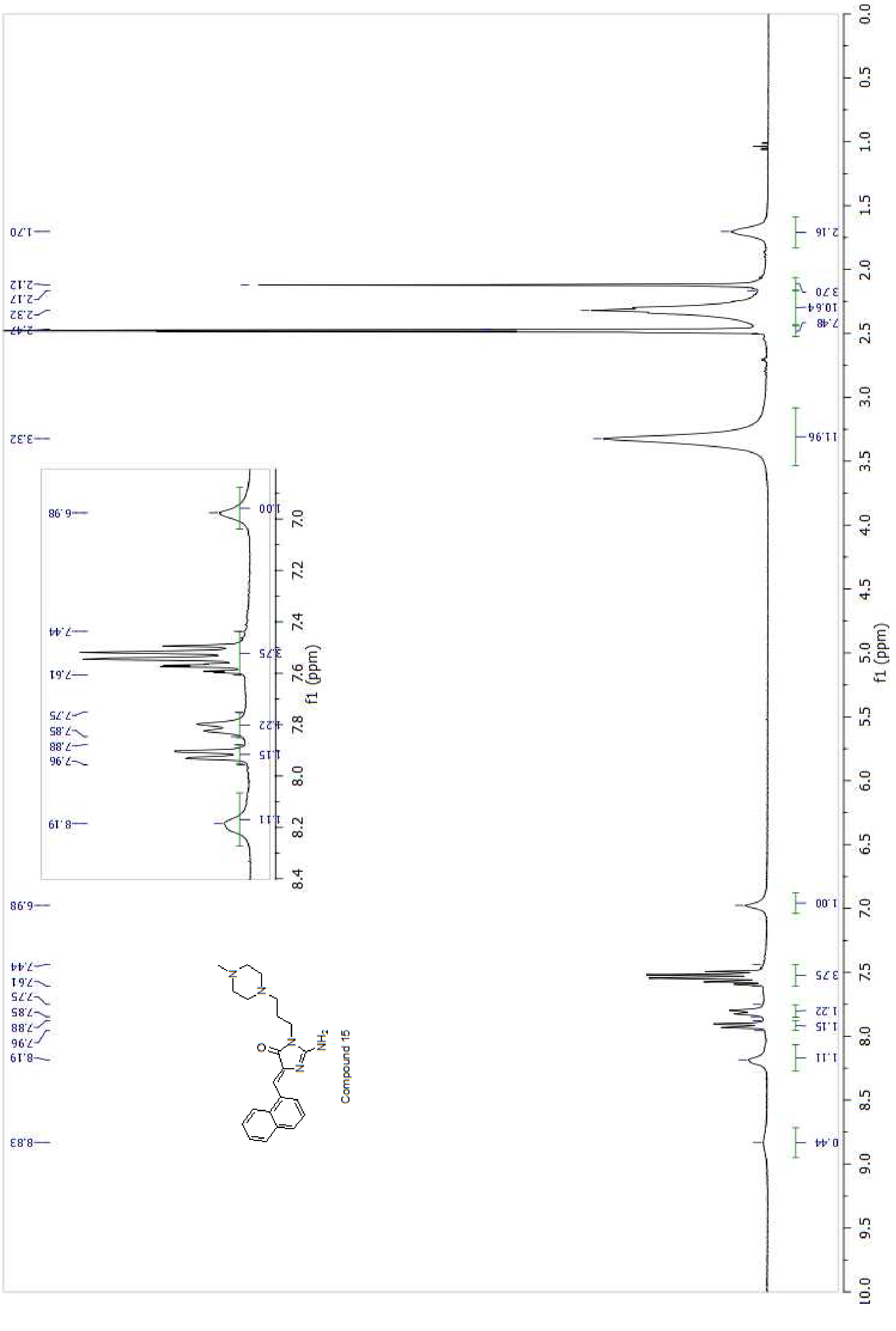

1.70
2.12
2.17
2.32
2.47
3.32
6.98
7.44
7.61
7.75
7.85
7.88
7.96
8.19
8.83
2.16
3.70
10.64
7.48
11.96
1.00
3.75
1.22
1.15
1.11
0.44
f1 (ppm)
Compound 15

Compound 16

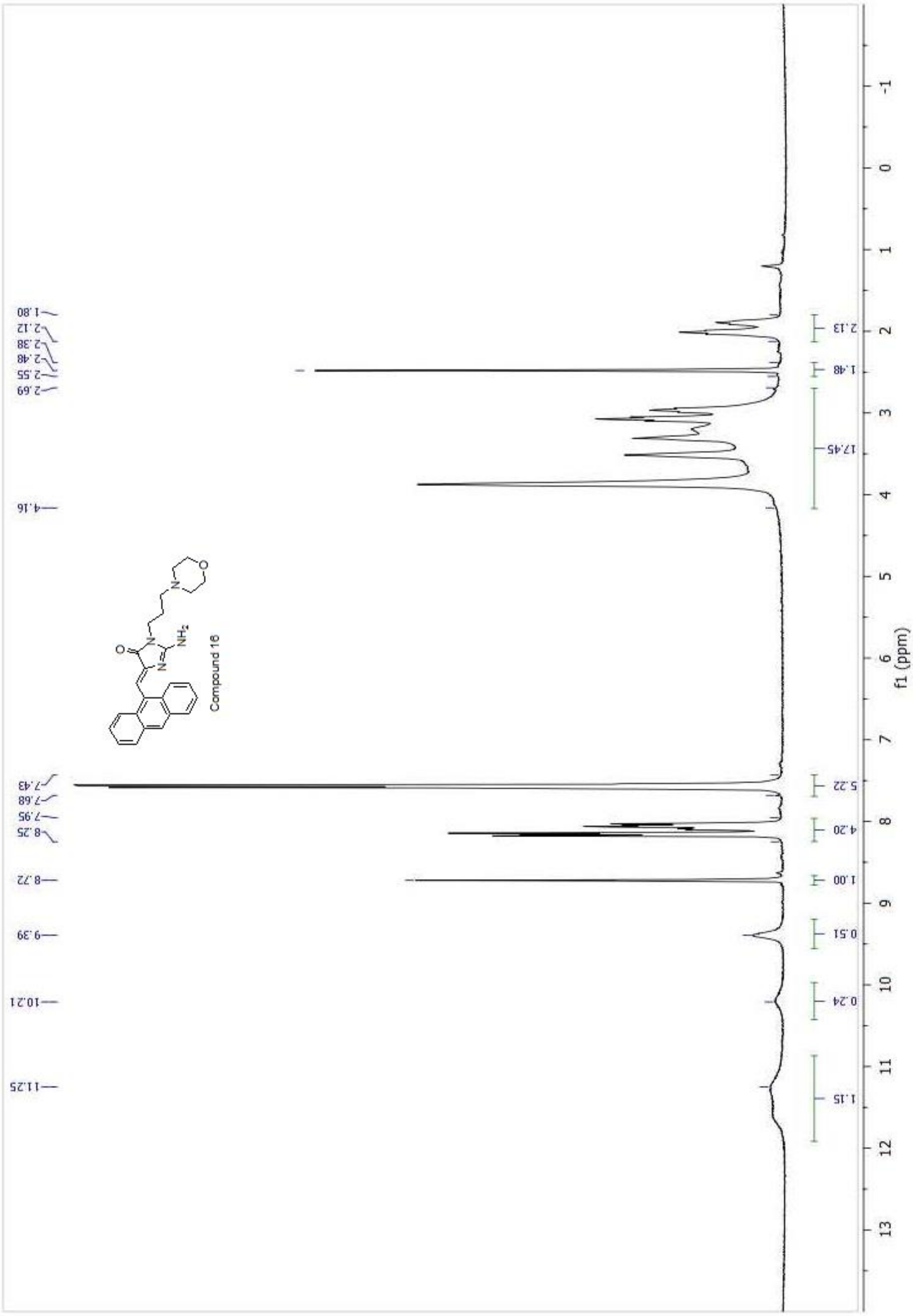
Compound 16
NH2
O
N
f1 (ppm)
1.80
2.12
2.38
2.48
2.55
2.69
4.16
7.43
7.68
7.95
8.25
8.72
9.39
10.21
11.25
2.13
1.48
17.45
5.22
4.20
1.00
0.51
0.24
1.15

Compound 17

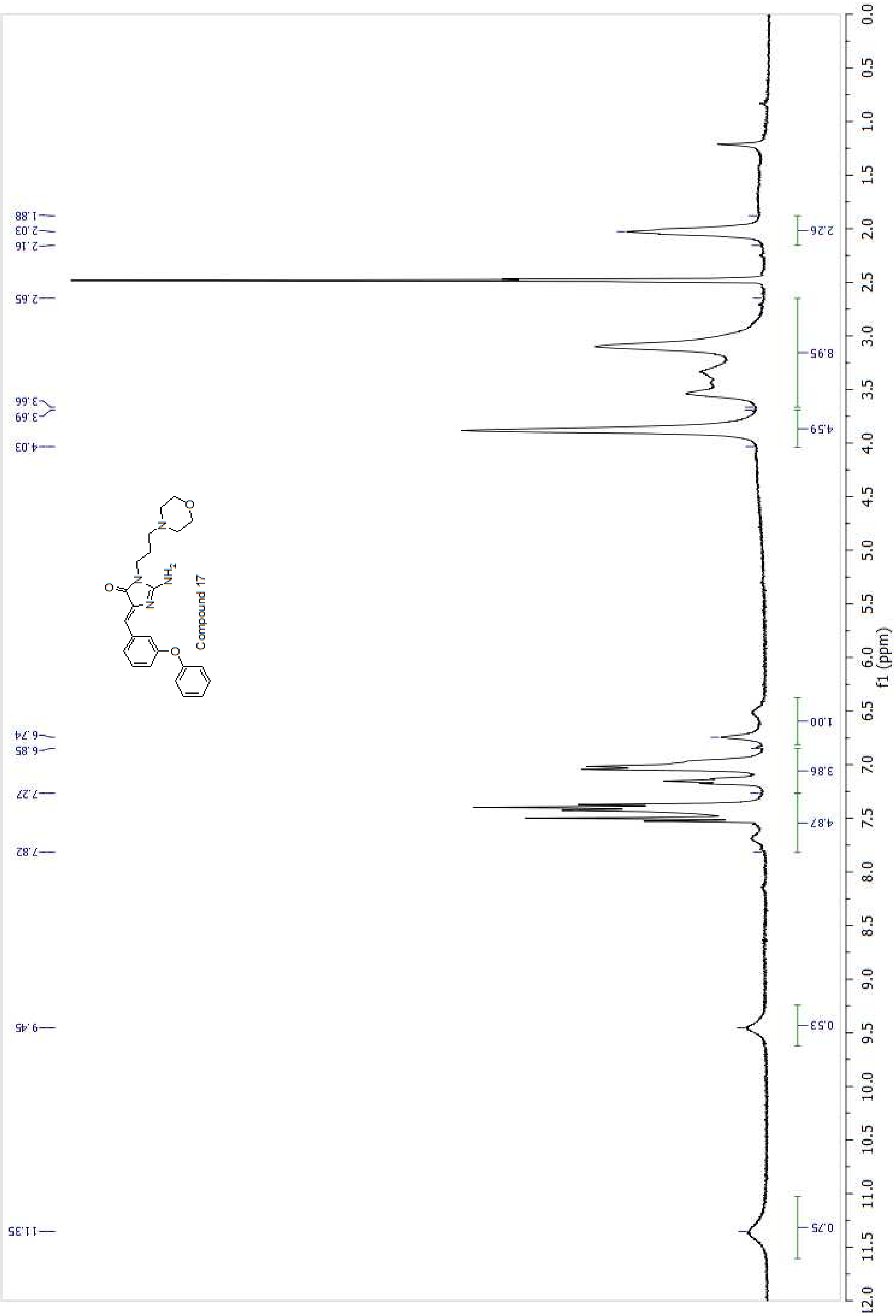
1.88
2.03
2.16
2.65
3.66
3.69
4.03
6.74
6.85
7.27
7.82
9.45
11.35
2.26
8.95
4.59
1.00
3.86
4.87
0.53
0.75
Compound 17
f1 (ppm)

Compound 21

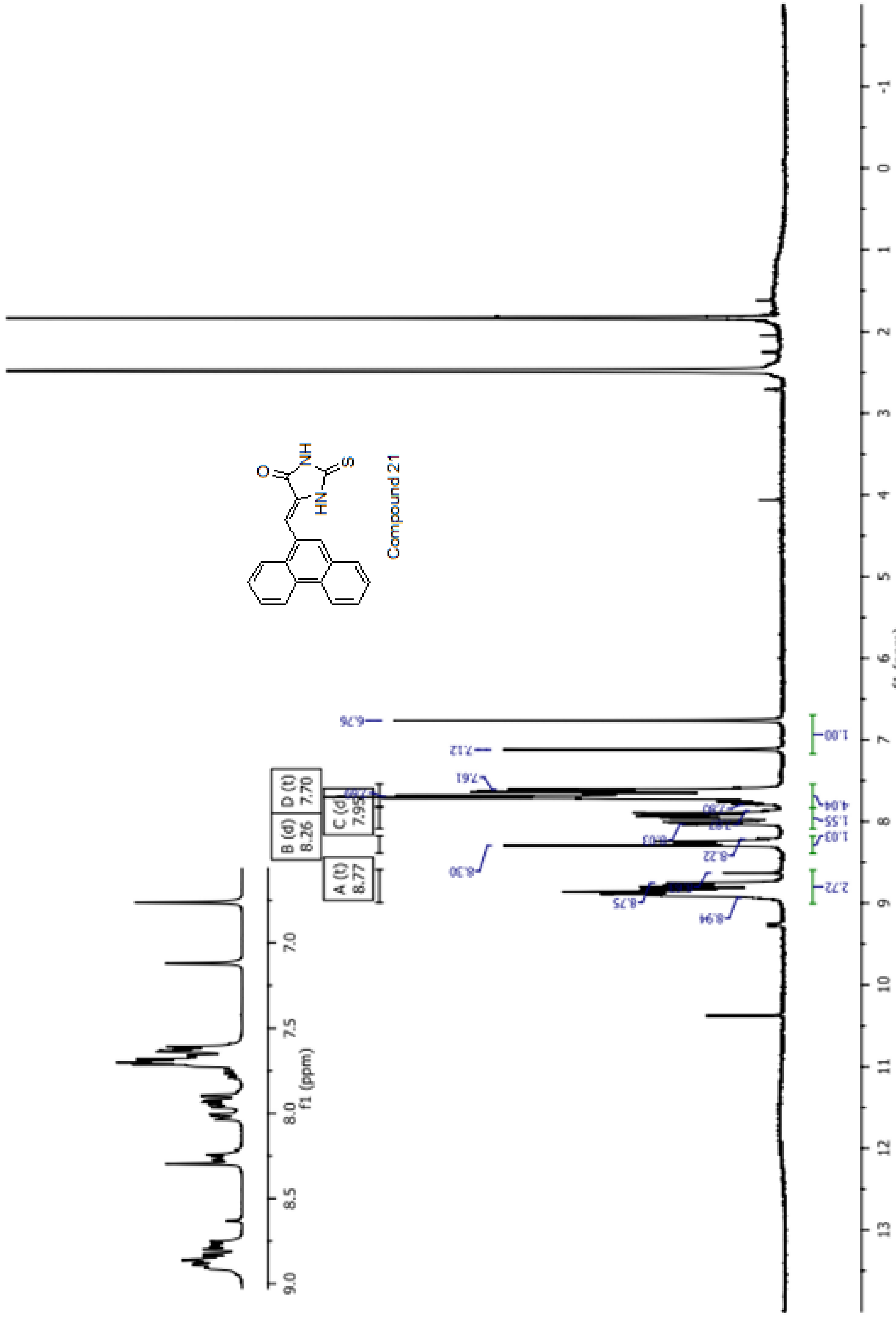

Compound 23

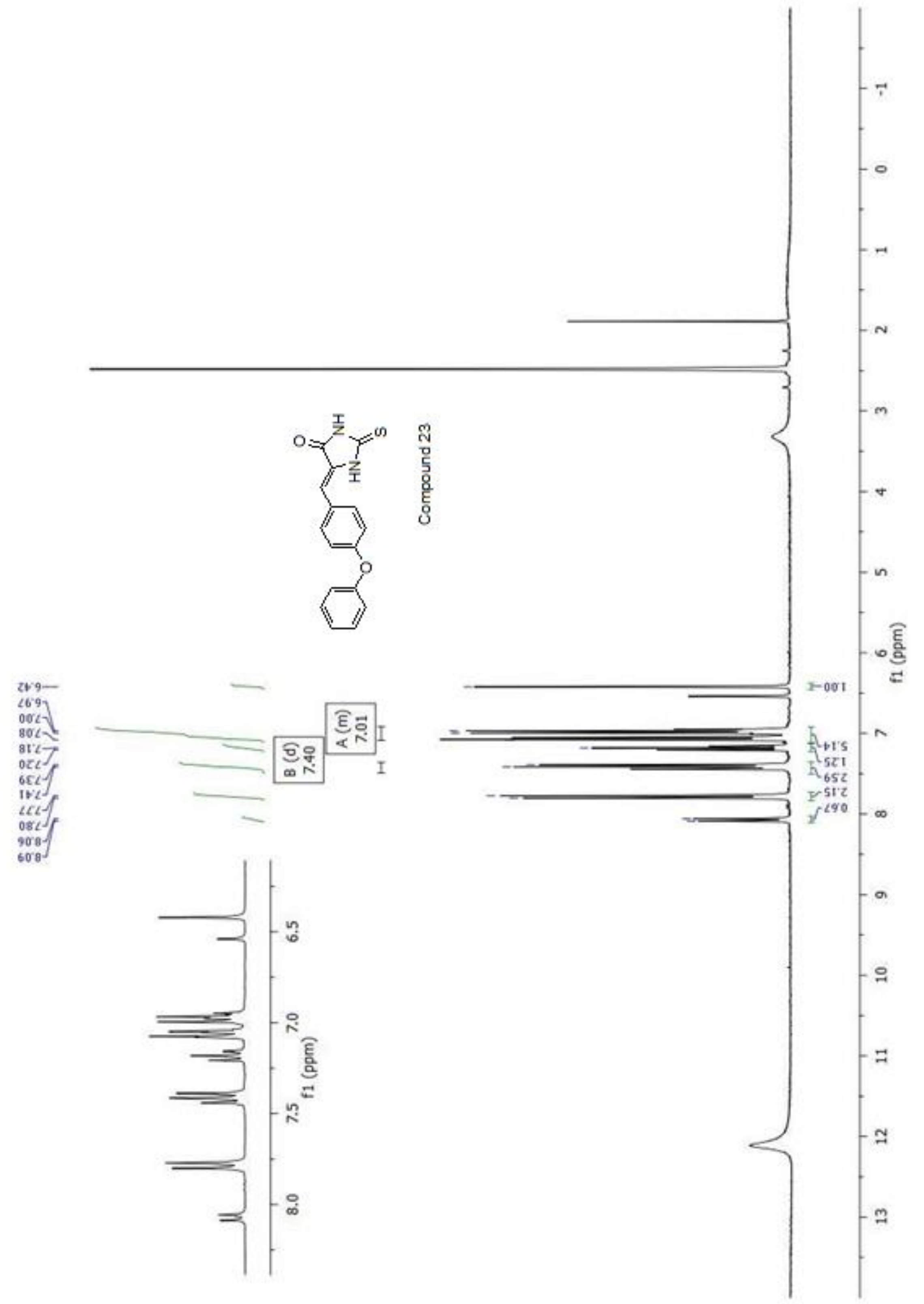

Compound 31

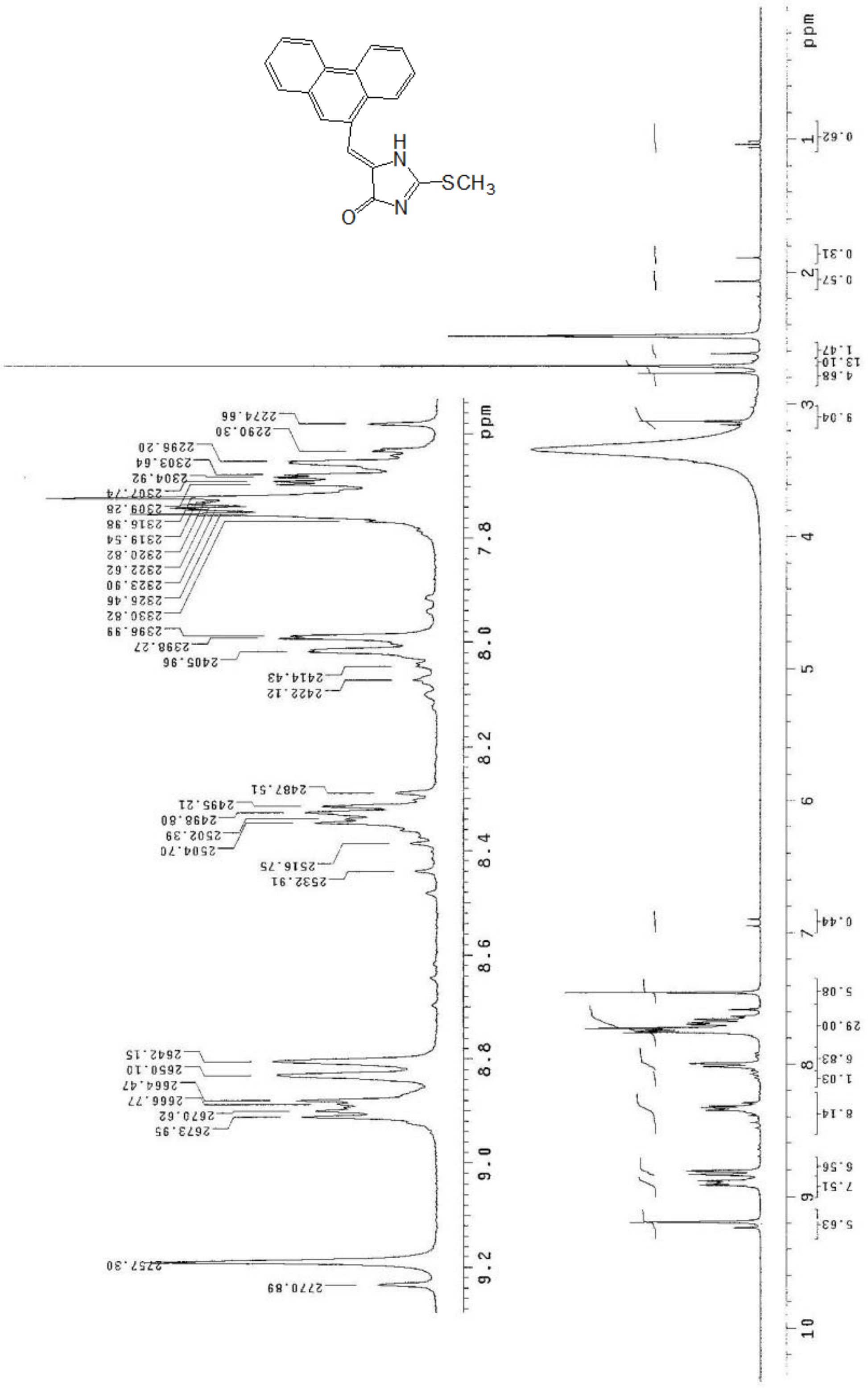

Compound 33

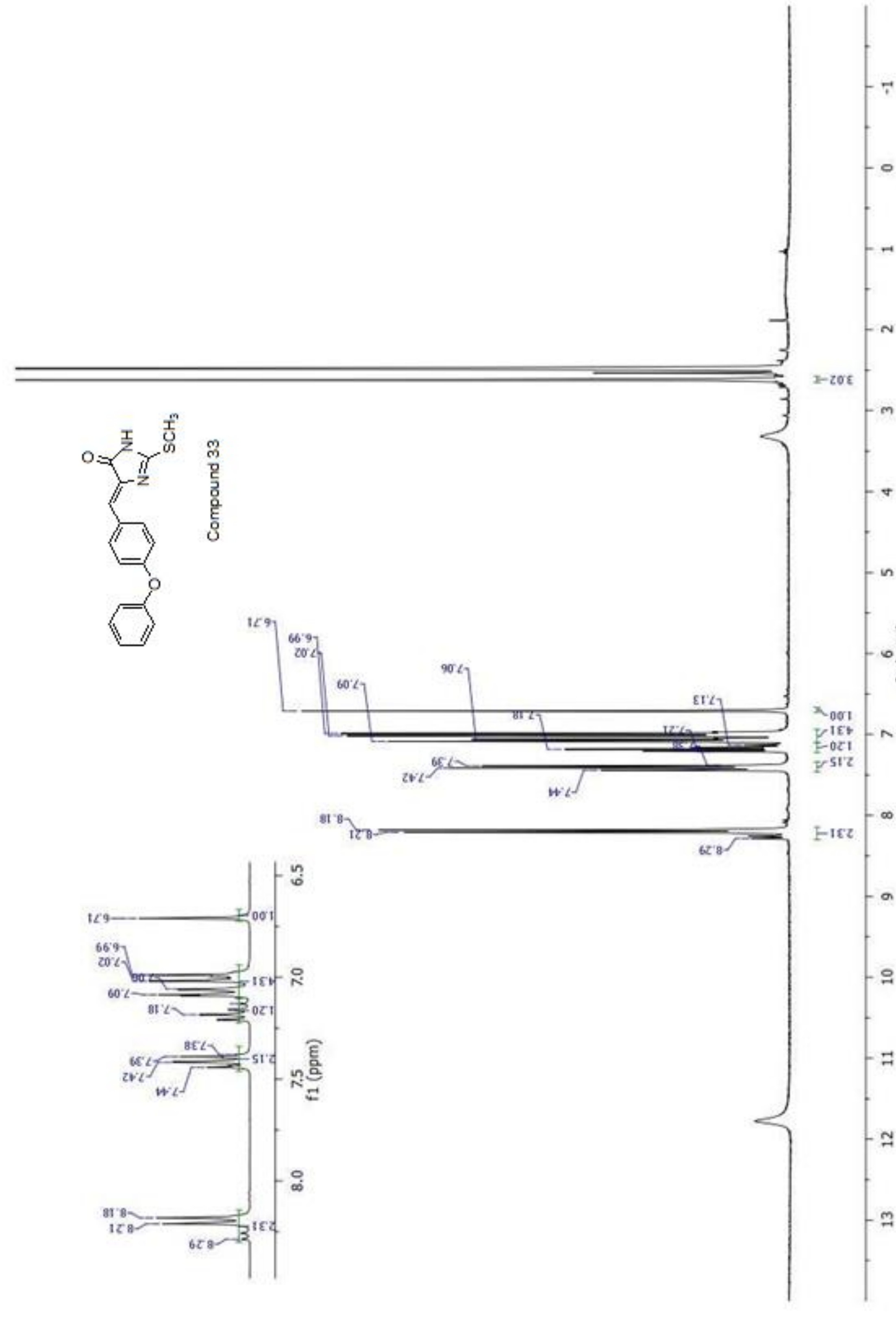

Compound 34

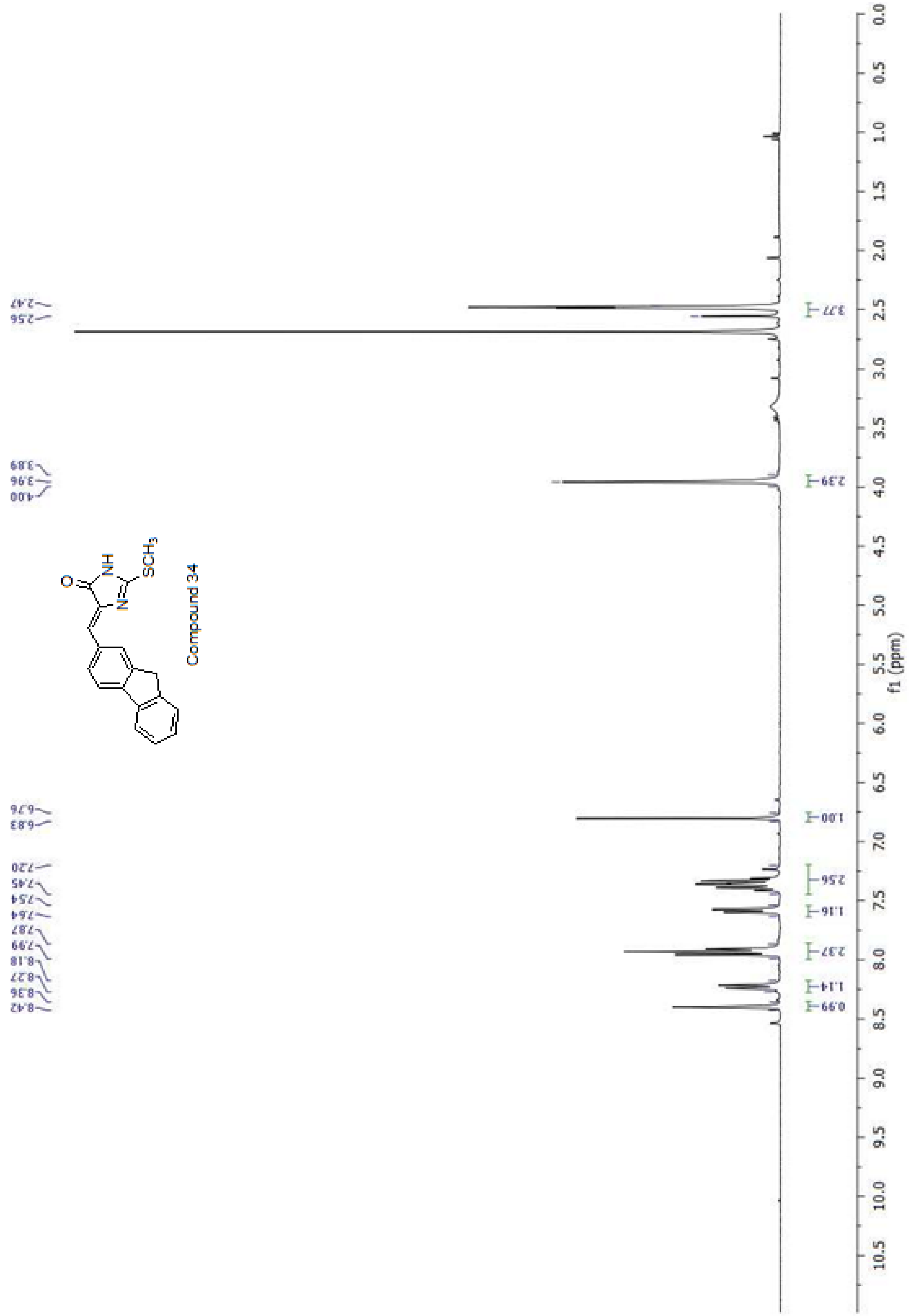

Compound 37

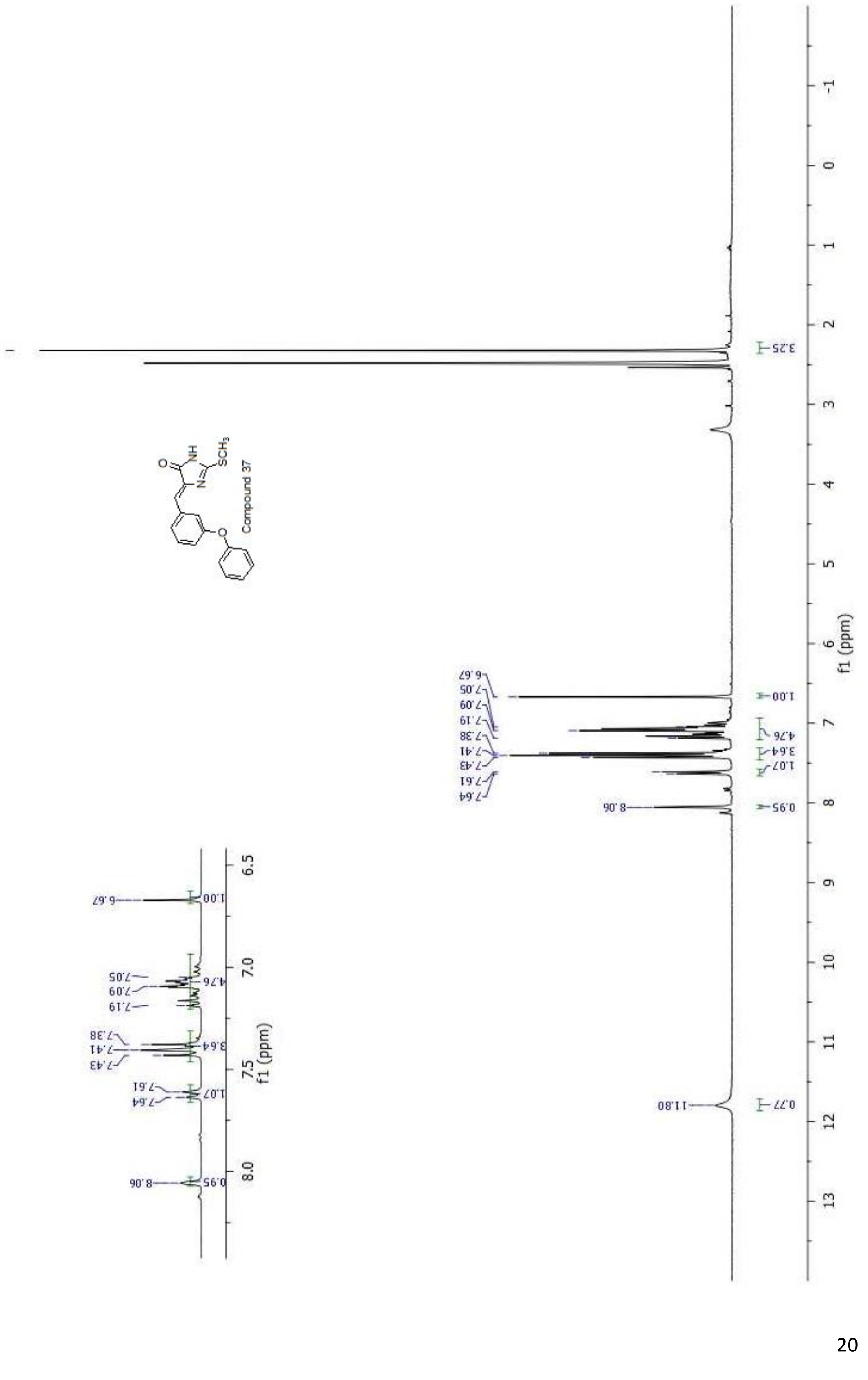
Compound 37
SCH3
NH
N
O
6.67
7.05
7.09
7.19
7.38
7.41
7.43
7.61
7.64
8.06
11.80
1.00
4.76
3.64
1.07
0.95
0.77
3.25
f1 (ppm)

13 C NMRs of selected compounds

Compound 12

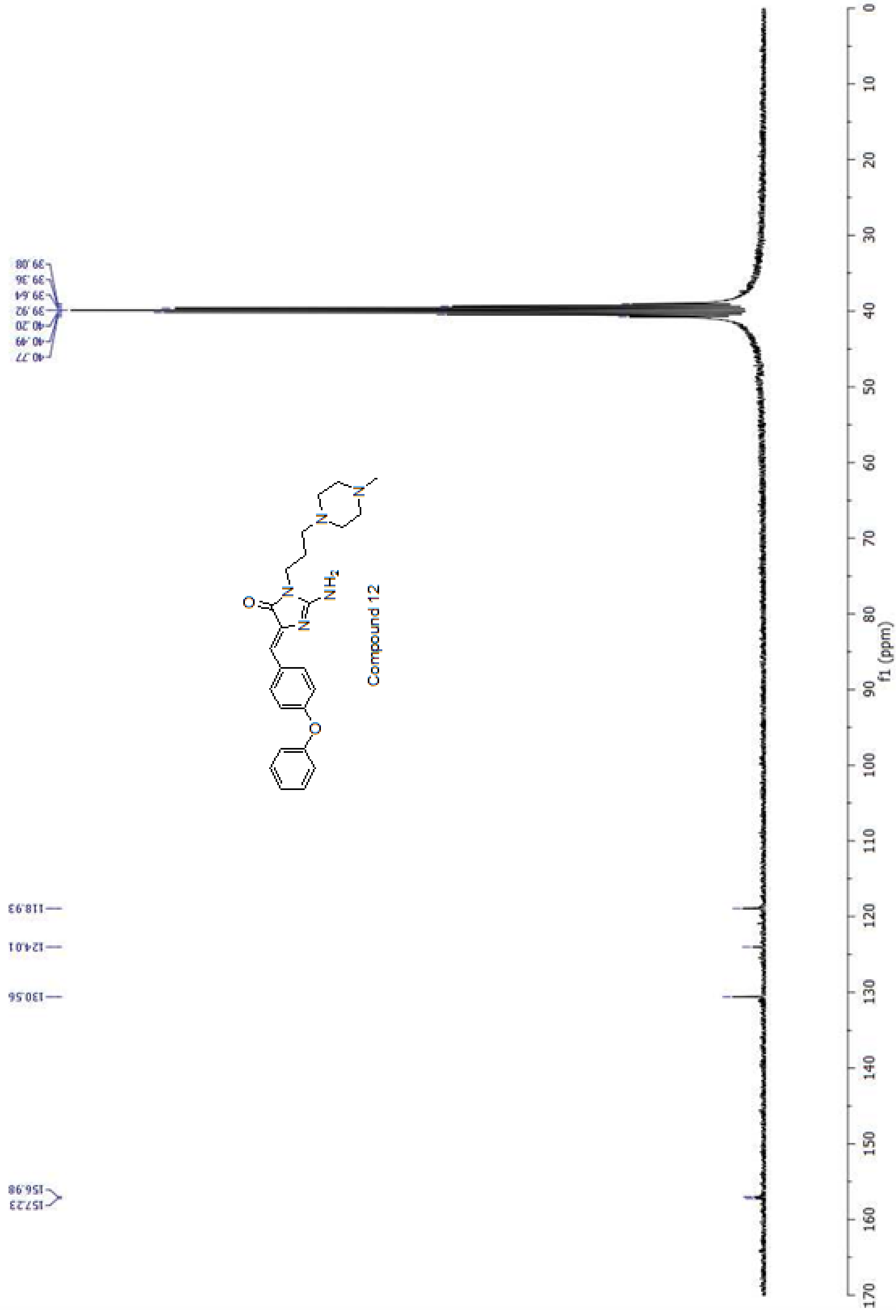

Compound 13

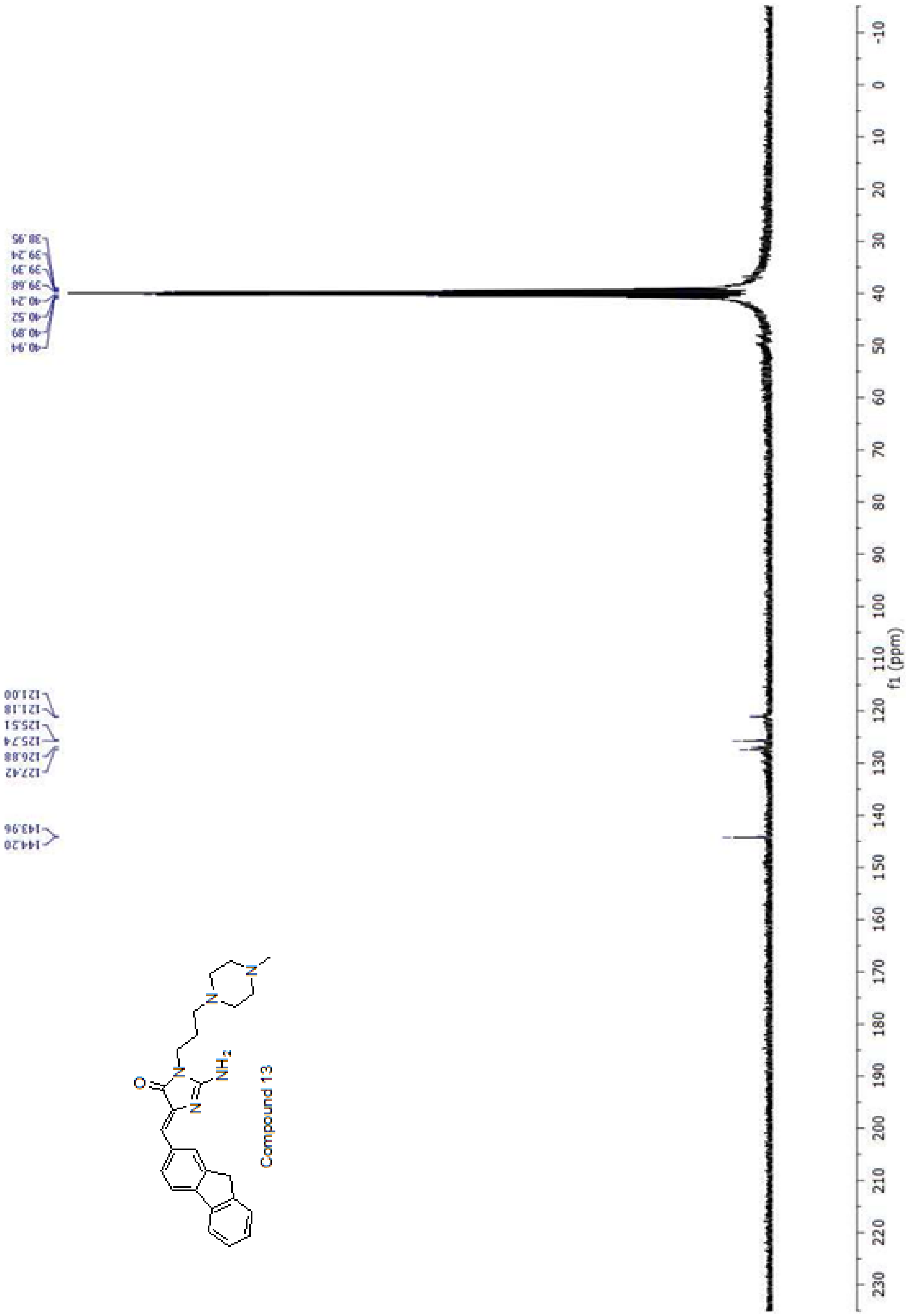

38.95
39.24
39.39
39.68
40.24
40.52
40.89
40.94
121.00
121.18
125.51
125.74
126.88
127.42
143.96
144.20
f1 (ppm)
Compound 13

Compound 14

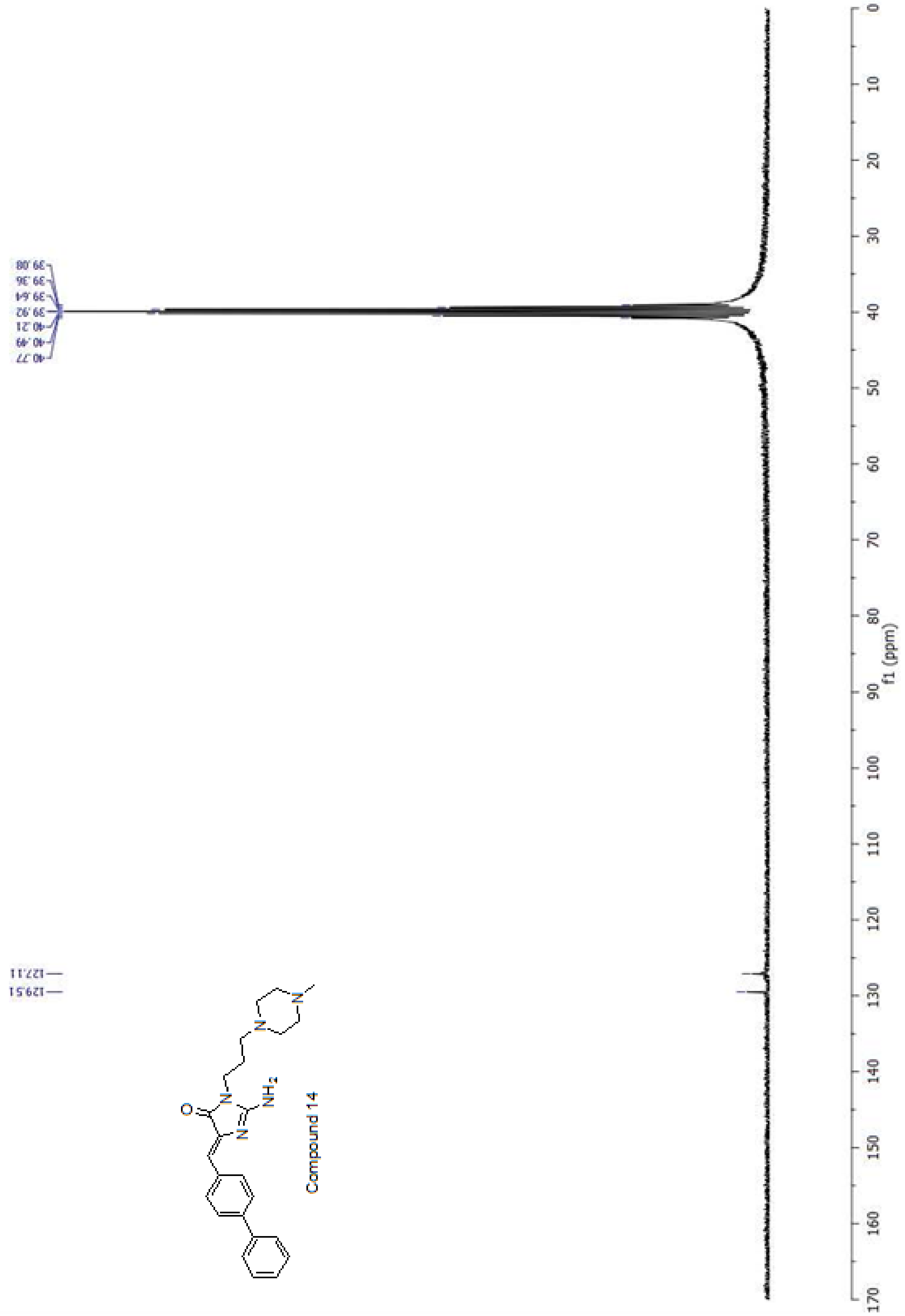
39.08
39.36
39.64
39.92
40.21
40.49
40.77
127.11
129.51
Compound 14
f1 (ppm)

Compound 15

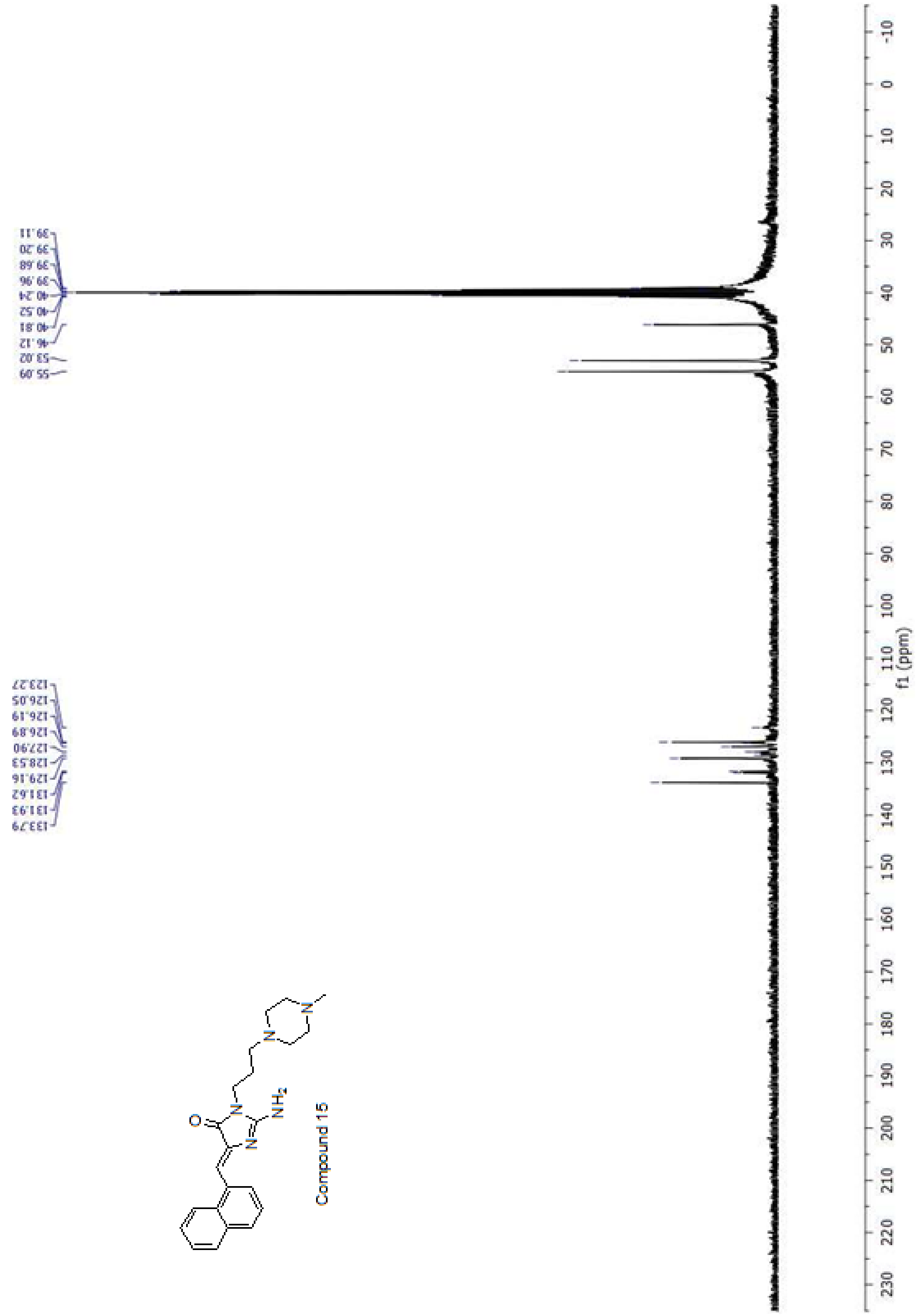
39.11
39.20
39.68
39.96
40.24
40.52
40.81
46.12
53.02
55.09
123.27
126.05
126.19
126.89
127.90
128.53
129.16
131.62
131.93
133.79
f1 (ppm)
O
N
N
NH2
N
N
Compound 15

Compound 16

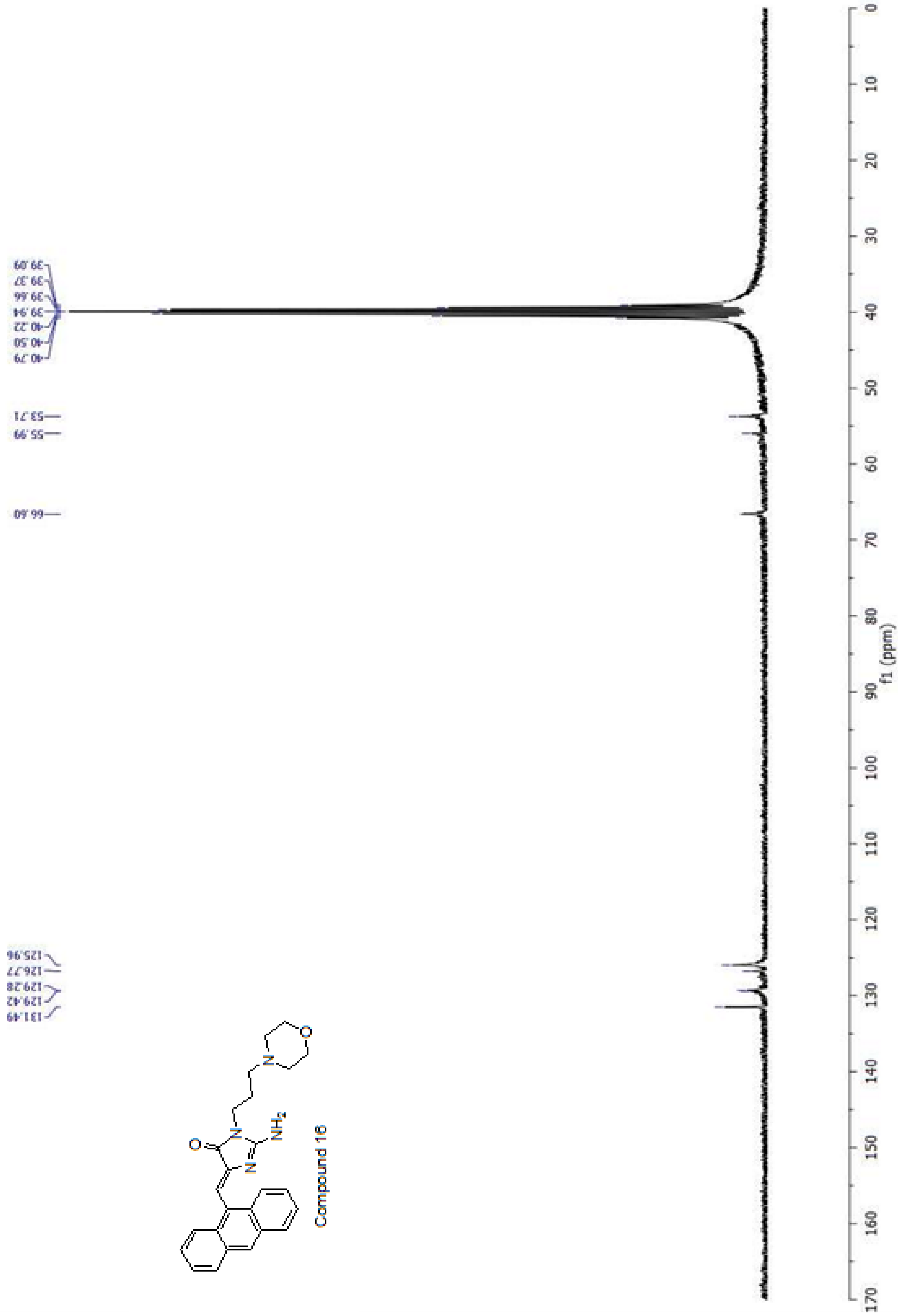
39.09
39.37
39.66
39.94
40.22
40.50
40.79
53.71
55.99
66.40
125.96
126.77
129.28
129.42
131.49
Compound 16
f1 (ppm)

Compound 17

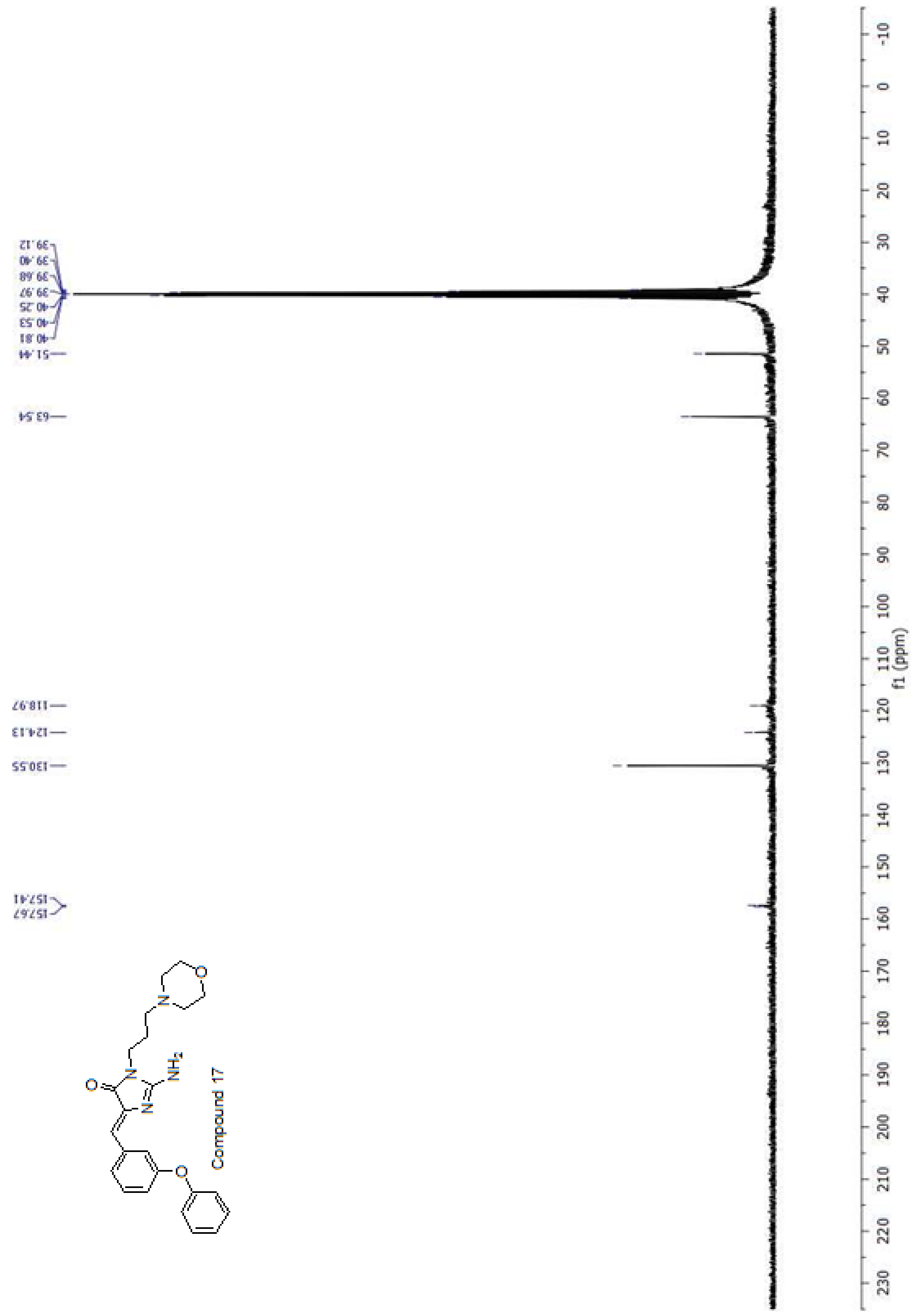

39.12
39.40
39.68
39.97
40.25
40.53
40.81
51.44
63.54
118.97
124.13
130.55
157.41
157.67
f1 (ppm)
Compound 17

Studies in silico

Ligand interaction diagrams for poses obtained in docking; grid centering at S240.

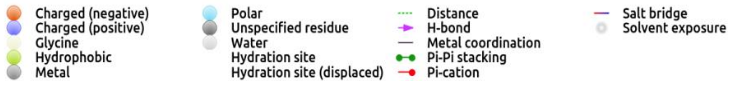

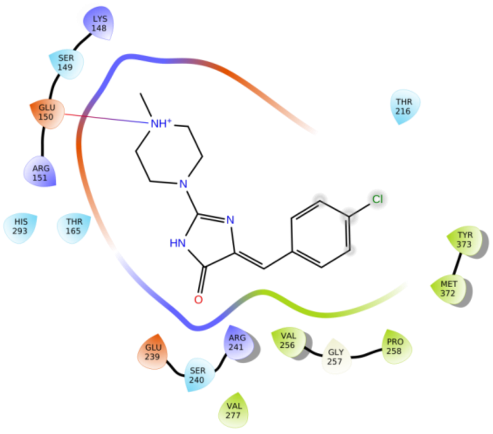

Compound 6

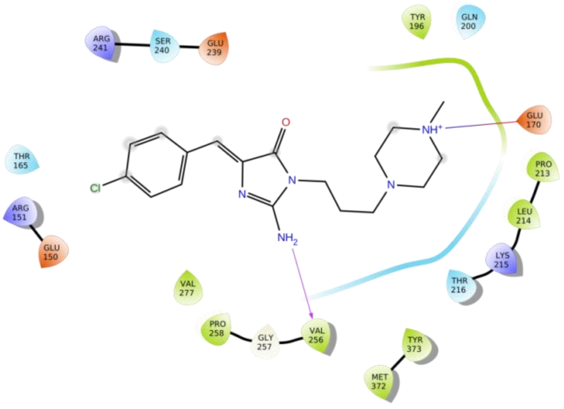

Compound 7

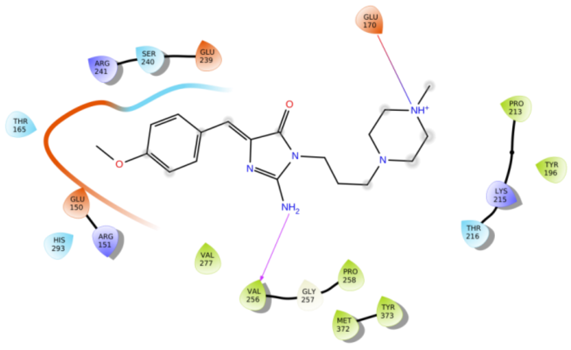

Compound 8

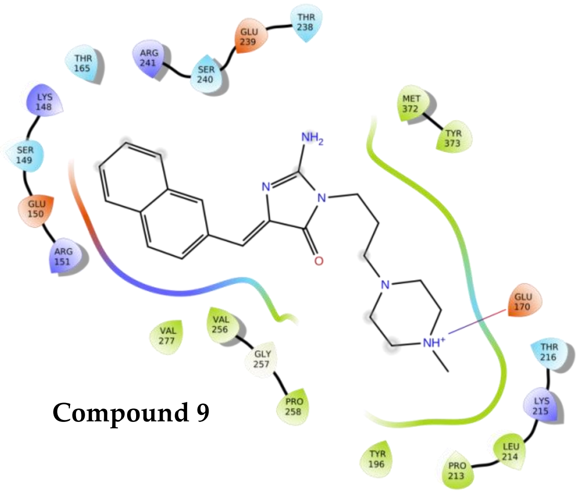

Compound 9

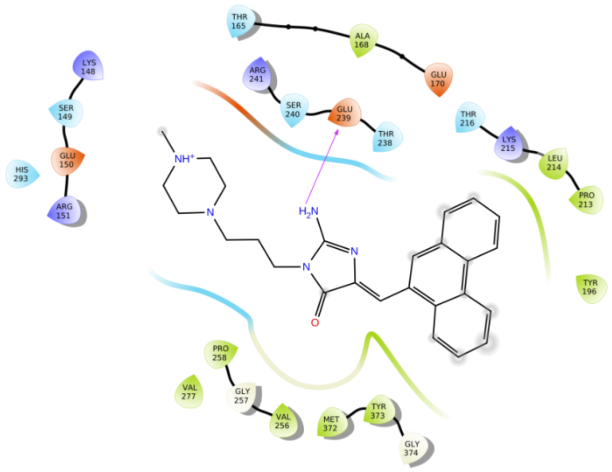

Compound 10

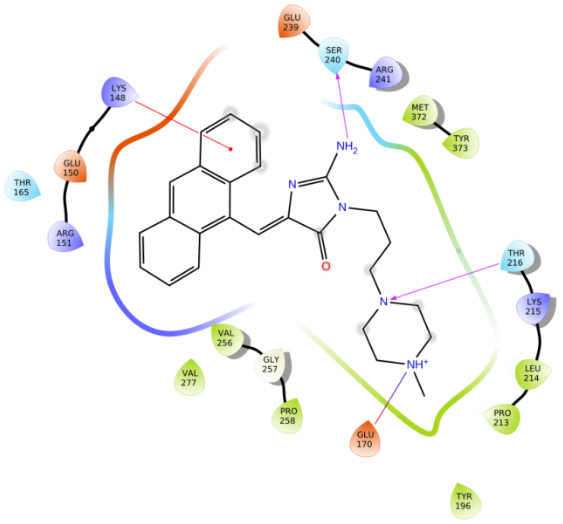

Compound 11

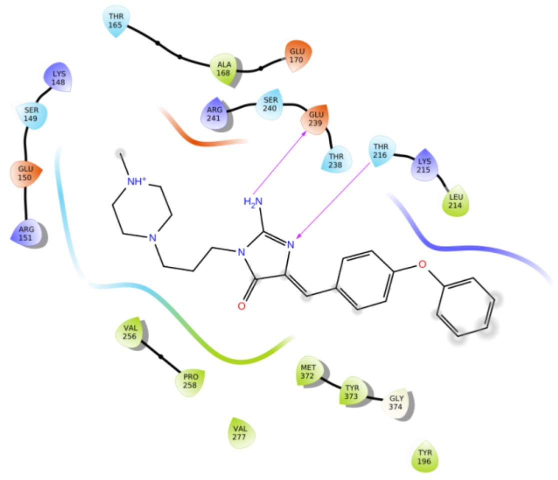

Compound 12

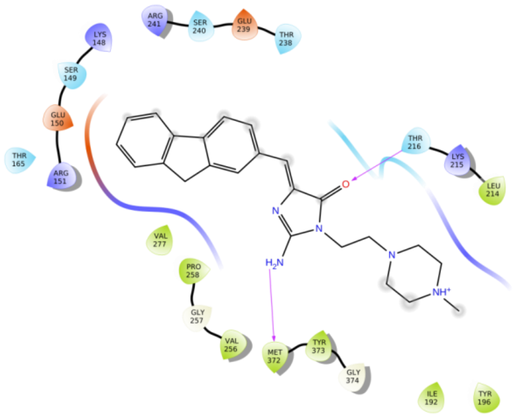

Compound 13

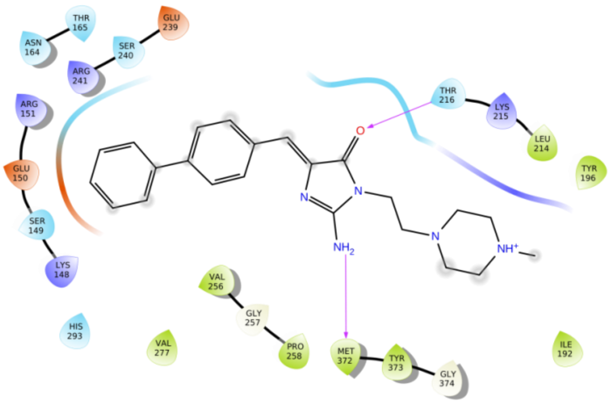

Compound 14

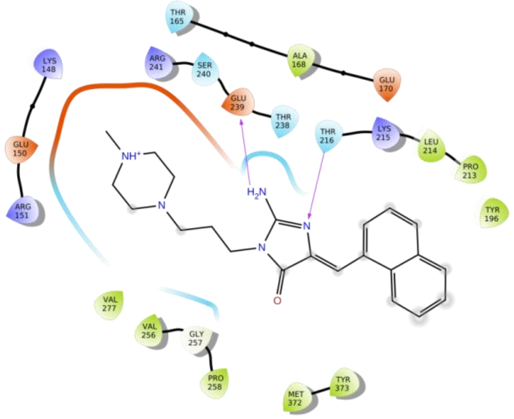

Compound 15

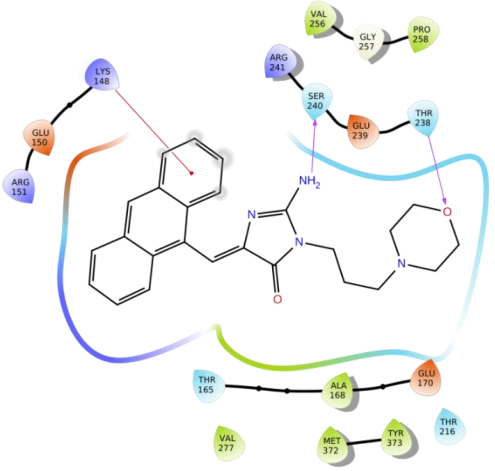

Compound 16

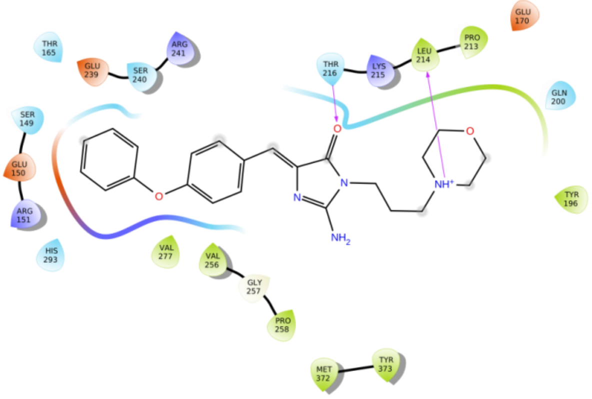

Compound 17

Ligand interaction diagrams for poses obtained in docking; grid centering at S403.

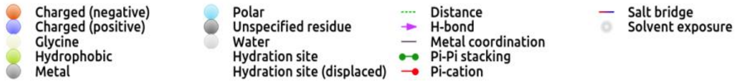

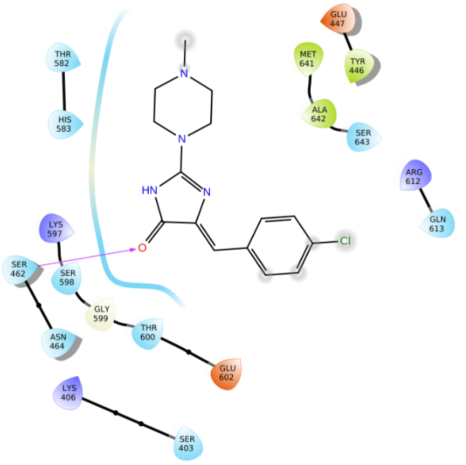

Compound 6

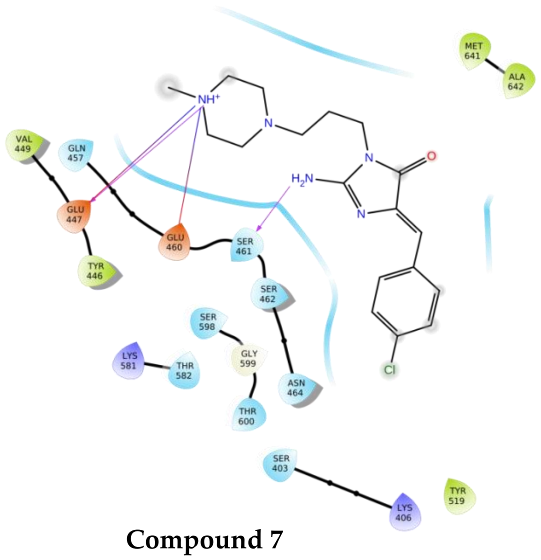

Compound 7

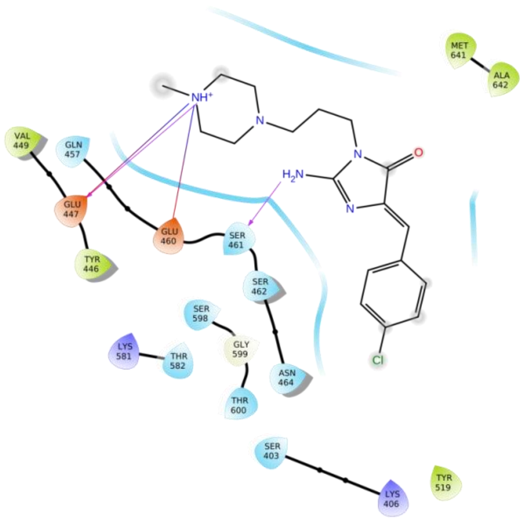

Compound 8

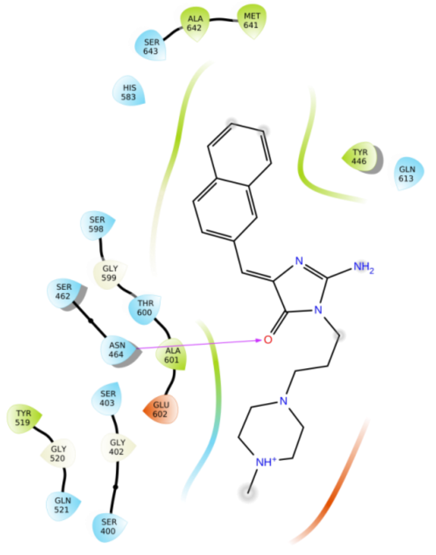

Compound 9

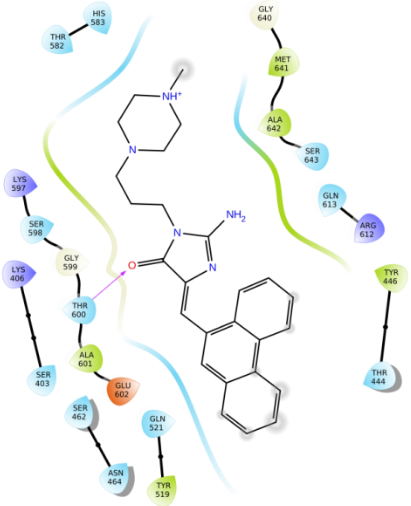

Compound 10

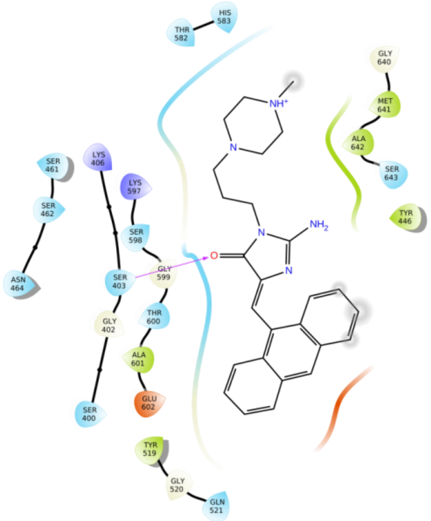

Compound 11

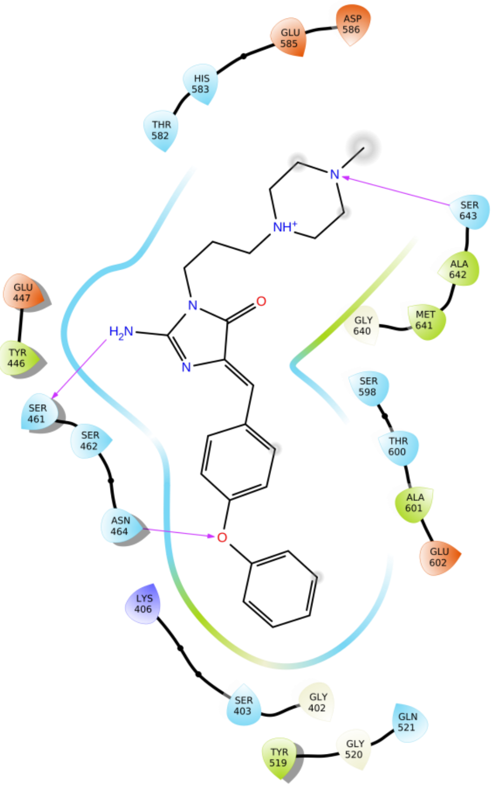

Compound 12

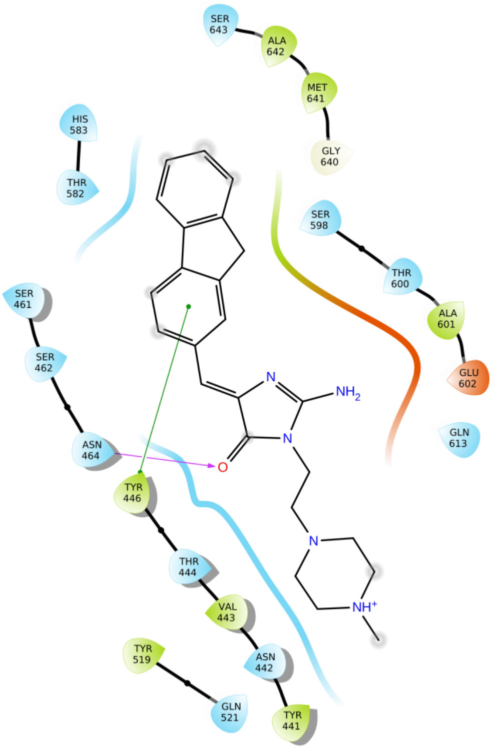

Compound 13

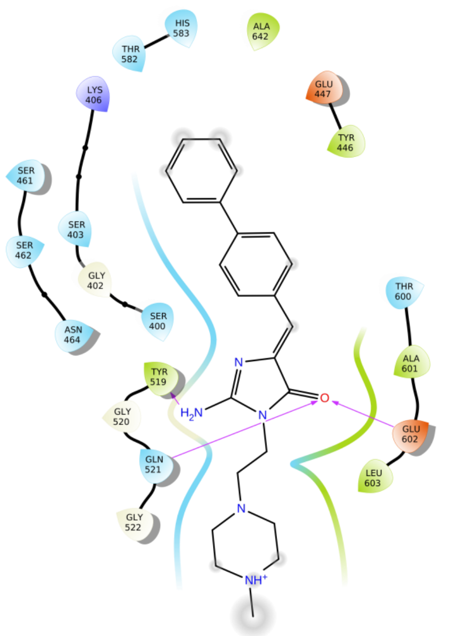

Compound 14

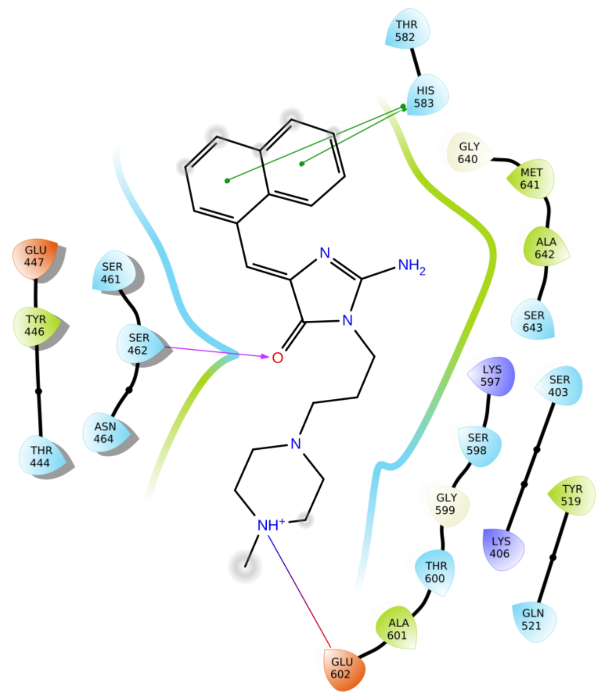

Compound 15

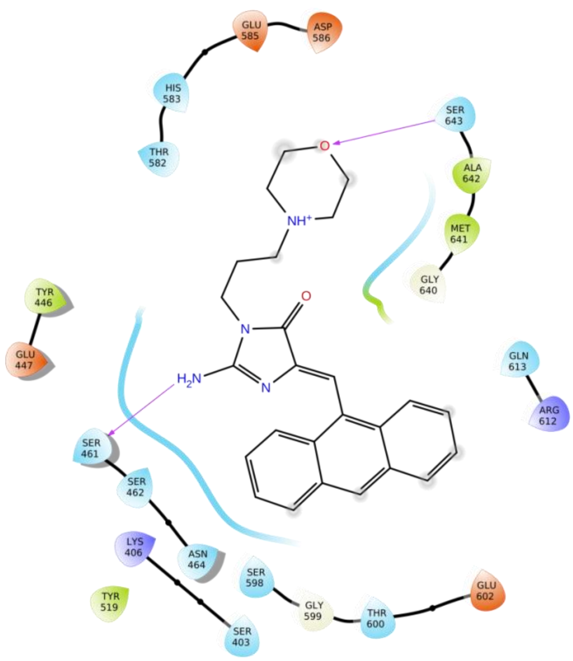

Compound 16

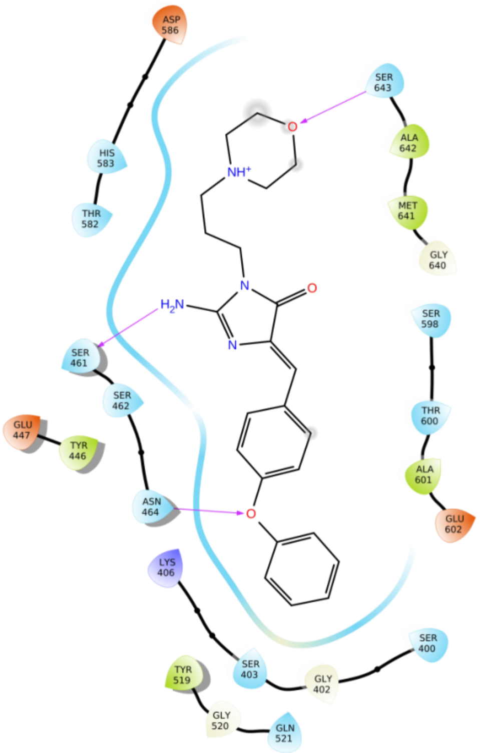

Compound 17

Simulation interaction diagrams from molecular dynamic simulations; grid centering at S240.

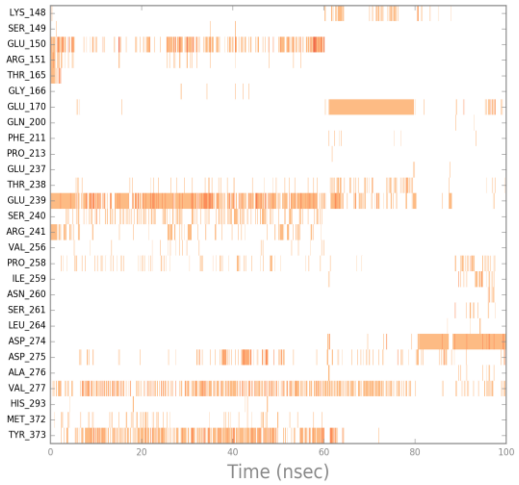

Compound 6

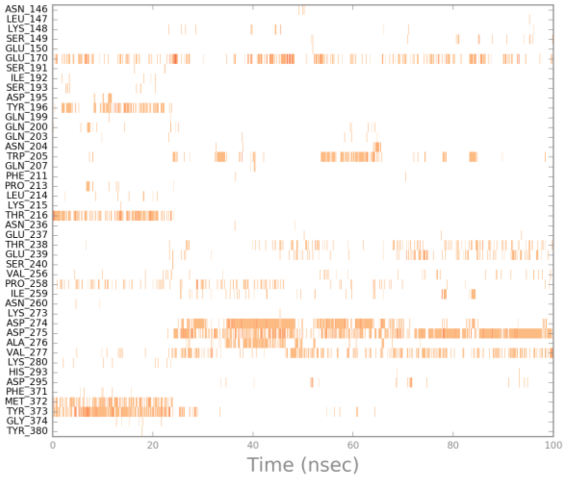

Compound 7

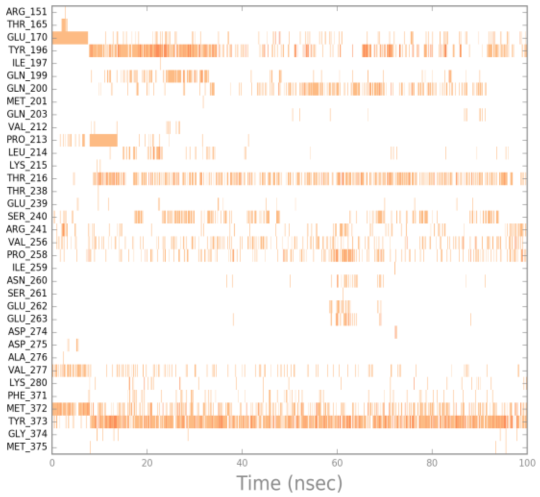

Compound 8

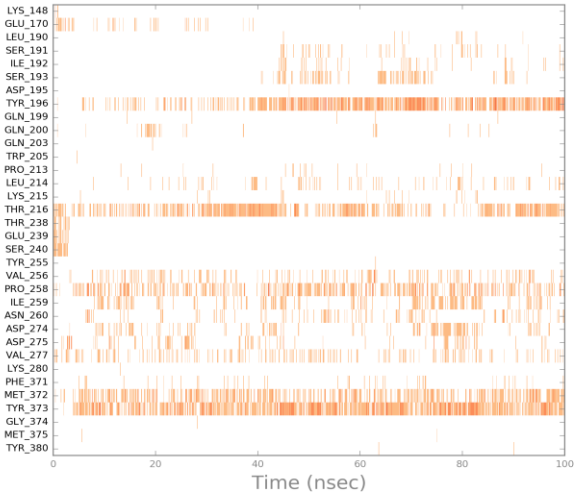

Compound 9

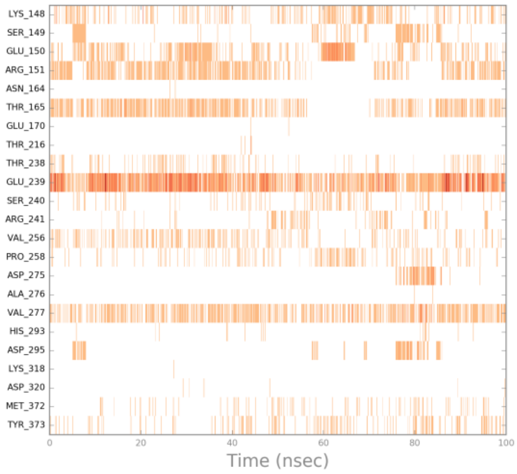

Compound 10

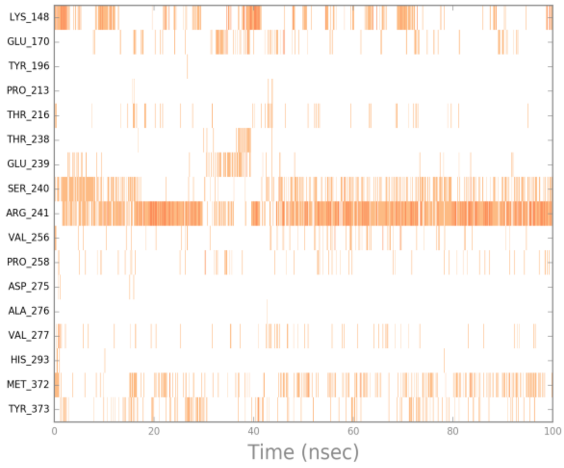

Compound 11

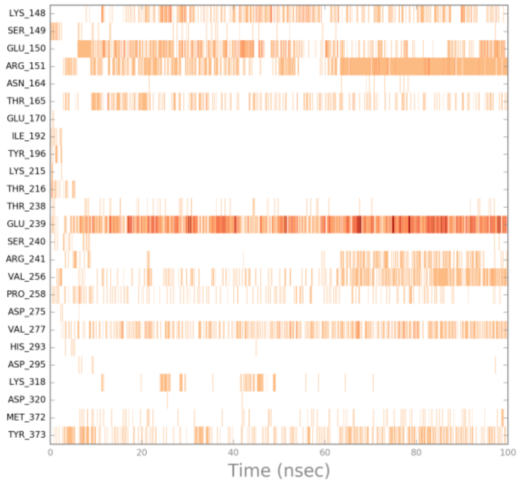

Compound 12

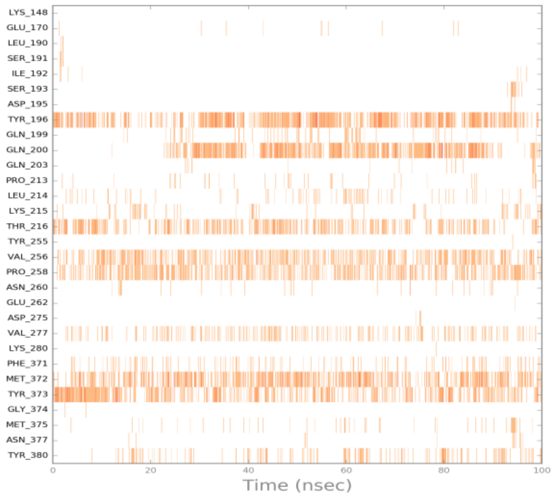

Compound 13

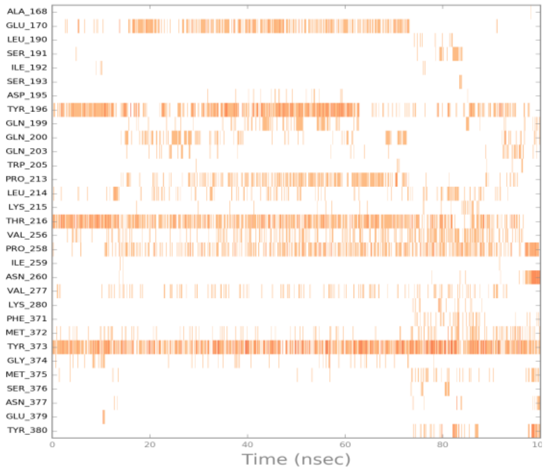

Compound 14

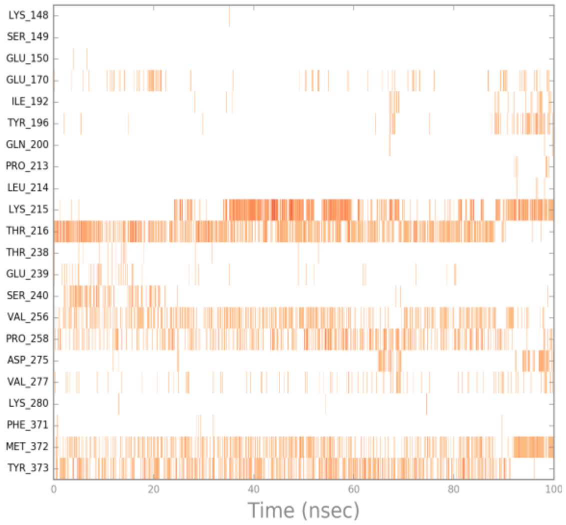

Compound 15

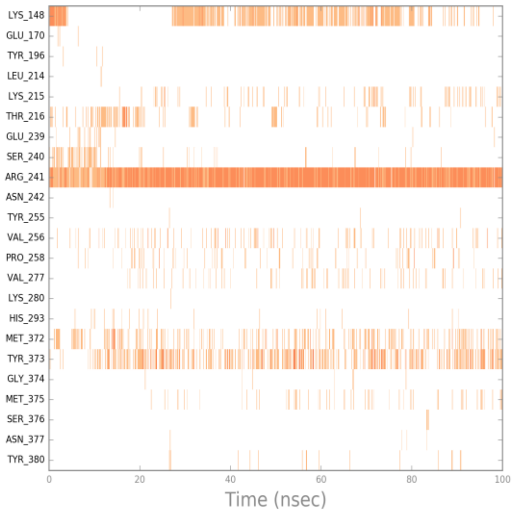

Compound 16

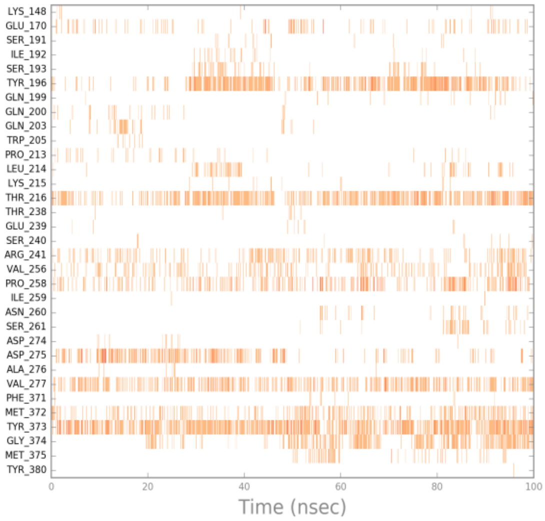

Compound 17

Simulation interaction diagrams from molecular dynamic simulations; grid centering at S403.

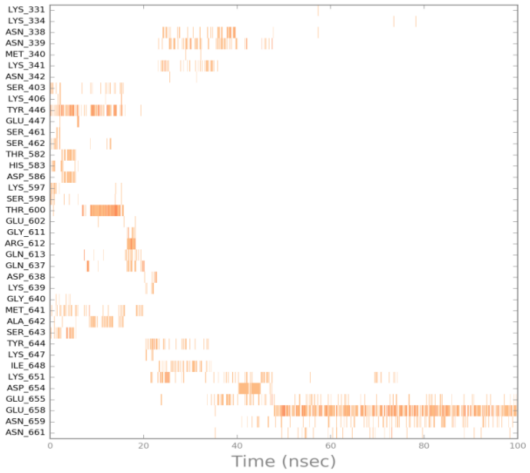

Compound 6

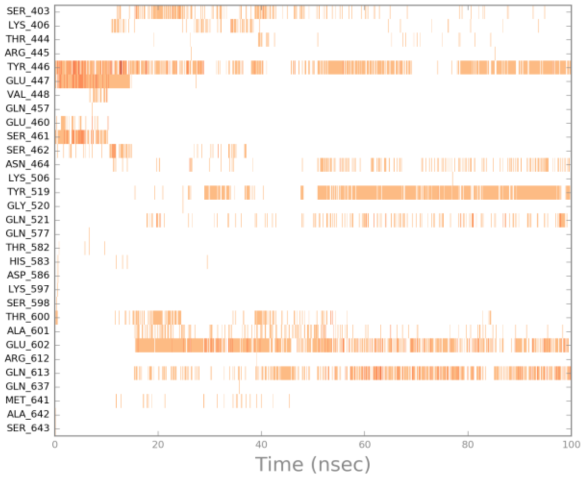

Compound 7

Compound 8 diffused away from the protein during simulation.

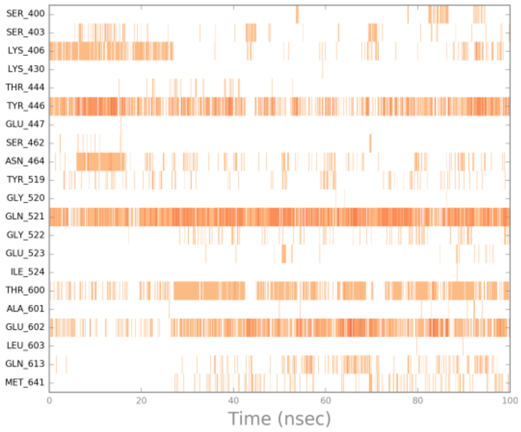

Compound 9

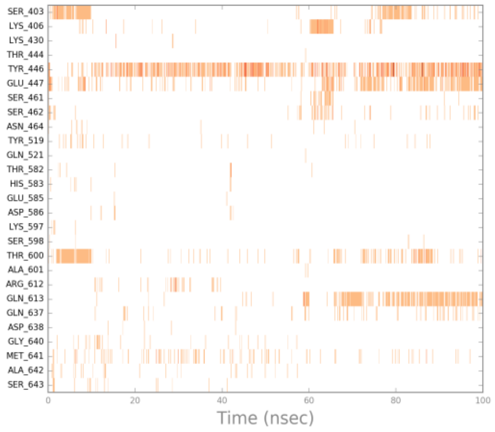

Compound 10

Compound 11 diffused away from the protein during simulation.

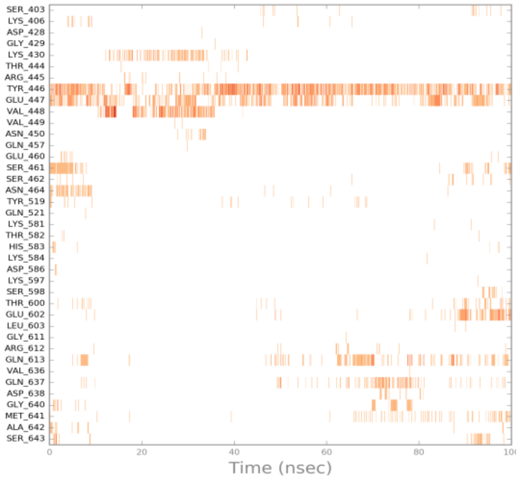

Compound 12

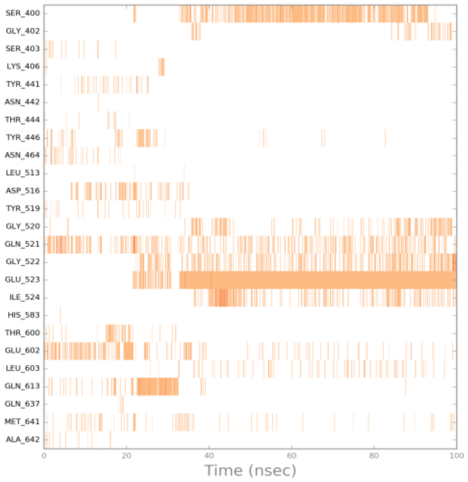

Compound 13

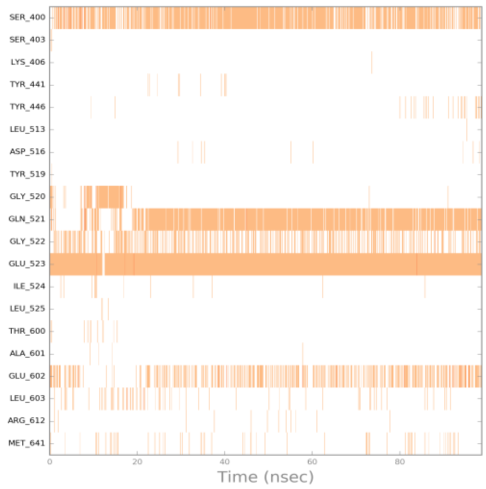

Compound 14

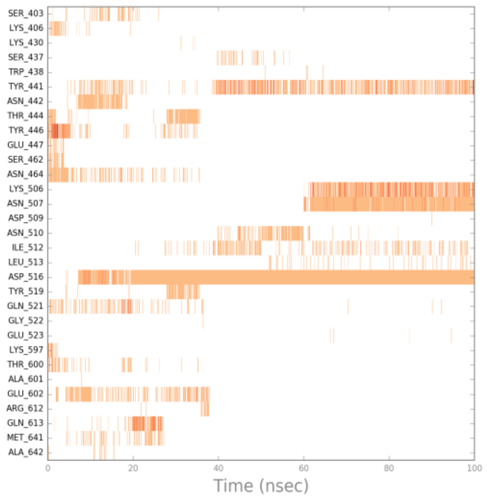

Compound 15

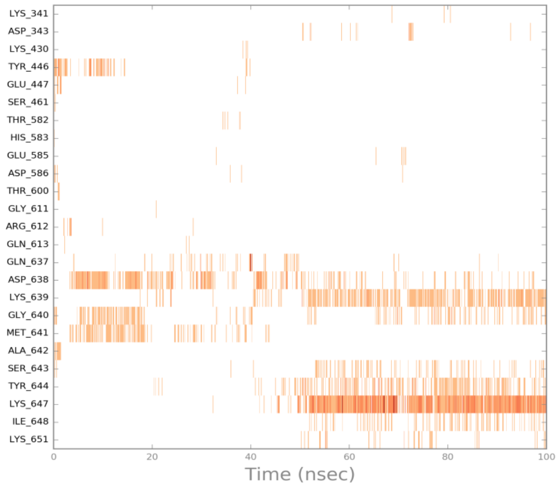

Compound 16

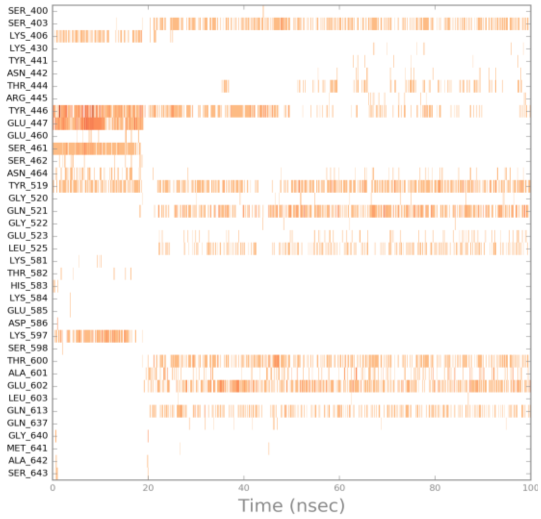

Compound 17
